# Genome-Wide Analysis of Small RNA and Novel MicroRNA Discovery in Human Acute Lymphoblastic Leukemia Based on Extensive Sequencing Approach

**DOI:** 10.1371/journal.pone.0006849

**Published:** 2009-09-02

**Authors:** Hua Zhang, Jian-Hua Yang, Yu-Sheng Zheng, Peng Zhang, Xiao Chen, Jun Wu, Ling Xu, Xue-Qun Luo, Zhi-Yong Ke, Hui Zhou, Liang-Hu Qu, Yue-Qin Chen

**Affiliations:** 1 Key Laboratory of Gene Engineering of the Ministry of Education, State Key Laboratory for Biocontrol, Sun Yat-sen University, Guangzhou, People's Republic of China; 2 Institute of Pathology and Southwest Cancer Center, Southwest Hospital, Third Military Medical University, Chongqing, People's Republic of China; 3 The Second Affiliated Hospital of Sun Yat-sen University, Guangzhou, People's Republic of China; 4 The First Affiliated Hospital of Sun Yat-sen University, Guangzhou, People's Republic of China; East Carolina University, United States of America

## Abstract

**Background:**

MicroRNAs (miRNAs) have been proved to play an important role in various cellular processes and function as tumor suppressors or oncogenes in cancers including leukemia. The identification of a large number of novel miRNAs and other small regulatory RNAs will provide valuable insights into the roles they play in tumorgenesis.

**Methodology/Principal Findings:**

To gain further understanding of the role of miRNAs relevant to acute lymphoblastic leukemia (ALL), we employed the sequencing-by-synthesis (SBS) strategy to sequence small RNA libraries prepared from ALL patients and normal donors. In total we identified 159 novel miRNAs and 116 novel miRNA*s from both libraries. Among the 159 novel miRNAs, 42 were identified with high stringency in our data set. Furthermore, we demonstrated the different expression patterns of 20 newly identified and several known miRNAs between ALL patients and normal donors, suggesting these miRNAs may be associated with ALL and could constitute an ALL-specific miRNA signature. Interestingly, GO “biological process” classifications revealed that a set of significantly abnormally expressed miRNAs are associated with disease relapse, which implies that these dysregulated miRNAs might promote the progression of ALL by regulating genes involved in the pathway of the disease development.

**Conclusion/Significance:**

The study presents a comprehensive picture of the expression of small RNAs in human acute lymphoblastic leukemia and highlights novel and known miRNAs differentially expressed between ALL patients and normal donors. To our knowledge, this is the first study to look at genome-wide known and novel miRNA expression patterns in in human acute lymphoblastic leukemia. Our data revealed that these deregulated miRNAs may be associated with ALL or the onset of relapse.

## Introduction

MicroRNAs (miRNAs) are a class of endogenous noncoding RNAs, between 19 to 25 nucleotides in size, which can regulate gene expression at either the transcriptional or post-transcriptional level [1]. Studies have shown that miRNAs play an important role in development and various cellular processes, such as differentiation, growth, and death [2]. A connection between miRNAs and cancers has also been elucidated [3]. It has been shown that about 50% of annotated human miRNAs are located at fragile sites and genomic regions involved in cancers [4]. Other data indicates that miRNAs function as both tumor suppressors and oncogenes in tumorigenesis and cancer-specific miRNA expression signatures have been identified in many cancers [4]–[7]. Identification of a large number of novel miRNAs and other small regulatory RNAs is critical to provide valuable insights into the role these may play in tumorgenesis.

Recently, the sequencing-based method has been widely applied for identifying and profiling novel miRNAs candidates. Studies on novel miRNAs based on the sequencing method have been reported in chicken, human embryonic stem cells, solid cancers, etc. [8]–[14]. In leukemia, Marton et al. [15] used a cloning-based sequencing approach to study small RNAs from chronic lymphocytic leukemia (CLL) patients and five novel miRNA candidates were identified, which could be relevant in CLL pathogenesis. Another study examined miRNA expression profiles and isolated many novel miRNAs candidates from leukemia cells mainly present in acute myeloid leukemia (AML) by means of miRNA amplification and sequencing [16]. Their results suggest that there are still many novel miRNAs existing in leukemia cells. Recently, the Illumina massively parallel sequencing platform was used to carry out an in-depth analysis of the miRNA transcriptome in a murine AML leukemia progression model and 55 novel miRNAs were identified, some of which could be relevant to the pathogenesis of AML [17], indicating that the high-throughput sequencing method can be used as a new and powerful tool to identify unannotated novel miRNA candidates which are lowly abundant or nonconserved but relevant to the diseased state.

Acute lymphoblastic leukemia (ALL) is a malignant disorder of lymphoid progenitor cells and characterized by chromosomal abnormalities, which occurs at all ages but with peak prevalence between the ages of 2 and 5 years [18]. With modern treatment strategies using risk-adapted combination chemotherapy, the cure rates of childhood ALL are almost 80% [19]. However, 20–30% of children still relapse and conventional intensive chemotherapy can only cure up to 30% of children who have relapsed [20]–[21]. As treatment of relapsed disease remains a challenging, the accurate assignment of individual patients to risk groups is a critical issue for optimal outcome[20], [22], [23]. Although microRNA expression signatures associated with cytogenetics and the clinical outcome of ALL have been reported [24]–[27], these efforts are limited as they are mainly restricted to the detection and profiling of previously identified miRNA sequences.

To gain a more complete and unbiased view of the small RNA transcriptome and further understand the role of miRNAs relevant to ALL, we employed a sequencing-by-synthesis (SBS) strategy to globally study small RNAs, especially miRNAs that have thus far been proven difficult to find using traditional cloning and in silico predictions. We present a comprehensive picture of the expression of small RNAs and have identified 159 novel miRNAs in total. Notably, the unique expression patterns in ALL patients and GO analysis suggest a set of miRNAs may be associated with disease relapse, which provides valuable insight into the pathogenesis of ALL relapse.

## Results

### Annotation of small RNAs and identification of novel miRNA genes from patient group and normal donor

**Figure 1:**
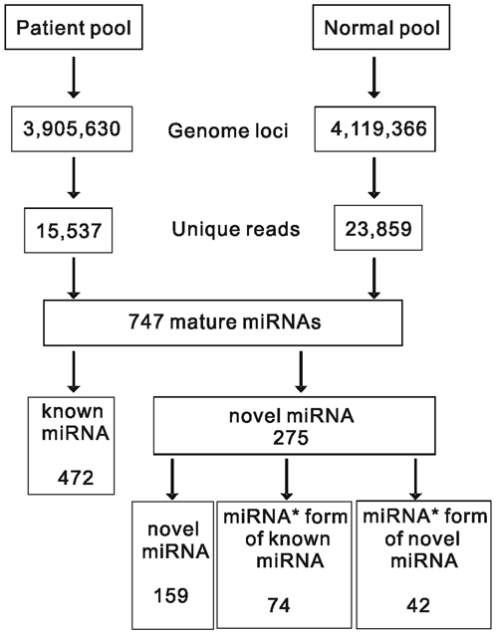
Schematic representation of small RNA library sequencing and subsequent bioinformatic analysis.

**Figure 2:**
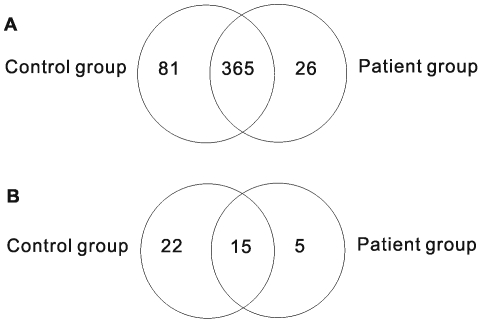
Venn diagram of the known miRNAs and novel miRNA*(star)s of the known miRNAs identified from the control group and the ALL patient group. (A) Known miRNAs unique in each group and shared by both. (B) MiRNA*s of known miRNAs unique in each group and shared by both.

To discover additional miRNAs that have escaped detection in previous studies associated with ALL [24]–[27], we employed SBS strategy to sequence two small RNA libraries of pooled bone marrows from a patient group (P) and a normal donor group (N). This yielded 4,418,887 (P) and 4,361,324 (N) reads, respectively. After computer filtering to remove ambiguous reads, 831,728 (P) and 1,993,962 (N) sequence reads were obtained corresponding to 60,460 (P) and 113,414 (N) unique reads (18 nt∼30 nt). Unique sequences were mapped to the human genome (Mar. 2006, version hg18, NCBI Build 36.1) using MegaBLAST [28] (version 2.2.18), and those with perfect matches across their entire length were retained. Consequently, a total of 15,537 and 23,859 unique sequences represented by 608,358 (P) and 1,327,742 (N) reads were perfectly matched to 3,905,630 and 4,119,366 genome loci, respectively. The schematic representation of small RNA library sequencing and subsequent bioinformatic analysis is shown in **Fig. 1**. These sequences were annotated based on their overlap with publicly available genome annotations **(Table S1)** and a total of 472 known miRNAs were identified with a greater total number of miRNAs expressed in the normal donor group than in the patient group **(** **Fig. 2A** **)**. By analyzing the precursor sequence of miRNAs, we identified a total of 74 novel miRNA*(star)s **(Table S2)** in both groups. Mature miRNAs are processed from the stem of a hairpin precursor, and the miRNA*(star) sequence corresponds to the section of this hairpin that remains complementary to the mature form with approximately 2-nucleotide 3′ overhangs [29], [30]. Among them, we found that some miRNA*s such as hsa-miR-766* were significantly up-regulated in the patient group, whilst some miRNA*s such as hsa-miR-1307* were significantly down-regulated in the patient group with fold changes >2.0 and a *p*-value of <0.001, suggesting miRNA*s could be involved in the progression of ALL.

To identify novel miRNAs among the unclassified sequences in our libraries, we removed all known miRNAs, other small RNAs and genomic repeats. A total of 12,798 unique sequences (corresponding to 55,128 reads) in the normal donor library and 7,356 (corresponding to 29,599 reads) in the ALL library were unannotated. We next evaluated reads that fell within potential miRNA-like hairpins considering the criteria: 1) z-score less than −3.0, this discriminate significantly miRNAs from other structured RNAs [31]–[33]. 2) A total number of reads from two libraries at least three reads in harpins or 3) the presence of reads corresponding to the predicted miRNA* species [13], [34]. Novel candidate miRNAs had to satisfy criteria 1, and one of criteria 2 and 3. One hundred and fifty nine novel miRNAs**(Fig. S1)** and 116 novel miRNA*s were identified from the two libraries. As described by Ruby et al. [13], the observation of both a candidate miRNA and a corresponding candidate miRNA* in a set of reads provides particularly compelling evidence for Dicer-like processing from an RNA hairpin. Therefore, 42 out of 159 novel candidate miRNAs met the criteria. Among the 42 novel miRNAs meeting the highly stringenct criteria, 22 and 5 novel miRNAs are unique in normal donor and ALL patient groups, respectively. The other 15 novel miRNAs are shared by both groups **(** **Table 1** **,** **Fig. 2B** **)**.

**Table 1:** 42 novel candidate miRNAs identified from control and patient group.

MiRNA	Sequence	MiRNA Reads C group	MiRNA Reads P group	MiRNA* Reads C group	MiRNA* Reads P group
hsa-miR-1832	TCCACATGTAAAAAAATGAATC	7	0	3	4
hsa-miR-1836	AAAAGTTATTGTGGTTTTTGCT	8	0	3	2
hsa-miR-1842	AAAACCGTCTAGTTACAGTTGT	355	4	44	0
hsa-miR-1845	ACATGGAGTTCAGGTGAGGATT	1	0	0	1
hsa-miR-1851	AGACCCATTGAGGAGAAGGTTC	4	0	1	0
hsa-miR-1852	CCTGCAACTTTGCCTGATCAGA	66	2	40	1
hsa-miR-1855	AAAAGTAATTGTTGTTCTTGCC	0	3	0	1
hsa-miR-1859	ATCCCCAGATACAATGGACAAT	103	14	121	14
hsa-miR-1866	CATCAGAATTCATGGAGGCTAGA	30	2	6	0
hsa-miR-1872	TCTCAGTAAGTGGCACTCTGTC	0	1	0	1
hsa-miR-1876	AGCTTTTGGGAATTCAGGTAG	6	1	1	1
hsa-miR-1886	GCAAAGACCACGATTACTTTT	2	0	2	0
hsa-miR-1889	TCCAGTACATATAAAGAGACTT	2	0	1	0
hsa-miR-1896	TTCAGTGTAACTCAACATTTGA	3	0	4	0
hsa-miR-1901	AATTACAGATTGTCTCAGAGAA	175	51	21	6
hsa-miR-1906	TGAGGAGATCGTCGAGGTTGG	4	0	1	0
hsa-miR-1909	TTAGCCAATTGTCCATCTTTAG	3	1	3	0
hsa-miR-1911	CACTTGTAATGGAGAACACT	2	0	0	1
hsa-miR-1915	CAGACAGCTTGCACTGACT	1	0	1	0
hsa-miR-1916	AAAATCCTTTTTGTTTTTCCAG	3	0	1	0
hsa-miR-1917	CACAAGATGCCTAGTTAAATTT	1	0	1	1
hsa-miR-1923	AAATACCACAGTTACTTTTGCA	2	0	0	1
hsa-miR-1926	TATCGTGCATATATCTACCACAT	2	0	1	3
hsa-miR-1929	TTAATATGTACTGACAAAGCGT	4	0	4	0
hsa-miR-1930	CCTCCCACTGCAGAGCCTGGGGA	1	0	0	1
hsa-miR-1932	TTGTGAAGAAAGAAATTCTTA	12	0	4	1
hsa-miR-1940	CCAGAGAAGGCTGCTCCTCACCA	1	0	0	1
hsa-miR-1941	AAGAGTTACTAGAACTATT	2	0	2	0
hsa-miR-1942	AATTTATTCTTGGTAGGTTGT	1	0	2	0
hsa-miR-1945	TGGGACTGATCTTGATGTCT	5	9	0	1
hsa-miR-1946	TTTAGTACCTATAATGTGCTAG	2	0	0	1
hsa-miR-1947	TGTTGTACTTTTTTTTTTGTTC	120	12	23	2
hsa-miR-1953	GCCCTGCCTGTTTTCTCCTTTG	1	1	1	1
hsa-miR-1955	AGCAATACTGTTACCTGAAAT	2	4	1	0
hsa-miR-1962	AGAAGGGGTGAAATTTAAACGT	7	3	1	0
hsa-miR-1963	AGAAGGGGTGAAATTTAAACGT	7	3	1	0
hsa-miR-1964	AGAAGGGGTGAAATTTAAACGT	7	3	1	0
hsa-miR-1967	TAGCTGTAGCTTTAGCAGAGC	0	1	0	1
hsa-miR-1971	TAGCCTTCAGATCTTGGTGTTT	125	11	34	0
hsa-miR-1972	TGAGACAGGCTTATGCTGCTA	0	4	0	1
hsa-miR-1975	CAAAAGTAATTGTGGTTTTTGTT	0	1	0	1
hsa-miR-1984	AATCTGAGAAGGCGCACAAGGTT	2	0	1	0

### Differentially expressed patterns of known and novel miRNAs between control and patient group

**Figure 3:**
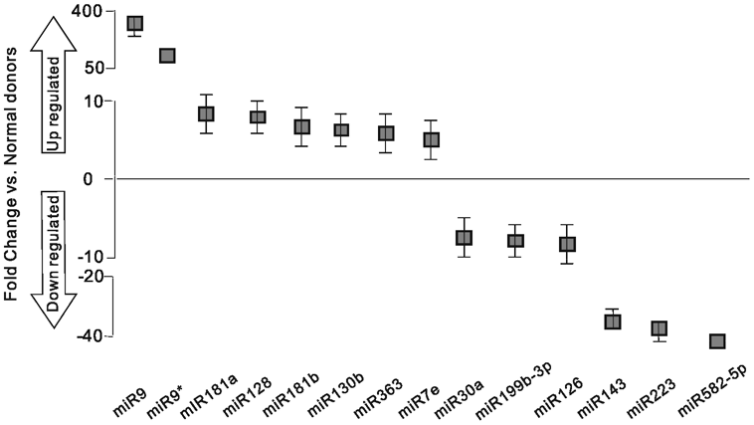
Differential expression of miRNAs in ALL patients. Box plots (median and interquartile range) show the distribution of fold change of expression in ALL patients compared with normal donor samples.

**Figure 4:**
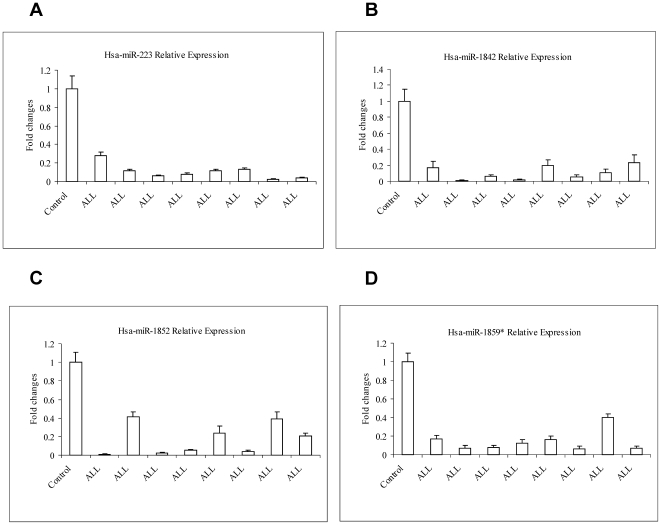
The expressions of four miRNA were validated in the patient group compared with the control group using qRT-PCR.

Using this sequencing platform, we performed a systematic miRNA expression profiling analysis of patient samples. A total of 847 miRNAs including miRNA*s (miRBase 11.0) were evaluated and the relative expression of each miRNA between control and patient were examined, 472 of which were detected in control or patient samples. Only 77 miRNAs were up-regulated and 67 miRNAs were down-regulated in the patient group when compared to the control group with fold changes >2.0 and a *P*-value of <0.001 **(Table S3)**. The top 40 miRNAs differentially expressed in patient samples with counts>200 and fold changes>2.0 and *P*-value of <0.001 are listed in **Table S4**. Of these, miR-9*, miR-9, miR-181a and miR-128 exhibited a significantly high abundance, whilst miR-582-5p, miR-223, miR-143, miR-126 etc. displayed the most significantly reduction in the patient group **(** **Fig. 3** **)**. We further performed qRT-PCR to examine the expression levels of randomly selected miR-223. Results showed that expression of miR-223 is significantly reduced over 2 fold compared to control in 85% (17/20) of ALL patients (part of data is shown in **Fig. 4A**). Additionally, we have found that some data in this study are consistent with previous findings reported by Mi et al [26] using microarray analysis. For example, miR-128, miR-130b and miR-210 were up-regulated and miR-424, miR-223, miR-23a, miR-27a were down-regulated in the patient group, indicating that there is a concordance between the findings despite using completely different miRNA expression analysis platforms, which suggests that these novel techniques are robust.

To investigate the potential disease relevance of novel miRNAs, we compared different expression of novel miRNAs in the patient group with normal control. Only 20 out of 201 newly identified miRNAs/miRNA*s exhibited significantly differential expression between the two groups with fold changes >2.0 and a *P*-value of <0.001. Among them, 6 miRNAs exhibited increased expression and 14 other miRNAs showed reduced expression **(** **Table 2** **)**. The six most highly expressed miRNAs are miR-1943, miR-1841, miR-1931, miR-1987, miR-1890, miR-1902 and the most lowly expressed miRNAs are miR-1893, miR-1971*, miR-1834, miR-1842*, miR-1842. We further used qRT-PCR to examine expression levels of three randomly selected highly down-regulated novel miRNA candidates: miR-1842, miR-1859* and miR-1852 in 20 ALL patients' and three normal donors' samples. Results showed that all these miRNAs can be detected in all clinical samples. Interestingly, we found that miR-1842 (16/20), miR-1852 (13/20) and miR-1859* (14/20) had significantly reduced expressions in most ALL patients compared to normal donors. This is consistent with our results derived using Solexa sequencing technology and suggests that these novel miRNAs may have functional relevance in the pathogenesis of ALL (part of data is shown in **Fig. 4B–D**).

**Table 2:** The top 6 up-regulated and 14 down-regulated novel miRNAs differentially expressed in patient group (fold changes>2.0 and *P*-value of <0.001).

MicroRNA	Control reads	Patient reads	Control percent	Patient percent	Fold changes	P-value
hsa-miR-1943	0	31	0	100		8.15E-17
hsa-miR-1841	0	5	0	100		0.000962473
hsa-miR-1931	0	5	0	100		0.000962473
hsa-miR-1987	0	5	0	100		0.000962473
hsa-miR-1890	8	54	6.392458766	93.60754123	14.64343294	4.51E-20
hsa-miR-1902	3	14	8.989689382	91.01031062	10.12385487	8.25E-06
hsa-miR-1859	103	14	77.22785611	22.77214389	0.294869559	6.11E-07
hsa-miR-1859*	121	14	79.935753	20.064247	0.251004666	5.31E-09
hsa-miR-1947	120	12	82.17333704	17.82666296	0.216939747	6.78E-10
hsa-miR-1971	125	11	83.96961466	16.03038534	0.190906978	4.38E-11
hsa-miR-1866	30	2	87.36474314	12.63525686	0.144626498	0.000498687
hsa-miR-1986	33	2	88.37992777	11.62007223	0.131478635	0.000189704
hsa-miR-1843	43	2	90.83458937	9.16541063	0.100902208	6.98E-06
hsa-miR-1852	62	2	93.45964161	6.540358394	0.069980564	1.05E-08
hsa-miR-1852*	40	1	94.85551703	5.144482971	0.054234937	2.67E-06
hsa-miR-1842	355	4	97.61393329	2.386066705	0.024443915	3.31E-52
hsa-miR-1842*	44	0	100	0	0	4.25E-08
hsa-miR-1834	37	0	100	0	0	5.96E-07
hsa-miR-1971*	34	0	100	0	0	1.85E-06
hsa-miR-1893	18	0	100	0	0	0.000771849

### Gene Ontology analysis reveals a set of abnormally expressed miRNAs involved in the pathway of nervous system development

**Figure 5:**
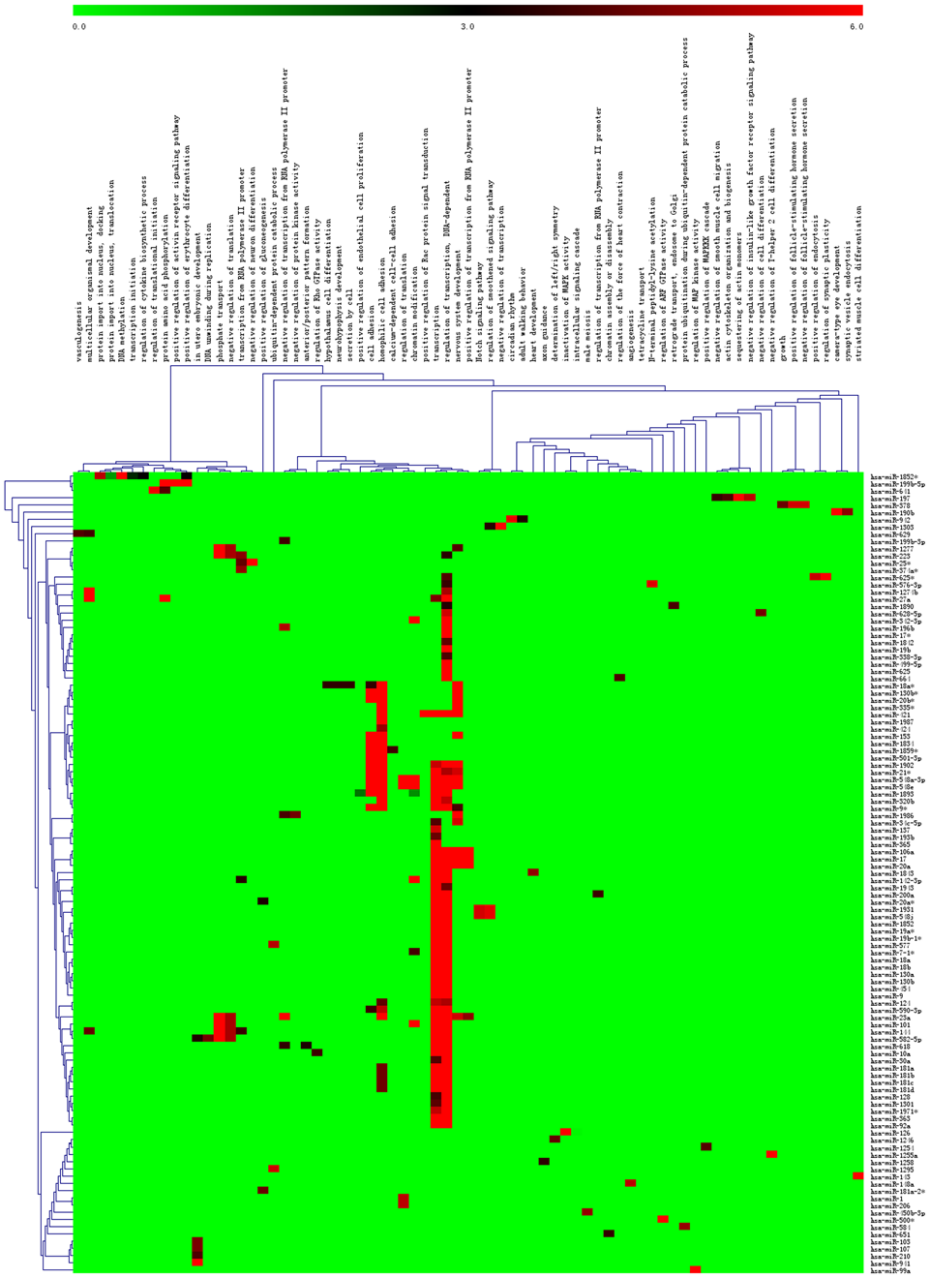
Clustering of over-represented Gene Ontology (GO) classes in predicted targets of differential microRNAs (fold changes>2.0 and P-value of <0.001). All genes with statistically over-represented GO annotations were included (P<0.001).

The targets of the 171 known and novel miRNAs displaying significantly different expression between the normal donors and patient group (fold changes >2.0 and a *p*-value of <0.001, **Table S5**) [35]–[36] were predicted by miRNAs TargetScan to further elucidate their pathological relevance. By examining the significant GO “biological process” classifications that are over-represented among these likely targets genes of the differentially expressed miRNAs, we analyzed the functional annotation for predicted target sets expected to shed light on the specific function of miRNAs significant to ALL biology. Clustering of over-represented GO classes in predicted targets of up-regulated and down-regualted miRNAs in ALL showed that the most significant GO terms were genes involved in cell adhesion, hemophilic cell adhesion, transcription and regulation of transcription (**Fig. 5**). Interestingly, another cluster of highly significant GO terms was associated with nervous system development, i.e. neuronal cell differentiation, which might be associated with the pathogenesis of ALL with subsequent central nervous system (CNS) relapse, suggesting that a set of significantly dysregulated miRNAs might promote the progression of CNS relapse in ALL by regulating genes in the pathway of nervous system development.

## Discussion

To gain insight into the roles of miRNAs in the progression of ALL, we have employed SBS strategy to globally study small RNAs, especially miRNA expression profiles in ALL patients and their normal counterparts. Our results revealed that there are a large number of miRNAs deregulated in ALL patients, of which some known and novel miRNAs may be involved in the progression of ALL or relapse.

Although 678 human miRNA sequences have been cataloged (miRBase, release 11.0, 2008) and identified by either cloning or computational prediction, the identification of unannotated and cancer-specific novel miRNAs candidates which are of low abundance or nonconserved is important to comprehensively understand the pathogenesis of leukemia and explore new therapeutic strategies. In this study, we identified 275 novel miRNAs/miRNA*s. Amongst these were 20 newly identified miRNAs, including 6 up-regulated and 14 down-regulated miRNAs exhibiting significantly differential expression in the patient group with fold changes >2.0 and a *p*-value of <0.001. We further used qRT-PCR to validate the expression of miR-1842 and miR-1852 and the results revealed that 80% (16/20) and 65% (13/20) of ALL patients had significantly down-regulated miR-1842 and miR-1852, respectively. This indicates that these novel miRNAs may have functional relevance in the pathogenesis of ALL. Schotte et al. [24] identified 8 new miRNA genes from childhood ALL patients using a cloning strategy. However, these 8 new miRNA genes were filtered out in our study for they mostly overlapped with tRNA, rRNA, AluSx, HY3 RNA, HY5 RNA,or due to poor secondary structure.

MiRNA*, also called passenger strand, has been considered as an inactive strand and prone to be degradation [37]. However, recent studies found that both pre-miRNA arms can functionally suppress the expression of their target genes and the miRNA* strand has inhibitory activity [30], [38]–[39]. By analyzing the precursor sequence of miRNAs, we identified a total of 116 novel miRNA*s, including 74 miRNA*s of known miRNAs and 42 miRNA*s of novel miRNAs and found that some miRNA*s were significantly dysregulated in ALL patient group with fold changes >2.0 and a *p*-value of <0.001. For example, we elucidated that miR-1859* was significantly down-regulated about 4 fold in 70% (14/20) patients, suggesting miRNA*s could be involved in the progression of ALL. Interestingly, *BCL-2*, an anti-apoptotic gene, was predicted to be one of the conserved targets of miR-1859* based on the targetscan prediction algorithm (www.targetscan.com) for the seed region (TTGTCCT). Overexpression of the BCL-2 protein has been frequently observed in many cancers including ALL [40]–[41]. Recently, it has been demonstrated that ALL cells are dependent on BCL-2, suggesting BCL-2 might be a more clinically significant parameter in ALL [42]. Therefore it is likely that down-regulated miRNA-1859* contributes to the progression of ALL by targeting the anti-apoptotic gene *BCL-2*.

In order to highlight the genes that are likely to be the targets of the known and novel miRNAs and to analyse their relevance to the pathogenesis of ALL, we also examined the significant GO “biological process” classifications that are over-represented among these targets genes of the 171 known and novel differentially expressed miRNAs. This analysis revealed that the most significant GO terms were genes involved in cell adhesion, hemophilic cell adhesion, transcription and regulation of transcriptio. Interestingly, target prediction of the miRNA pattern revealed that some abnormally expressed miRNAs may be involved in the pathway of nervous system development, i.e. neuronal cell differentiation. Notably, we found that the nervous system development related gene *Sox11* was the target of miR-1986. *Sox11* is important for neurogenesis, neural cell survival and neurite outgrowth [43]–[44], which implies that miRNAs might be associated with the pathogenesis of ALL with subsequent central nervous system (CNS) relapse. CNS relapse is the main source of extramedullary relapse in ALL, and has accounted for 30–40% of initial relapses in some clinical trials [18], [41]. Further study of the differential expression patterns of known and novel miRNAs, together with the clinic follow up information, may reveal a miRNA signature as a biomarker for detection of early CNS relapse in pediatric ALL patients.

In conclusion, we present a comprehensive picture of the expression of small RNAs and in total have identified 159 novel miRNAs and 116 novel miRNA*s using an SBS strategy and computational analysis. To our knowledge, this is the first study to look at known and novel miRNA expression pattern in human ALL. Notably, we confirmed the expressions of some novel miRNAs and demonstrated that miRNAs are different between ALL patients and normal donors. Our data suggests these deregulated known and novel miRNAs may be associated with ALL or the onset of CNS relapses due to ALL.

## Methods

### Clinical samples and Ethics Statement

A total of 32 bone marrow samples including 27 ALL untreated pediatric patients and 5 normal donors were enrolled from the First and Second Affiliated Hospital of Sun Yat-sen University in this study. Among them, 3 ALL patients' and 2 normal donors' (control group) bone marrows were pooled respectively for small RNA library construction for Solexa sequencing. Another 24 ALL patients and 3 normal donors were used as a validation set to confirm the miRNA differential expression by qRT-PCR. Bone marrow samples were taken by bone marrow puncture at diagnosis. Written informed consent for biological studies was obtained from all patients analyzed. The study was approved by the Ethics Committee of the affiliated hospitals of Sun Yat-sen University.

### Total RNA isolation, small RNA library preparation, and nucleotide sequencing

Total RNA was isolated with Trizol (Invitrogen, Carlsbad, CA) according to the instructions of the manufacturer. Small RNA library preparation and sequencing were performed with Solexa sequencing Technology (BGI, Shenzhen, China). Briefly, total RNA was size fractionated on a 15% tris-borate-EDTA (TBE) urea polyacrylamide gel and a 15–30 nt fraction was excised, using 10 bp ladder (Invitrogen) as marker. RNA was eluted from the polyacrylamide gel slice by Spin-X filter (Fisher) and precipitated by addition of 750 µL of 100% ethanol and 3 µL of glycogen and incubating at −80°C for 30 minutes. After washing with 75% ethanol, the RNA pellet was dissolved in DEPC-treated water. The RNA was dephosphorylated by alkaline phosphatase and recovered by ethanol precipitation. The small RNAs pools were then ligated sequentially to a 5′RNA adapter (5′-GUUCAGAGUUCUACAGUCCGACGAUC-3′) with T4 RNA ligase (Promega). The ligated RNA was size fractionated on a 15% TBE urea polyacrylamide gel, and a 40–60 nt fraction was excised. The RNA was eluted, precipitated and resuspended as described above. The 3′RNA adapter (5′-pUCGUAUGCCGUCUUCUGCUUGidT-3′; p, phosphate; idT, inverted deoxythymidine) was subsequently ligated to the precipitated RNA with T4 RNA ligase. Ligated RNA was size fractionated on a 10% TBE urea polyacrylamide gel, and the 70–90 nt fraction was excised. The RNA was eluted from the polyacrylamide gel and precipitated and resuspended in DEPC water. Then, reverse transcription reaction was preformed after ligation with adapters using Superscript II reverse transcriptase (Invitrogen) and Solexa's small RNA RT-Primer (5′-CAAGCAGAAGACGGCATACGA-3′) and followed by PCR amplification with Hotstart Phusion DNA Polymerase (NEB) in 15 cycles using Solexa's small RNA primer set (5′-CAAGCAGAAGACGGCATACGA-3′; 5′-AATGATACGGCGACCACCGACAGGTTCAGAGTTCTACAGTCCGA-3′). PCR products were purified on a 6% TBE polyacrylamide gel using a 25 bp DNA marker (Invitrogen). An approximately 92 bp fraction was excised, eluted and precipitated by ethanol. The pellet was dissolved in EB buffer and diluted for sequencing on the Solexa Genome Sequencer.

### Initial processing of reads

Low quality reads were filtered and 3′ adaptor sequence trimmed. Adaptor contaminants were cleaned up and 18–30 nt reads collected. The remaining reads were discarded. We calculated sequencing frequency of each unique small RNA sequence, the number of reads for each sequence reflecting relative abundance. Each unique sequence was aligned to the human reference genome (Version hg18, Mar. 2006, NCBI Build 36.1, obtained from the UCSC Genome Browser download page) using MegaBLAST [28] with the following options: -W 15 –F F. Only perfect matches over their entire length were set aside. All mapped sequences were also searched against the human miRNA, tRNA, rRNA, snoRNA, snRNA, scRNA or refGene (exonic) (miRBase 11.0, UCSC annotation). These annotated RNAs were removed. To avoid repeat associated sequences, reads with more than five total matching positions in the genome were discarded. We aligned known mature miRNA sequence to the human genome and 97% known mature sequences are less than five positions (**Fig. S2**). The genomic loci corresponding to sequences remaining after these filtering steps were then analyzed for the hairpin secondary structure that is characteristic of miRNA precursors. Each miRNA tally from each library was normalized to the total number of miRNA hairpin-matching reads for that library, and those normalized tallies were used for relative expression analysis.

### Known miRNAs expression analysis

Relative expression analysis was sought to determine the expression preferences of individual miRNAs between these two libraries. The number of reads matching a particular mature miRNA was calculated, but only sequence matches that overlapped at least 10 nt with the dominantly abundant mature miRNA sequence contributed to the miRNA tally. The Audic and Claverie test was used to establish the statistical significance of the difference in read frequencies for predicting differential miRNA expression based on the comparison of tag counts generated from digital expression analyses [46]. The program winflat (http://www.igs.cnrs-mrs.fr/SpipInternet/IMG/tgz/winflat.tgz) was used to compute the probability of differential miRNA. A *p-*value of ≤0.001 was chosen as a threshold for determining significant differential miRNA expression.

### Novel miRNAs prediction

Sequences from 100 nucleotide (nt) upstream to 100 nt downstream of the remaining aligned reads were extracted from the human genome. Potential miRNA stem loops were located by sliding a 100 nt window advancing by 10-nt increments along the strand of read sequences and folding the window with the secondary structure prediction program RNAfold[47] to identify stem-loop structures with a folding free energy of at most −18 kcal/mole (Mfe≤−18 kcal/mole). For overlapped hairpins, we only took the one that had the greatest number of paired arm bases. Forked hairpins were permitted provided that the longest forked segment contained no more than eight base pairs. For forked hairpins, we folded the sequence again with the program RNAshapes [48] and discarded the forked hairpins without stem-loop optional structure. Only structures that: 1) folded into hairpins, 2) contained a read in one of the hairpin arm, 3) loop length of hairpin is less than 20 and minimum pairs of arm is 15, 4) more than 2 reads located within hairpin, 5) the read sequences does not span the loop and 6) potential precursors are consistent with miRNA biogenesis [14], [45], were used in further analysis. We also used a recently discovered property of miRNAs to have lower folding free energies than random sequences with the same nucleotide content [31]–[32]. We thus use z-scores as described by Washietl et al [33] to filter the candidates. Only hairpins with the z-score less than −3.0 were novel miRNA genes. Furthermore, the observation of both a candidate miRNA and a corresponding candidate miRNA* was defined as high stringency. The sequence with dominantly abundant reads was named as mature miRNA and its corresponding complementary sequence as miRNA*.

### Target prediction and Gene Ontology (GO) analysis

The conserved targets of known miRNAs and novel miRNAs were predicted by TargetScan (http://www.targetscan.org/vert_42/vert_42_data_download/targetscan_41.zip) [35]–[36]. Only miRNAs with significant over-expression in either C group or P group were included in target analyses. GO [49] terms and gene information were downloaded from the NCBI website (ftp://ftp.ncbi.nih.gov/gene/DATA) on August 2008. The functional categories scrutinized are biological processes and molecular functions as defined in the Gene Ontology Consortium database (http://www.geneontology.org)[49]. Significant overrepresentation of particular GO terms in the dataset was determined using the software GeneMerge with corrections for multiple tests [50]. Bonferroni-corrected *p*-values are reported and a cutoff of 0.1 on the Bonferroni-corrected P value was applied. As described by Lall et al [51], Bonferroni-corrected P values for overrepresented GO terms for the targets of each miRNA are plotted on a negative log2 scale (e.g. *p*-values 2^0^ = 1, 2^−6^ = 0.015625). We performed 2-way hierarchical clustering with the program MeV [52], with the Pearson correlation coefficient and average linkage clustering.

### Quantitative real-time reverse transcription PCR assays

Quantitative real-time RT-PCR (qRT-PCR) was performed as described[53] and employed a Hairpin-it^TM^ miRNAs Real-Time PCR Quantitation Kit containing stem-loop like RT primer, miRNA specific PCR primers and Molecular Beacon probe (GenePharma, Shanghai, China). Briefly, RNA was reverse-transcribed to cDNA with miRNA specific stem-loop like RT primer and the expression of each miRNA relative to normal control was determined using the 2^-ΔΔC^_T_ method[54]. Comparative real-time PCR was performed in triplicate. The no-template real-time PCR was used as negative control in each PCR reaction.

## Supporting Information

Table S1 (0.05 MB DOC)

Table S2 (0.07 MB DOC)

Table S3 (0.33 MB DOC)

Table S4 (0.10 MB DOC)

Table S5 (0.22 MB DOC)

Figure S1 Alignment of 159 novel miRNAs in C group and P group(q means the read comes from C group; t means the read comes from P group)
(0.29 MB DOC)

Figure S2 The loci number of known miRNAs
(0.15 MB TIF)
