# Supplementary material for: Genome-Wide Analysis of Small RNA and Novel MicroRNA Discovery in Human Acute Lymphoblastic Leukemia Based on Extensive Sequencing Approach
Source: PLoS One. 2009 Sep 2;4(9):e6849. doi: 10.1371/journal.pone.0006849 (PMC2731166; doi:10.1371/journal.pone.0006849)
Supplement: Table S1 — (0.05 MB DOC) [file pone.0006849.s001.doc]

Table S1. Distribution of sequenced small RNAs across different categories in control group and patient group

| Category | Control group (C) | | | Patient group (P) | | |
| --- | --- | --- | --- | --- | --- | --- |
|  | genome loci | unique reads | total reads | genome loci | unique reads | total reads |
|  |  | 113,414 | 1,993,962 |  | 60,460 | 831,728 |
| Matching hg18 | 3,905,630 | 23,859 | 1,327,742 | 4,119,366 | 15,537 | 608,358 |
| Known miRNAs |  | 3,276 | 1,214,819 |  | 2,513 | 555,758 |
| Known snoRNAs |  | 1,157 | 11,715 |  | 994 | 7,846 |
| Known tRNAs |  | 2,575 | 28,030 |  | 1,479 | 6,726 |
| Known rRNAs |  | 578 | 1,323 |  | 735 | 984 |
| Known snRNAs |  | 249 | 582 |  | 263 | 1,011 |
| Known scRNAs |  | 647 | 9,577 |  | 542 | 3,824 |
| RefSeq |  | 1,579 | 1,822 |  | 990 | 1,034 |
| Repeats(>5 copies) |  | 1000 | 4746 |  | 665 | 1576 |
| Unannotated |  | 12798 | 55128 |  | 7356 | 29599 |

snoRNA, small nucleolar RNA; tRNA, transferRNA; rRNA, ribosomal RNA; snRNA, small nuclear RNA; scRNA, small cytoplasmic RNA.
