# Supplementary material for: Genome-Wide Analysis of Small RNA and Novel MicroRNA Discovery in Human Acute Lymphoblastic Leukemia Based on Extensive Sequencing Approach
Source: PLoS One. 2009 Sep 2;4(9):e6849. doi: 10.1371/journal.pone.0006849 (PMC2731166; doi:10.1371/journal.pone.0006849)
Supplement: Table S2 — (0.07 MB DOC) [file pone.0006849.s002.doc]

**Table S2**. 74 known miRNA stars.

| miRNA | sequence |
| --- | --- |
| hsa-mir-98* | CTATACAACTTACTACTTTCC |
| hsa-mir-103-2* | AGCTTCTTTACAGTGCTGCCTTGT |
| hsa-mir-101-2* | CGGTTATCATGGTACCGATGCT |
| hsa-mir-107* | AGCTTCTTTACAGTGTTGCCTTGT |
| hsa-mir-128-2* | GGGGGCCGATACACTGTACGAG |
| hsa-mir-147b-5p | TGGAAACATTTCTGCACAAACT |
| hsa-mir-181b-1* | CTCACTGAACAATGAATGCAA |
| hsa-mir-181b-2* | ACTCACTGATCAATGAATGCA |
| hsa-mir-196b* | TCGACAGCACGACACTGCCTTCA |
| hsa-mir-197* | CGGGTAGAGAGGGCAGTGGGAGG |
| hsa-mir-301a-5p | GCTCTGACTTTATTGCACTACT |
| hsa-mir-345* | GCCCTGAACGAGGGGTCTGGAG |
| hsa-mir-376b-5p | GTGGATATTCCTTCTATGTTTA |
| hsa-mir-421* | CTCATTAAATGTTTGTTGAATGA |
| hsa-mir-450a-1* | ATTGGGAACATTTTGCATGTAT |
| hsa-mir-450a-2* | TTGGGGACATTTTGCATTCAT |
| hsa-mir-451* | TTTAGTAATGGTAATGGTTCT |
| hsa-mir-503* | GGGGTATTGTTTCCGCTGCCAGG |
| hsa-mir-511-1-3p | AATGTGTAGCAAAAGACAGAA |
| hsa-mir-539-3p | ATACAAGGACAATTTCTTTTT |
| hsa-mir-548e* | CAAAAGCAATCGCGGTTTTTG |
| hsa-mir-548g-5p | CAAAAGTAATTGCAGTTTTT |
| hsa-mir-548h-2-3p | ACCACAATTACTTTTGCACC |
| hsa-mir-548h-3-3p | CAAAAACTGCAATTACTTTTG |
| hsa-mir-548h-4-3p | CAAAAACCGCAATTACTTTTG |
| hsa-mir-548j* | CAAAAACTGCATTACTTTTG |
| hsa-mir-548k-3p | AAAAACCGCAATTATTTTTGCT |
| hsa-mir-548l-3p | TGGCAAAAACTGCAGTTACTT |
| hsa-mir-559-3p | TTTGGTGCATATTTACTTTAGG |
| hsa-mir-561-5p | ATCAAGGATCTTAAACTTTG |
| hsa-mir-570-5p | AAAGGTAATTGCAGTTTTTCCCA |
| hsa-mir-577-3p | GGTTTCAATACTTTATCTGCTCT |
| hsa-mir-579-5p | TCGCGGTTTGTGCCAGATGACG |
| hsa-mir-580-5p | TAATGATTCATCAGACTCAGAT |
| hsa-mir-599-5p | TTTGATAAGCTGACATGGGACA |
| hsa-mir-600-5p | CATAGGAAGGCTCTTGTCTGTC |
| hsa-mir-627-3p | TCTTTTCTTTGAGACTCACT |
| hsa-mir-651-3p | AAAGGAAAGTGTATCCTA |
| hsa-mir-652-5p | ACAACCCTAGGAGAGGGTGC |
| hsa-mir-653-3p | ACTGGAGTTTGTTTCAATA |
| hsa-mir-659-5p | AGGACCTTCCCTGAACCAAGGA |
| hsa-mir-660* | ACCTCCTGTGTGCATGGATTA |
| hsa-mir-766-5p | AGGAGGAATTGGTGCTGGTCTTT |
| hsa-mir-873-3p | GGAGACTGATGAGTTCCCGGGA |
| hsa-mir-874-5p | CGGCCCCACGCACCAGGGTAAGA |
| hsa-mir-1247-3p | CGGGAACGTCGAGACTGGAGC |
| hsa-mir-1249-5p | AGGAGGGAGGAGATGGGCCAAGTT |
| hsa-mir-1255a-3p | CTATCTTCTTTGCTCATCCTTG |
| hsa-mir-1259-3p | AGCCAGTTTCTGTCTGATA |
| hsa-mir-1270-3p | AGGCTTTTCTTTATCTTCTATG |
| hsa-mir-1271-3p | AGTGCCTGCTATGTGCCAGGCA |
| hsa-mir-1277-5p | TATATATATATATGTACGTATG |
| hsa-mir-1278-5p | ATGATATGCATAGTACTCCC |
| hsa-mir-1284-3p | AAAGCCCATGTTTGTATTGGAA |
| hsa-mir-1292-3p | TCGCGCCCCGGCTCCCGTTCCA |
| hsa-mir-1302-3-5p | TAGCATAAATATTTCCCAAGCTT |
| hsa-mir-1303-5p | AGCGAGACCTCAACTCTACAATT |
| hsa-mir-1306-5p | CCACCTCCCCTGCAAACGTCCA |
| hsa-mir-1307-5p | TCGACCGGACCTCGACCGGCT |
| hsa-mir-1826-3p | GCCTGTCTGAGCGTCGCT |
| hsa-mir-212-5p | ACCTTGGCTCTAGACTGCTTA |
| hsa-mir-365-1-5p | AGGGACTTTTGGGGGCAGATGTG |
| hsa-mir-365-2-5p | AGGGACTTTCAGGGGCAGCTGTG |
| hsa-mir-382-3p | AATCATTCACGGACAACACT |
| hsa-mir-548f-4-5p | AAAAGTAATAGTGGTTTTTGC |
| hsa-mir-548p-5p | AAAATTAATTGCAGTTTTT |
| hsa-mir-597-3p | AGTGGTTCTCTTGTGGCTCAAG |
| hsa-mir-619-5p | TGCTGGGATTACAGGCATGAG |
| hsa-mir-642-3p | AGACACATTTGGAGAGGG |
| hsa-mir-643-5p | ACCTGAGCTAGAATACAAGTAG |
| hsa-mir-942-3p | CACATGGCCGAAACAGAGAAGT |
| hsa-mir-1255b-2-3p | CACTTTCTTTGCTCATCCAT |
| hsa-mir-1256-3p | CTAAAGAGAAGTCAATGCATGA |
| hsa-mir-1258-5p | CCACGACCTAATCCTAACTCCTG |
