# Supplementary material for: Genome-Wide Analysis of Small RNA and Novel MicroRNA Discovery in Human Acute Lymphoblastic Leukemia Based on Extensive Sequencing Approach
Source: PLoS One. 2009 Sep 2;4(9):e6849. doi: 10.1371/journal.pone.0006849 (PMC2731166; doi:10.1371/journal.pone.0006849)
Supplement: Table S3 — (0.33 MB DOC) [file pone.0006849.s003.doc]

**Table S3. The top 144 known miRNAs differentially expressed in patient samples with fold changes>2.0 and P-value of <0.001**

| **MicroRNA** | **Control reads** | **Patient reads** | **Control percent** | **Patient percent** | **Fold changes** | **P-value** |
| --- | --- | --- | --- | --- | --- | --- |
| hsa-miR-9 | 60 | 2072 | 1.317236249 | 98.68276375 | 74.91652603 | 0 |
| hsa-miR-181a | 3500 | 12324 | 11.57573753 | 88.42426247 | 7.638758413 | 0 |
| hsa-miR-128 | 1820 | 5995 | 12.27611449 | 87.72388551 | 7.145899914 | 0 |
| hsa-miR-181b | 1153 | 3043 | 14.86883091 | 85.13116909 | 5.725478324 | 0 |
| hsa-miR-363 | 1080 | 2738 | 15.38503118 | 84.61496882 | 5.499824332 | 0 |
| hsa-miR-342-3p | 1110 | 2076 | 19.77316386 | 80.22683614 | 4.057359596 | 0 |
| hsa-miR-92a | 10300 | 15451 | 23.50559187 | 76.49440813 | 3.254306829 | 0 |
| hsa-miR-107 | 7581 | 10152 | 25.60740043 | 74.39259957 | 2.90512111 | 0 |
| hsa-miR-103 | 35550 | 33204 | 33.04435444 | 66.95564556 | 2.026235546 | 0 |
| hsa-miR-101 | 18304 | 2551 | 76.78453693 | 23.21546307 | 0.30234555 | 0 |
| hsa-miR-23a | 6732 | 833 | 78.83727347 | 21.16272653 | 0.268435546 | 0 |
| hsa-miR-142-3p | 5768 | 635 | 80.72137147 | 19.27862853 | 0.238829299 | 0 |
| hsa-miR-27a | 5144 | 539 | 81.47871529 | 18.52128471 | 0.227314393 | 0 |
| hsa-miR-451 | 73984 | 5940 | 85.16612386 | 14.83387614 | 0.174175781 | 0 |
| hsa-miR-199b-3p | 10328 | 603 | 88.7579049 | 11.2420951 | 0.126660213 | 0 |
| hsa-miR-144* | 4316 | 134 | 93.689641 | 6.310358996 | 0.06735386 | 0 |
| hsa-miR-143 | 6537 | 128 | 95.92522673 | 4.074773272 | 0.042478641 | 0 |
| hsa-miR-223 | 112309 | 1912 | 96.43826309 | 3.561736913 | 0.036932819 | 0 |
| hsa-miR-144 | 2811 | 125 | 91.20183923 | 8.798160773 | 0.096469116 | 4.01E-301 |
| hsa-let-7d | 5362 | 5112 | 32.59186164 | 67.40813836 | 2.06825063 | 1.42E-299 |
| hsa-miR-192 | 3308 | 3534 | 30.14218949 | 69.85781051 | 2.317609028 | 2.27E-263 |
| hsa-miR-181a* | 216 | 921 | 9.756031148 | 90.24396885 | 9.250069776 | 5.73E-261 |
| hsa-miR-130b | 447 | 1157 | 15.11670873 | 84.88329127 | 5.615196588 | 2.12E-245 |
| hsa-miR-378 | 2762 | 3062 | 29.36831001 | 70.63168999 | 2.405030796 | 8.65E-245 |
| hsa-miR-424 | 2793 | 227 | 85.01110221 | 14.98889779 | 0.176316945 | 4.57E-224 |
| hsa-miR-30e* | 3195 | 349 | 80.84269274 | 19.15730726 | 0.236970178 | 4.72E-206 |
| hsa-miR-148a | 2983 | 305 | 81.845629 | 18.154371 | 0.221812346 | 2.73E-203 |
| hsa-miR-122 | 35 | 472 | 3.305143273 | 96.69485673 | 29.25587448 | 4.39E-190 |
| hsa-miR-223* | 3775 | 580 | 75.00123379 | 24.99876621 | 0.3333114 | 2.92E-170 |
| hsa-miR-486-5p | 2038 | 176 | 84.22135081 | 15.77864919 | 0.187347377 | 9.40E-158 |
| hsa-miR-9* | 2 | 305 | 0.301356301 | 99.6986437 | 330.8331145 | 3.17E-150 |
| hsa-miR-335 | 1395 | 65 | 90.81967181 | 9.180328185 | 0.101083036 | 6.92E-148 |
| hsa-miR-92a-1* | 37 | 377 | 4.328179676 | 95.67182032 | 22.10440127 | 8.37E-144 |
| hsa-miR-199b-5p | 1062 | 21 | 95.88668279 | 4.113317205 | 0.04289769 | 1.86E-141 |
| hsa-miR-20a | 1530 | 1710 | 29.20031403 | 70.79968597 | 2.424620704 | 8.82E-140 |
| hsa-miR-374a* | 1592 | 132 | 84.75476505 | 15.24523495 | 0.179874665 | 5.48E-127 |
| hsa-miR-17 | 1663 | 1728 | 30.72960831 | 69.27039169 | 2.254190518 | 3.12E-123 |
| hsa-miR-19b | 2132 | 296 | 76.85260047 | 23.14739953 | 0.301192144 | 1.90E-109 |
| hsa-miR-542-3p | 1384 | 142 | 81.79405854 | 18.20594146 | 0.222582689 | 3.71E-95 |
| hsa-miR-130a | 695 | 894 | 26.38130481 | 73.61869519 | 2.790563079 | 6.71E-93 |
| hsa-miR-181d | 513 | 709 | 25.01093549 | 74.98906451 | 2.998251087 | 5.95E-82 |
| hsa-miR-499-5p | 234 | 465 | 18.82890905 | 81.17109095 | 4.310982156 | 5.00E-81 |
| hsa-miR-30a | 943 | 76 | 85.11796676 | 14.88203324 | 0.174840093 | 2.32E-77 |
| hsa-miR-17* | 289 | 499 | 21.07136843 | 78.92863157 | 3.745776258 | 9.66E-76 |
| hsa-miR-598 | 149 | 359 | 16.05925858 | 83.94074142 | 5.226937533 | 8.33E-74 |
| hsa-miR-181c | 150 | 336 | 17.06644723 | 82.93355277 | 4.859450337 | 1.94E-65 |
| hsa-miR-1295 | 0 | 126 | 0 | 100 | #DIV/0! | 1.41E-64 |
| hsa-miR-193b* | 4 | 129 | 1.409183709 | 98.59081629 | 69.96306847 | 1.26E-59 |
| hsa-miR-582-5p | 405 | 5 | 97.39159169 | 2.608408306 | 0.026782685 | 9.31E-59 |
| hsa-let-7c | 1131 | 158 | 76.74223184 | 23.25776816 | 0.303063484 | 1.35E-58 |
| hsa-miR-181a-2* | 44 | 196 | 9.377626045 | 90.62237395 | 9.663679648 | 2.29E-58 |
| hsa-miR-10a | 454 | 15 | 93.31177339 | 6.688226607 | 0.071676128 | 6.64E-55 |
| hsa-miR-25* | 59 | 201 | 11.91801476 | 88.08198524 | 7.390659184 | 1.55E-52 |
| hsa-miR-425 | 1352 | 260 | 70.56207811 | 29.43792189 | 0.417191822 | 4.80E-45 |
| hsa-miR-124 | 0 | 85 | 0 | 100 | #DIV/0! | 5.78E-44 |
| hsa-miR-532-5p | 510 | 41 | 85.14967946 | 14.85032054 | 0.174402542 | 9.86E-43 |
| hsa-miR-548a-3p | 33 | 142 | 9.675874591 | 90.32412541 | 9.334983061 | 3.30E-42 |
| hsa-miR-1246 | 2 | 87 | 1.048561065 | 98.95143894 | 94.36879003 | 1.09E-41 |
| hsa-miR-450a | 1001 | 171 | 72.96094028 | 27.03905972 | 0.370596371 | 1.51E-40 |
| hsa-miR-338-3p | 561 | 58 | 81.68018875 | 18.31981125 | 0.224287083 | 1.90E-39 |
| hsa-miR-1277 | 242 | 2 | 98.23868792 | 1.761312076 | 0.017928905 | 4.69E-37 |
| hsa-let-7e | 100 | 202 | 18.57982198 | 81.42017802 | 4.382182893 | 6.45E-37 |
| hsa-miR-143* | 342 | 22 | 87.75379286 | 12.24620714 | 0.139551884 | 7.16E-33 |
| hsa-miR-145* | 206 | 1 | 98.95786917 | 1.042130831 | 0.010531056 | 8.13E-33 |
| hsa-miR-130b* | 66 | 155 | 16.40743711 | 83.59256289 | 5.094797093 | 1.95E-32 |
| hsa-miR-618 | 230 | 7 | 93.80641807 | 6.193581928 | 0.06602514 | 3.66E-29 |
| hsa-miR-145 | 173 | 1 | 98.76154311 | 1.23845689 | 0.01253987 | 1.75E-27 |
| hsa-miR-196b | 179 | 2 | 97.63345508 | 2.366544923 | 0.024239078 | 5.43E-27 |
| hsa-miR-342-5p | 95 | 166 | 20.87361665 | 79.12638335 | 3.790736635 | 6.79E-27 |
| hsa-miR-126 | 245 | 12 | 90.39497338 | 9.605026616 | 0.106256203 | 7.29E-27 |
| hsa-miR-99a | 181 | 4 | 95.42508427 | 4.574915726 | 0.047942486 | 7.58E-25 |
| hsa-miR-625* | 4 | 56 | 3.187600021 | 96.81239998 | 30.37156461 | 2.62E-24 |
| hsa-miR-193b | 2 | 51 | 1.775579672 | 98.22442033 | 55.31963553 | 4.92E-24 |
| hsa-miR-629 | 37 | 99 | 14.69593224 | 85.30406776 | 5.804604046 | 2.15E-23 |
| hsa-miR-206 | 1 | 46 | 0.992139441 | 99.00786056 | 99.79228371 | 7.79E-23 |
| hsa-miR-1274b | 479 | 78 | 73.89546305 | 26.10453695 | 0.353263054 | 1.18E-21 |
| hsa-miR-106a | 99 | 147 | 23.68979699 | 76.31020301 | 3.221226549 | 2.97E-20 |
| hsa-miR-18a | 222 | 240 | 29.89273383 | 70.10726617 | 2.345294564 | 3.00E-20 |
| hsa-miR-34c-5p | 126 | 1 | 98.30739824 | 1.692601756 | 0.01721744 | 6.42E-20 |
| hsa-miR-1271 | 9 | 52 | 7.38863671 | 92.61136329 | 12.5342965 | 1.66E-18 |
| hsa-miR-504 | 106 | 0 | 100 | 0 | 0 | 2.96E-18 |
| hsa-miR-1 | 133 | 164 | 27.21054001 | 72.78945999 | 2.675046507 | 1.20E-17 |
| hsa-miR-21* | 258 | 31 | 79.32327491 | 20.67672509 | 0.260664037 | 6.49E-17 |
| hsa-miR-320b | 127 | 156 | 27.28682937 | 72.71317063 | 2.664771698 | 8.69E-17 |
| hsa-miR-30a* | 103 | 1 | 97.93723505 | 2.062764953 | 0.021062111 | 3.10E-16 |
| hsa-miR-582-3p | 83 | 0 | 100 | 0 | 0 | 1.74E-14 |
| hsa-miR-24-2* | 144 | 10 | 86.90720362 | 13.09279638 | 0.150652602 | 3.18E-14 |
| hsa-miR-425* | 292 | 52 | 72.13284267 | 27.86715733 | 0.386331057 | 2.33E-12 |
| hsa-miR-941 | 103 | 5 | 90.47231059 | 9.527689411 | 0.105310557 | 3.66E-12 |
| hsa-miR-548e | 432 | 98 | 67.01820692 | 32.98179308 | 0.492131834 | 1.05E-11 |
| hsa-miR-590-3p | 181 | 24 | 77.66055871 | 22.33944129 | 0.287654913 | 2.50E-11 |
| hsa-miR-365 | 79 | 2 | 94.79378728 | 5.206212721 | 0.054921455 | 2.72E-11 |
| hsa-miR-651 | 152 | 17 | 80.47447879 | 19.52552121 | 0.24262998 | 3.26E-11 |
| hsa-miR-625 | 64 | 85 | 25.76502134 | 74.23497866 | 2.881231017 | 5.28E-11 |
| hsa-miR-181c* | 31 | 57 | 20.04453042 | 79.95546958 | 3.988892126 | 6.46E-11 |
| hsa-miR-197 | 113 | 9 | 85.26720277 | 14.73279723 | 0.172783869 | 9.97E-11 |
| hsa-miR-221* | 326 | 68 | 68.84623017 | 31.15376983 | 0.452512356 | 1.03E-10 |
| hsa-miR-576-3p | 134 | 14 | 81.52261635 | 18.47738365 | 0.226653467 | 1.46E-10 |
| hsa-miR-19a* | 12 | 36 | 13.31878493 | 86.68121507 | 6.508192416 | 2.91E-10 |
| hsa-miR-577 | 4 | 25 | 6.868729014 | 93.13127099 | 13.5587342 | 5.50E-10 |
| hsa-miR-106a* | 24 | 46 | 19.38731501 | 80.61268499 | 4.158011821 | 1.88E-09 |
| hsa-miR-190b | 87 | 6 | 86.98574753 | 13.01425247 | 0.149613619 | 3.51E-09 |
| hsa-miR-210 | 14 | 35 | 15.56785217 | 84.43214783 | 5.42349368 | 4.57E-09 |
| hsa-miR-1301 | 33 | 52 | 22.63239925 | 77.36760075 | 3.418444501 | 7.98E-09 |
| hsa-miR-95 | 44 | 60 | 25.26356671 | 74.73643329 | 2.95826928 | 1.56E-08 |
| hsa-miR-20b* | 18 | 37 | 18.31730957 | 81.68269043 | 4.459317026 | 2.37E-08 |
| hsa-miR-500* | 82 | 87 | 30.28763129 | 69.71236871 | 2.301677806 | 2.97E-08 |
| hsa-miR-7-1* | 114 | 107 | 32.93602762 | 67.06397238 | 2.036188855 | 6.69E-08 |
| hsa-miR-1303 | 1 | 15 | 2.981429089 | 97.01857091 | 32.54096208 | 1.08E-07 |
| hsa-miR-664 | 52 | 2 | 92.29874379 | 7.701256209 | 0.083438364 | 3.30E-07 |
| hsa-miR-20a* | 48 | 58 | 27.6139711 | 72.3860289 | 2.621355279 | 3.31E-07 |
| hsa-miR-652 | 112 | 16 | 76.34089395 | 23.65910605 | 0.309913925 | 4.22E-07 |
| hsa-miR-421 | 89 | 86 | 32.29690713 | 67.70309287 | 2.096271714 | 4.99E-07 |
| hsa-miR-1258 | 0 | 11 | 0 | 100 | #DIV/0! | 9.26E-07 |
| hsa-miR-886-3p | 63 | 5 | 85.31153707 | 14.68846293 | 0.172174403 | 1.35E-06 |
| hsa-miR-550* | 41 | 1 | 94.9746867 | 5.025313298 | 0.052912133 | 1.87E-06 |
| hsa-miR-30d* | 14 | 26 | 19.88513938 | 80.11486062 | 4.028881019 | 6.14E-06 |
| hsa-miR-450b-3p | 37 | 1 | 94.461499 | 5.538501003 | 0.058632364 | 7.71E-06 |
| hsa-miR-19b-1* | 0 | 9 | 0 | 100 | #DIV/0! | 9.38E-06 |
| hsa-miR-137 | 0 | 9 | 0 | 100 | #DIV/0! | 9.38E-06 |
| hsa-miR-18b | 34 | 41 | 27.65457566 | 72.34542434 | 2.616038128 | 1.45E-05 |
| hsa-miR-335* | 20 | 30 | 23.50675563 | 76.49324437 | 3.254096208 | 1.48E-05 |
| hsa-miR-501-3p | 16 | 26 | 22.0981246 | 77.9018754 | 3.525270892 | 2.26E-05 |
| hsa-miR-27b* | 14 | 24 | 21.19107805 | 78.80892195 | 3.718967095 | 2.72E-05 |
| hsa-miR-184 | 0 | 8 | 0 | 100 | #DIV/0! | 2.99E-05 |
| hsa-miR-454 | 110 | 21 | 70.71347139 | 29.28652861 | 0.414157699 | 3.72E-05 |
| hsa-miR-505* | 35 | 40 | 28.74131936 | 71.25868064 | 2.479311396 | 3.90E-05 |
| hsa-miR-548j | 60 | 57 | 32.66983514 | 67.33016486 | 2.060927598 | 4.49E-05 |
| hsa-miR-574-3p | 37 | 2 | 89.50430232 | 10.49569768 | 0.117264728 | 5.13E-05 |
| hsa-miR-641 | 28 | 34 | 27.51585467 | 72.48414533 | 2.634268359 | 6.30E-05 |
| hsa-miR-23b* | 4 | 13 | 12.42152425 | 87.57847575 | 7.050541784 | 7.02E-05 |
| hsa-miR-345 | 110 | 22 | 69.74086762 | 30.25913238 | 0.433879494 | 7.29E-05 |
| hsa-miR-573 | 1 | 9 | 4.872207539 | 95.12779246 | 19.52457725 | 7.37E-05 |
| hsa-miR-18a* | 1 | 9 | 4.872207539 | 95.12779246 | 19.52457725 | 7.37E-05 |
| hsa-miR-584 | 29 | 1 | 93.03997623 | 6.960023766 | 0.074806809 | 0.000126975 |
| hsa-miR-140-5p | 34 | 2 | 88.6830169 | 11.3169831 | 0.127611616 | 0.000137059 |
| hsa-miR-942 | 30 | 33 | 29.53043171 | 70.46956829 | 2.386337219 | 0.000255978 |
| hsa-miR-153 | 0 | 6 | 0 | 100 | #DIV/0! | 0.000302427 |
| hsa-miR-505 | 70 | 12 | 72.89178501 | 27.10821499 | 0.371896709 | 0.000321308 |
| hsa-miR-628-5p | 39 | 4 | 81.79943678 | 18.20056322 | 0.222502305 | 0.000463336 |
| hsa-miR-1255a | 43 | 5 | 79.85590672 | 20.14409328 | 0.25225552 | 0.000468936 |
| hsa-miR-574-5p | 30 | 2 | 87.36474314 | 12.63525686 | 0.144626498 | 0.000498687 |
| hsa-miR-1254 | 2 | 8 | 10.33315125 | 89.66684875 | 8.677589888 | 0.000846081 |
| hsa-miR-200a | 8 | 14 | 20.84877195 | 79.15122805 | 3.796445576 | 0.000882212 |
