# Supplementary material for: Genome-Wide Analysis of Small RNA and Novel MicroRNA Discovery in Human Acute Lymphoblastic Leukemia Based on Extensive Sequencing Approach
Source: PLoS One. 2009 Sep 2;4(9):e6849. doi: 10.1371/journal.pone.0006849 (PMC2731166; doi:10.1371/journal.pone.0006849)
Supplement: Table S4 — (0.10 MB DOC) [file pone.0006849.s004.doc]

**Table S4. The top 20 up-regulated and 20 down-regulated known miRNAs differentially expressed in patient group (counts>200 and fold changes>2.0 and *P*-value of <0.001)**

| **MicroRNA** | **Normal donor reads** | **Patient reads** | **Normal donor percent** | **Patient percent** | **Fold changes** | **P-value** |
| --- | --- | --- | --- | --- | --- | --- |
| hsa-miR-9* | 2 | 305 | 0.301534545 | 99.69846546 | 330.6369606 | 3.17E-150 |
| hsa-miR-9 | 60 | 2072 | 1.318007414 | 98.68199259 | 74.87210737 | 0 |
| hsa-miR-122 | 35 | 472 | 3.307039239 | 96.69296076 | 29.23852842 | 4.39E-190 |
| hsa-miR-92a-1* | 37 | 377 | 4.330636215 | 95.66936379 | 22.09129538 | 8.37E-144 |
| hsa-miR-181a-2* | 44 | 196 | 9.382667426 | 90.61733257 | 9.657949968 | 2.29E-58 |
| hsa-miR-181a* | 216 | 921 | 9.761254046 | 90.23874595 | 9.24458533 | 5.73E-261 |
| hsa-miR-181a | 3500 | 12324 | 11.58180959 | 88.41819041 | 7.634229327 | 0 |
| hsa-miR-25* | 59 | 201 | 11.92424214 | 88.07575786 | 7.386277198 | 1.55E-52 |
| hsa-miR-128 | 1820 | 5995 | 12.2825029 | 87.7174971 | 7.141663048 | 0 |
| hsa-miR-181b | 1153 | 3043 | 14.87633975 | 85.12366025 | 5.722083638 | 0 |
| hsa-miR-130b | 447 | 1157 | 15.1243205 | 84.8756795 | 5.61186729 | 2.12E-245 |
| hsa-miR-363 | 1080 | 2738 | 15.39275356 | 84.60724644 | 5.496563438 | 0 |
| hsa-miR-598 | 149 | 359 | 16.06725512 | 83.93274488 | 5.223838436 | 8.33E-74 |
| hsa-miR-181c | 150 | 336 | 17.07484328 | 82.92515672 | 4.856569127 | 1.94E-65 |
| hsa-let-7e | 100 | 202 | 18.58879567 | 81.41120433 | 4.379584659 | 6.45E-37 |
| hsa-miR-499-5p | 234 | 465 | 18.83797521 | 81.16202479 | 4.308426137 | 5.00E-81 |
| hsa-miR-342-3p | 1110 | 2076 | 19.78257388 | 80.21742612 | 4.054953952 | 0 |
| hsa-miR-17* | 289 | 499 | 21.08123391 | 78.91876609 | 3.743555355 | 9.66E-76 |
| hsa-miR-92a | 10300 | 15451 | 23.51625748 | 76.48374252 | 3.252377322 | 0 |
| hsa-miR-181d | 513 | 709 | 25.02206071 | 74.97793929 | 2.996473398 | 5.95E-82 |
| hsa-miR-486-5p | 2038 | 176 | 84.2292307 | 15.7707693 | 0.187236297 | 9.40E-158 |
| hsa-miR-374a* | 1592 | 132 | 84.76242675 | 15.23757325 | 0.179768016 | 5.48E-127 |
| hsa-miR-424 | 2793 | 227 | 85.01865785 | 14.98134215 | 0.176212405 | 4.57E-224 |
| hsa-miR-30a | 943 | 76 | 85.12547797 | 14.87452203 | 0.174736429 | 2.32E-77 |
| hsa-miR-532-5p | 510 | 41 | 85.15717745 | 14.84282255 | 0.174299137 | 9.86E-43 |
| hsa-miR-451 | 73984 | 5940 | 85.17361499 | 14.82638501 | 0.174072511 | 0 |
| hsa-miR-143* | 342 | 22 | 87.76016503 | 12.23983497 | 0.139469143 | 7.16E-33 |
| hsa-miR-199b-3p | 10328 | 603 | 88.76382148 | 11.23617852 | 0.126585115 | 0 |
| hsa-miR-126 | 245 | 12 | 90.40012158 | 9.599878416 | 0.106193202 | 7.29E-27 |
| hsa-miR-335 | 1395 | 65 | 90.82461549 | 9.175384514 | 0.101023103 | 6.92E-148 |
| hsa-miR-144 | 2811 | 125 | 91.20659702 | 8.793402975 | 0.096411918 | 4.01E-301 |
| hsa-miR-10a | 454 | 15 | 93.31547383 | 6.684526175 | 0.071633631 | 6.64E-55 |
| hsa-miR-144* | 4316 | 134 | 93.6931465 | 6.306853498 | 0.067313926 | 0 |
| hsa-miR-618 | 230 | 7 | 93.80986298 | 6.190137016 | 0.065985994 | 3.66E-29 |
| hsa-miR-199b-5p | 1062 | 21 | 95.88902136 | 4.110978643 | 0.042872256 | 1.86E-141 |
| hsa-miR-143 | 6537 | 128 | 95.92754431 | 4.072455693 | 0.042453455 | 0 |
| hsa-miR-223 | 112309 | 1912 | 96.4402997 | 3.559700301 | 0.036910921 | 0 |
| hsa-miR-582-5p | 405 | 5 | 97.39309792 | 2.606902075 | 0.026766805 | 9.31E-59 |
| hsa-miR-1277 | 242 | 2 | 98.23971384 | 1.760286162 | 0.017918275 | 4.69E-37 |
| hsa-miR-145* | 206 | 1 | 98.95848062 | 1.041519378 | 0.010524812 | 8.13E-33 |
