# Supplementary material for: Genome-Wide Analysis of Small RNA and Novel MicroRNA Discovery in Human Acute Lymphoblastic Leukemia Based on Extensive Sequencing Approach
Source: PLoS One. 2009 Sep 2;4(9):e6849. doi: 10.1371/journal.pone.0006849 (PMC2731166; doi:10.1371/journal.pone.0006849)
Supplement: Table S5 — (0.22 MB DOC) [file pone.0006849.s005.doc]

**Table S5. 171 known and novel miRNAs significantly different expression between the normal donors and patient group (fold changes >2.0 and a *p*-value of <0.001)**

| MiRNA | Fold changes | P-value |
| --- | --- | --- |
| hsa-miR-223 | 0.036932819 | 0 |
| hsa-miR-143 | 0.042478641 | 0 |
| hsa-miR-144* | 0.06735386 | 0 |
| hsa-miR-199b-3p | 0.126660213 | 0 |
| hsa-miR-451 | 0.174175781 | 0 |
| hsa-miR-27a | 0.227314393 | 0 |
| hsa-miR-142-3p | 0.238829299 | 0 |
| hsa-miR-23a | 0.268435546 | 0 |
| hsa-miR-101 | 0.30234555 | 0 |
| hsa-miR-103 | 2.026235546 | 0 |
| hsa-miR-107 | 2.90512111 | 0 |
| hsa-miR-92a | 3.254306829 | 0 |
| hsa-miR-342-3p | 4.057359596 | 0 |
| hsa-miR-363 | 5.499824332 | 0 |
| hsa-miR-181b | 5.725478324 | 0 |
| hsa-miR-128 | 7.145899914 | 0 |
| hsa-miR-181a | 7.638758413 | 0 |
| hsa-miR-9 | 74.91652603 | 0 |
| hsa-miR-144 | 0.096469116 | 4.0146E-301 |
| hsa-let-7d | 2.06825063 | 1.4228E-299 |
| hsa-miR-192 | 2.317609028 | 2.272E-263 |
| hsa-miR-181a* | 9.250069776 | 5.7273E-261 |
| hsa-miR-130b | 5.615196588 | 2.1159E-245 |
| hsa-miR-378 | 2.405030796 | 8.654E-245 |
| hsa-miR-424 | 0.176316945 | 4.5731E-224 |
| hsa-miR-30e* | 0.236970178 | 4.7222E-206 |
| hsa-miR-148a | 0.221812346 | 2.7259E-203 |
| hsa-miR-122 | 29.25587448 | 4.3884E-190 |
| hsa-miR-223* | 0.3333114 | 2.9187E-170 |
| hsa-miR-486-5p | 0.187347377 | 9.3982E-158 |
| hsa-miR-9* | 330.8331145 | 3.1706E-150 |
| hsa-miR-335 | 0.101083036 | 6.9234E-148 |
| hsa-miR-92a-1* | 22.10440127 | 8.3735E-144 |
| hsa-miR-199b-5p | 0.04289769 | 1.8585E-141 |
| hsa-miR-20a | 2.424620704 | 8.8159E-140 |
| hsa-miR-374a* | 0.179874665 | 5.4758E-127 |
| hsa-miR-17 | 2.254190518 | 3.1174E-123 |
| hsa-miR-19b | 0.301192144 | 1.8979E-109 |
| hsa-miR-542-3p | 0.222582689 | 3.70696E-95 |
| hsa-miR-130a | 2.790563079 | 6.71009E-93 |
| hsa-miR-181d | 2.998251087 | 5.94859E-82 |
| hsa-miR-499-5p | 4.310982156 | 5.00197E-81 |
| hsa-miR-30a | 0.174840093 | 2.32407E-77 |
| hsa-miR-17* | 3.745776258 | 9.66242E-76 |
| hsa-miR-598 | 5.226937533 | 8.3327E-74 |
| hsa-miR-181c | 4.859450337 | 1.93872E-65 |
| hsa-miR-1295 | #DIV/0! | 1.40732E-64 |
| hsa-miR-193b* | 69.96306847 | 1.25753E-59 |
| hsa-miR-582-5p | 0.026782685 | 9.3067E-59 |
| hsa-let-7c | 0.303063484 | 1.35054E-58 |
| hsa-miR-181a-2* | 9.663679648 | 2.2893E-58 |
| hsa-miR-10a | 0.071676128 | 6.64003E-55 |
| hsa-miR-25* | 7.390659184 | 1.54869E-52 |
| hsa-miR-1842 | 0.024443915 | 3.31E-52 |
| hsa-mir-766-5p | 14.77256374 | 2.97E-50 |
| hsa-miR-425 | 0.417191822 | 4.80034E-45 |
| hsa-miR-124 | #DIV/0! | 5.77968E-44 |
| hsa-miR-532-5p | 0.174402542 | 9.86168E-43 |
| hsa-miR-548a-3p | 9.334983061 | 3.29612E-42 |
| hsa-miR-1246 | 94.36879003 | 1.08596E-41 |
| hsa-miR-450a | 0.370596371 | 1.51049E-40 |
| hsa-miR-338-3p | 0.224287083 | 1.89644E-39 |
| hsa-miR-1277 | 0.017928905 | 4.68741E-37 |
| hsa-let-7e | 4.382182893 | 6.44762E-37 |
| hsa-miR-143* | 0.139551884 | 7.15923E-33 |
| hsa-miR-145* | 0.010531056 | 8.13413E-33 |
| hsa-miR-130b* | 5.094797093 | 1.94505E-32 |
| hsa-miR-618 | 0.06602514 | 3.66247E-29 |
| hsa-miR-145 | 0.01253987 | 1.74582E-27 |
| hsa-miR-196b | 0.024239078 | 5.43398E-27 |
| hsa-miR-342-5p | 3.790736635 | 6.78614E-27 |
| hsa-miR-126 | 0.106256203 | 7.29428E-27 |
| hsa-mir-181b-1* | 6.705410368 | 3.20E-26 |
| hsa-miR-99a | 0.047942486 | 7.57639E-25 |
| hsa-miR-625* | 30.37156461 | 2.62006E-24 |
| hsa-miR-193b | 55.31963553 | 4.91534E-24 |
| hsa-miR-629 | 5.804604046 | 2.15049E-23 |
| hsa-miR-206 | 99.79228371 | 7.78809E-23 |
| hsa-miR-1274b | 0.353263054 | 1.17737E-21 |
| hsa-miR-106a | 3.221226549 | 2.96718E-20 |
| hsa-miR-18a | 2.345294564 | 3.00445E-20 |
| hsa-miR-1890 | 14.64343294 | 4.51E-20 |
| hsa-miR-34c-5p | 0.01721744 | 6.41792E-20 |
| hsa-miR-1271 | 12.5342965 | 1.66066E-18 |
| hsa-mir-1307-5p | 0.169318827 | 1.86E-18 |
| hsa-miR-504 | 0 | 2.9641E-18 |
| hsa-miR-1 | 2.675046507 | 1.19946E-17 |
| hsa-miR-21* | 0.260664037 | 6.49419E-17 |
| hsa-miR-1943 | #DIV/0! | 8.15E-17 |
| hsa-miR-320b | 2.664771698 | 8.68633E-17 |
| hsa-miR-30a* | 0.021062111 | 3.09521E-16 |
| hsa-miR-582-3p | 0 | 1.73627E-14 |
| hsa-miR-24-2* | 0.150652602 | 3.18449E-14 |
| hsa-mir-301a-5p | 4.755986765 | 1.66E-12 |
| hsa-miR-425* | 0.386331057 | 2.33101E-12 |
| hsa-miR-941 | 0.105310557 | 3.66056E-12 |
| hsa-miR-548e | 0.492131834 | 1.05236E-11 |
| hsa-miR-590-3p | 0.287654913 | 2.49743E-11 |
| hsa-miR-365 | 0.054921455 | 2.71638E-11 |
| hsa-miR-651 | 0.24262998 | 3.26378E-11 |
| hsa-miR-1971 | 0.190906978 | 4.38E-11 |
| hsa-miR-625 | 2.881231017 | 5.28014E-11 |
| hsa-miR-181c* | 3.988892126 | 6.46082E-11 |
| hsa-miR-197 | 0.172783869 | 9.97237E-11 |
| hsa-miR-221* | 0.452512356 | 1.0338E-10 |
| hsa-miR-576-3p | 0.226653467 | 1.46128E-10 |
| hsa-miR-19a* | 6.508192416 | 2.90695E-10 |
| hsa-miR-577 | 13.5587342 | 5.50186E-10 |
| hsa-miR-1947 | 0.216939747 | 6.78E-10 |
| hsa-mir-365-1-5p | #DIV/0! | 8.92E-10 |
| hsa-miR-106a* | 4.158011821 | 1.87677E-09 |
| hsa-miR-190b | 0.149613619 | 3.51148E-09 |
| hsa-miR-210 | 5.42349368 | 4.57113E-09 |
| hsa-miR-1859* | 0.251004666 | 5.31E-09 |
| hsa-miR-1301 | 3.418444501 | 7.97642E-09 |
| hsa-miR-1852 | 0.069980564 | 1.05E-08 |
| hsa-miR-95 | 2.95826928 | 1.56451E-08 |
| hsa-miR-20b* | 4.459317026 | 2.36598E-08 |
| hsa-miR-500* | 2.301677806 | 2.9666E-08 |
| hsa-miR-1842* | 0 | 4.25E-08 |
| hsa-miR-7-1* | 2.036188855 | 6.68886E-08 |
| hsa-miR-1303 | 32.54096208 | 1.08116E-07 |
| hsa-miR-664 | 0.083438364 | 3.30437E-07 |
| hsa-miR-20a* | 2.621355279 | 3.3088E-07 |
| hsa-miR-652 | 0.309913925 | 4.22367E-07 |
| hsa-miR-421 | 2.096271714 | 4.99277E-07 |
| hsa-miR-1834 | 0 | 5.96E-07 |
| hsa-miR-1859 | 0.294869559 | 6.11E-07 |
| hsa-miR-1258 | #DIV/0! | 9.26355E-07 |
| hsa-miR-886-3p | 0.172174403 | 1.34644E-06 |
| hsa-miR-1971* | 0 | 1.85E-06 |
| hsa-miR-550* | 0.052912133 | 1.87072E-06 |
| hsa-miR-1852* | 0.054234937 | 2.67E-06 |
| hsa-miR-30d* | 4.028881019 | 6.1414E-06 |
| hsa-miR-1843 | 0.100902208 | 6.98E-06 |
| hsa-miR-450b-3p | 0.058632364 | 7.70919E-06 |
| hsa-miR-1902 | 10.12385487 | 8.25E-06 |
| hsa-miR-19b-1* | #DIV/0! | 9.38241E-06 |
| hsa-miR-137 | #DIV/0! | 9.38241E-06 |
| hsa-miR-18b | 2.616038128 | 1.44719E-05 |
| hsa-miR-335* | 3.254096208 | 1.47552E-05 |
| hsa-miR-501-3p | 3.525270892 | 2.26433E-05 |
| hsa-miR-27b* | 3.718967095 | 2.7197E-05 |
| hsa-miR-184 | #DIV/0! | 2.98595E-05 |
| hsa-miR-454 | 0.414157699 | 3.72219E-05 |
| hsa-miR-505* | 2.479311396 | 3.90054E-05 |
| hsa-miR-548j | 2.060927598 | 4.49457E-05 |
| hsa-miR-574-3p | 0.117264728 | 5.1295E-05 |
| hsa-miR-641 | 2.634268359 | 6.29881E-05 |
| hsa-miR-23b* | 7.050541784 | 7.01512E-05 |
| hsa-miR-345 | 0.433879494 | 7.29147E-05 |
| hsa-miR-573 | 19.52457725 | 7.37253E-05 |
| hsa-miR-18a* | 19.52457725 | 7.37253E-05 |
| hsa-miR-584 | 0.074806809 | 0.000126975 |
| hsa-miR-140-5p | 0.127611616 | 0.000137059 |
| hsa-miR-1986 | 0.131478635 | 0.000189704 |
| hsa-miR-942 | 2.386337219 | 0.000255978 |
| hsa-miR-153 | #DIV/0! | 0.000302427 |
| hsa-miR-505 | 0.371896709 | 0.000321308 |
| hsa-miR-628-5p | 0.222502305 | 0.000463336 |
| hsa-miR-1255a | 0.25225552 | 0.000468936 |
| hsa-miR-574-5p | 0.144626498 | 0.000498687 |
| hsa-miR-1866 | 0.144626498 | 0.000498687 |
| hsa-mir-181b-2* | 3.471035955 | 0.0007279 |
| hsa-miR-1893 | 0 | 0.000771849 |
| hsa-miR-1254 | 8.677589888 | 0.000846081 |
| hsa-miR-200a | 3.796445576 | 0.000882212 |
| hsa-miR-1841 | #DIV/0! | 0.000962473 |
| hsa-miR-1931 | #DIV/0! | 0.000962473 |
| hsa-miR-1987 | #DIV/0! | 0.000962473 |
| hsa-mir-619-5p | #DIV/0! | 0.000962473 |
