# Supplementary material for: Genome-Wide Analysis of Small RNA and Novel MicroRNA Discovery in Human Acute Lymphoblastic Leukemia Based on Extensive Sequencing Approach
Source: PLoS One. 2009 Sep 2;4(9):e6849. doi: 10.1371/journal.pone.0006849 (PMC2731166; doi:10.1371/journal.pone.0006849)
Supplement: Figure S1 — Alignment of 159 novel miRNAs in C group and P group(q means the read comes from C group; t means the read comes from P group) (0.29 MB DOC) [file pone.0006849.s006.doc]

>hsa-miR-1828 chr1 1719802 1719881 - mfe=-31.300

GGCGGCCCTTGCCTGGATCATTCTGATCAAGATGGCTACAGATACATAAGCATTGATCAGAAGTCCAGCCTAAGGCCTTC

...((((...(.((((((..((((((((((....(((...........))).)))))))))))))))).)...))))...

**************************************************CATTGATCAGAAGTCCAGCCTA******** hsa-miR-1828 **number of reads**

..................................................CATTGATCAGAAGTCCAGCCTA........ q0034784 2

..................................................CATTGATCAGAAGTCCAGCCT......... t0039392 1

>hsa-miR-1829 chr1 27080220 27080318 - mfe=-29.500 AATGCATTTTCGACTTAAGATATTTTTGATTTAGGATGGGTTTATCAGGATGTAACCCCATCATAAGTCAAGGAGCATCTGTACGTTACTTTATGTATT

(((((((....(((...((((.(((((((((((.((((((.((((......)))).)))))).)))))))))))..))))....))).....)))))))

********************TATTTTTGATTTAGGATGGGTT********************************************************* hsa-miR-1829

....................TATTTTTGATTTAGGATGGGTT......................................................... q0034390 2

....................TATTTTTGATTTAGGATGGG........................................................... t0029820 1

>hsa-miR-1830 chr1 44783736 44783826 + mfe=-34.500

AATCCAAGATCCATAACAGGGAAATGGGAAGAACTAGATTTGAATCCAGACCTTTAGTTCTTCCCTTTGCCCAATTATGCTCCTTGTGGTT

((((((((...(((((..(((....(((((((((((((((((....))))..)))))))))))))....)))..)))))...)))).))))

****************CAGGGAAATGGGAAGAACTAGA***************************************************** hsa-miR-1830

................CAGGGAAATGGGAAGAACTAGA..................................................... q0032941 2

................CAGGGAAATGGGAAGAACTAG...................................................... q0036739 2

>hsa-miR-1831 chr1 61839160 61839246 - mfe=-39.110

TAGGTTAGTGCAAAAGTAATTGTGGTTTTTGTGATTACTTGTTTTTTTAAAAATAGCAAAAACCACAATTACTTTTGCACCAACGTA

((.(((.(((((((((((((((((((((((((.......((((((....))))))))))))))))))))))))))))))).))).))

***********AAAAGTAATTGTGGTTTTTGTG****************************************************** hsa-miR-1831

...........AAAAGTAATTGTGGTTTTTGTG...................................................... q0008077_t0050435 9_1

...........AAAAGTAATTGTGGTTTTTGTGA..................................................... q0079945 1

>hsa-miR-1832 chr1 63739341 63739428 - mfe=-46.400

GGTAGGGACTGGATTCATTATTTTGCATGTGTATAACCAGTTACTGAAACAGATATCCACATGTAAAAAAATGAATCTAGTCCCTACC

(((((((((((((((((((.(((((((((((.(((.(..(((.....))).).))).))))))))))).)))))))))))))))))))

*******************************************************TCCACATGTAAAAAAATGAATC*********** hsa-miR-1832

*********TGGATTCATTATTTTGCATGTG********************************************************* hsa-miR-1832*

.......................................................TCCACATGTAAAAAAATGAATC........... q0010389 7

.........TGGATTCATTATTTTGCATGTG......................................................... q0028282_t0047280 2_1

.........TGGATTCATTATTTTGCATGTGT........................................................ q0110845_t0057421 1_1

.........TGGATTCATTATTTTGCATGT.......................................................... t0011566 2

>hsa-miR-1833 chr1 67223427 67223489 + mfe=-36.500

GAATATCTTTAGTTCATTCAGATTTACTAATTTTGGTAAATCTGAATGAACTAAAGATGTATC

..(((((((((((((((((((((((((((....)))))))))))))))))))))))))))...

**************************************AATCTGAATGAACTAAAGATGT*** hsa-miR-1833

......................................AATCTGAATGAACTAAAGATGT... q0033708 2

.......................................ATCTGAATGAACTAAAGATGTA.. q0061264 1

>hsa-miR-1834 chr1 74045231 74045292 - mfe=-18.100

TGATCACCATTCTAACTGGTGTGAGATGATATCTAGTTGTTTCCATAGTGTAGTGGTTATCA

.(((.((((((...((((.((.((((..((.....))..))))))))))..)))))).))).

**************************************GTTTCCATAGTGTAGTGG****** hsa-miR-1834

......................................GTTTCCATAGTGTAGTGG...... q0005166 16

......................................GTTTCCATAGTGTAGTGGTTAT.. q0006462 12

......................................GTTTCCATAGTGTAGTGGTTATC. q0015508 4

.......................................TTTCCATAGTGTAGTGGTT.... q0030381 2

......................................GTTTCCATAGTGTAGTGGT..... q0058541 1

......................................GTTTCCATAGTGTAGTGGTTATCA q0059490 1

......................................GTTTCCATAGTGTAGTGGTTA... q0090896 1

>hsa-miR-1835 chr5_h2_hap1 1579412 1579508 + mfe=-52.800

AGTAGCTGGGATTACAGGCATGTGCTACCCTGCTTGGCTTGTTTTAAAAATGCATTTCAAGGCAGACGCGGTGGCTCACGCCTGTAATCCCAGCACT

(((.(((((((((((((((.((.((((((((((((((..(((.........)))..)))).))))....)))))).)).))))))))))))))))))

*******************ATGTGCTACCCTGCTTGGCTTG******************************************************** hsa-miR-1835

...................ATGTGCTACCCTGCTTGGCTTG........................................................ q0031567_t0022060 2_1

>hsa-miR-1836 chr1 116904170 116904255 - mfe=-49.500

TATTAGGTTGGTGCAAAAGTTATTGTGGTTTTTGCTATTTTTTTTTAATGGCAAAAACCGGCAATTACTTTTGCACTAACCTAGTA

((((((((((((((((((((.((((((((((((((((((.......))))))))))))).))))).))))))))))))))))))))

**************AAAAGTTATTGTGGTTTTTGCT************************************************** hsa-miR-1836

*****************************************************AAAACCGGCAATTACTTTTGCA*********** hsa-miR-1836*

..............AAAAGTTATTGTGGTTTTTGCT.................................................. q0008722 8

....................................................AAAAACCGGCAATTACTTT............... q0053553 1

....................................................AAAAACCGGCAATTACTTTTG............. q0080107 1

...................................................CAAAAACCGGCAATTACTTTTG............. q0084943 1

.....................................................AAAACCGGCAATTACTTTTGCA........... t0031077 1

.....................................................AAAACCGGCAATTACTTTTGC............ t0032264 1

>hsa-miR-1837 chr1 147589963 147590046 + mfe=-55.800

TATTAGGTTGGTGCAAAAGTAACTGCGGTTTTTGTCATTACTTTTAATGGCAAAAATCGCAATTACTTTTGCACCAACCTAATA

((((((((((((((((((((((.(((((((((((((((((....))))))))))))))))).))))))))))))))))))))))

**************AAAAGTAACTGCGGTTTTTGT************************************************* hsa-miR-1837

..............AAAAGTAACTGCGGTTTTTGT................................................. q0031378 2

..............AAAAGTAACTGCGGTTTTTGTCA............................................... q0081875 1

>hsa-miR-1838 chr1 147975492 147975575 + mfe=-55.800

TATTAGGTTGGTGCAAAAGTAACTGCGGTTTTTGTCATTACTTTTAATGGCAAAAATCGCAATTACTTTTGCACCAACCTAATA

((((((((((((((((((((((.(((((((((((((((((....))))))))))))))))).))))))))))))))))))))))

**************AAAAGTAACTGCGGTTTTTGT************************************************* hsa-miR-1838

..............AAAAGTAACTGCGGTTTTTGT................................................. q0031378 2

..............AAAAGTAACTGCGGTTTTTGTCA............................................... q0081875 1

>hsa-miR-1839 chr1 182019086 182019177 + mfe=-32.800

AATGATACTTAGATTGGCACAAAAGTAACTGTGGTTTTGCATTGAAAGTATAGCAAAAACCACAATTACTTTTGCACCAATCCAATATTATT

(((((((.((.((((((..(((((((((.(((((((((((............)).))))))))).)))))))))..)))))).)))))))))

*******************CAAAAGTAACTGTGGTTTTGCA*************************************************** hsa-miR-1839

...................CAAAAGTAACTGTGGTTTTGCA................................................... q0012219 5

>hsa-miR-1840 chr1 222652547 222652642 - mfe=-33.800

ATTTCATCAGGCAAAGGGATATTTACAGATACTTTTTAAAATTTGTTTGAGTTGAGGCAGATTAAATATCTGTATTCTCCTTTGCCTGCAGGGAGT

(((((..((((((((((((....((((((((.(((....(((((((((......)))))))))))))))))))).)).))))))))))...)))))

********************************************************************TCTGTATTCTCCTTTGCCTGCA****** hsa-miR-1840

....................................................................TCTGTATTCTCCTTTGCCTGCA...... q0017351 3 3

......TCAGGCAAAGGGATATTTACAG.................................................................... q0070874_t0054669 1_1

......TCAGGCAAAGGGATATTTACAGA................................................................... t0032745 1

......TCAGGCAAAGGGATATTTACAGAT.................................................................. t0041568 1

>hsa-miR-1841 chr1 233419967 233420059 - mfe=-51.900

GAAATATATCTACACAAGGCCAAAGGAAGAGAACAGATATATCCACAGTACACTTGGCTGTTCTCTTTCTTTAGCCTTGTGTAGATATATTTT

((((((((((((((((((((.(((((((((((((((......(((.((....)))))))))))))))))))).))))))))))))))))))))

**************CAAGGCCAAAGGAAGAGAACAG********************************************************* hsa-miR-1841

..............CAAGGCCAAAGGAAGAGAACAG......................................................... t0010130 3

..............CAAGGCCAAAGGAAGAGAACA.......................................................... t0011463 2

>hsa-miR-1842 chr1 234082906 234083001 - mfe=-46.400

TGGGAGTTGGCACTAAGTACAGCTGTAATTAGTCAGTTTTCTGTCCTGTCCACACAGAAAACCGTCTAGTTACAGTTGTAAGTTGTGCCAGACCTA

((((..(((((((....(((((((((((((((.(.(((((((((.........))))))))).).)))))))))))))))....))))))).))))

*********************************************************AAAACCGTCTAGTTACAGTTGT***************** hsa-miR-1842

**********************CTGTAATTAGTCAGTTTTCTGT**************************************************** hsa-miR-1842*

.........................................................AAAACCGTCTAGTTACAGTTGT................. q0000592_t0006546 279_4

.........................................................AAAACCGTCTAGTTACAGTTG.................. q0001920 58

......................CTGTAATTAGTCAGTTTTCTGT.................................................... q0005839 14

....................AGCTGTAATTAGTCAGTTTTC....................................................... q0007746 10

....................AGCTGTAATTAGTCAGTTTT........................................................ q0007980 9

....................AGCTGTAATTAGTCAGTTTTCT...................................................... q0008139 9

..........................................................AAACCGTCTAGTTACAGTTGT................. q0010287 7

.........................................................AAAACCGTCTAGTTACAGTTGTA................ q0014877 4

..........................................................AAACCGTCTAGTTACAGTT................... q0026084 2

.........................................................AAAACCGTCTAGTTACAGTT................... q0031125 2

.........................................................AAAACCGTCTAGTTACAGT.................... q0032181 2

.........................................................AAAACCGTCTAGTTACAG..................... q0069797 1

......................CTGTAATTAGTCAGTTTTCTG..................................................... q0079756 1

....................AGCTGTAATTAGTCAGTTT......................................................... q0085525 1

>hsa-miR-1843 chr2 6912669 6912761 - mfe=-60.600

GGGAATTATTAGGTTGGTGCAAAAGTCATTGTGGTTTTTGATATTATTATTAATGGCAAAAACCGCAATGACTTTTGCACCAACCTAATACCC

(((...((((((((((((((((((((((((((((((((((.(((((....))))).)))))))))))))))))))))))))))))))))))))

********************AAAAGTCATTGTGGTTTTTGAT*************************************************** hsa-miR-1843

....................AAAAGTCATTGTGGTTTTTGAT................................................... q0003940_t0052992 23_1

...................CAAAAGTCATTGTGGTTTTTGA.................................................... q0004979 17

....................AAAAGTCATTGTGGTTTTTGATA.................................................. q0026340 2

....................AAAAGTCATTGTGGTTTTTGA.................................................... q0101324_t0046100 1_1

>hsa-miR-1844 chr2 11825019 11825105 + mfe=-40.400

GCTTGCTGCAAAAATAATTGCAGTTTTTGCCATTATTTTTAATAATTATAATAATGGCCAAAACTGCAGTTATTTTTGCACCAACGT

(((((.(((((((((((((((((((((.(((((((((............))))))))).))))))))))))))))))))).))).))

*****************************************************ATGGCCAAAACTGCAGTTATTT************ hsa-miR-1844

.....................................................ATGGCCAAAACTGCAGTTATTT............ q0050866 1

.....................................................ATGGCCAAAACTGCAGTTATT............. q0058107 1

...................................................TAATGGCCAAAACTGCAGTTA............... t0038754 1

...................................................TAATGGCCAAAACTGCAGTTAT.............. t0058325 1

>hsa-miR-1845 chr2 16476213 16476290 - mfe=-40.100

AATTCATTTAATCCTCACTTTGAATCCATGTTGGAAACTCCATAGCAACATGGAGTTCAGGTGAGGATTAAATGAGTT

((((((((((((((((((((.((((((((((((............))))))))).)))))))))))))))))))))))

***********************************************ACATGGAGTTCAGGTGAGGATT********* hsa-miR-1845

*********AATCCTCACTTTGAATCCAT************************************************* hsa-miR-1845*

...............................................ACATGGAGTTCAGGTGAGGATT......... q0067739 1

.........AATCCTCACTTTGAATCCAT................................................. t0022431 1

>hsa-miR-1846 chr2 27398096 27398171 - mfe=-35.400

GGTTAGTATATTGGTATCTGATCACTCCATCCTGATCCATTTGGAGTGATCAGATACCAGTCACTCCAAATGCAGT

((..(((..((((((((((((((((((((............)))))))))))))))))))).))))).........

*******ATATTGGTATCTGATCACTCCA*********************************************** hsa-miR-1846

.......ATATTGGTATCTGATCACTCCA............................................... q0021876 3

>hsa-miR-1847 chr2 35549972 35550068 + mfe=-44.500

CAACTGTTAGGTTGGTGCAAAAGTAATTGTGGTTTTTGAAAGTAACTTGGCGAAAACGACAATGACTTTTGCACCAATCTAATACAATCAACAGTTG

((((((((((((((((((((((((.(((((.(((((((.(((...)))..))))))).))))).))))))))))))))))).........)))))))

******************AAAAGTAATTGTGGTTTTTGAA********************************************************* hsa-miR-1847

..................AAAAGTAATTGTGGTTTTTGAA......................................................... q0006517 12

..................AAAAGTAATTGTGGTTTTTGAAA........................................................ q0018932 3

.................CAAAAGTAATTGTGGTTTTTGAA......................................................... q0037828_t0058090 2_1

.................CAAAAGTAATTGTGGTTTTTGAAA........................................................ q0068856 1

>hsa-miR-1848 chr2 64421389 64421487 - mfe=-73.740

CTGTGTTCCCTATCCTCCTTATGTCCCACCCCCACTCCTGTTTGAATATTTCACCAGAAACAGGAGTGGGGGGTGGGACGTAAGGAGGATGGGGGAAAG

.....((((((((((((((((((((((((((((((((((((((..............)))))))))).))))))))))))))))))))))))))))...

************************************************************CAGGAGTGGGGGGTGGGACGTA***************** hsa-miR-1848

............................................................CAGGAGTGGGGGGTGGGACGTA................. q0017824 3

...........................................................ACAGGAGTGGGGGGTGGGACGTA................. q0083562 1

>hsa-miR-1849 chr2 68906547 68906625 + mfe=-32.100

AGGTTGTGAGCAAGGGGGAAGGAAGAGTCTAGAATGAATGCCACATTCTTTCATTCTTTTCTTTCCTTCCTTGTAGCCT

((((((..((...((((((((((((((....(((.(((((...))))).)))...))))))))))))))))..))))))

*****************************************************TTCTTTTCTTTCCTTCCTTGTAG*** hsa-miR-1849

.....................................................TTCTTTTCTTTCCTTCCTTGTAG... q0081002 1

....................................................ATTCTTTTCTTTCCTTCCTTGT..... t0015132 2

>hsa-miR-1850 chr2 71607460 71607555 + mfe=-48.410

CCCACCATCCTAGCTTGCCTGAGACTGTCCTGGTTTTAGCACTGAAAGTGCCTGTTCCAGGAAACCCTGCAGTCTCCAGCAAACTGGGACAGTGGG

(((((..((((((.((((..(((((((((((((..(..((((.....))))..)..))))).......))))))))..)))).))))))..)))))

******ATCCTAGCTTGCCTGAGACTGT******************************************************************** hsa-miR-1850

......ATCCTAGCTTGCCTGAGACTGT.................................................................... q0031741 2

.....CATCCTAGCTTGCCTGAGACTG..................................................................... q0034964 2

.....CATCCTAGCTTGCCTGAGACTGT.................................................................... q0067437 1

>hsa-miR-1851 chr22 48323039 48323125 - mfe=-56.300

GCTTCTCTGAGGATGAAAGACCCATTGAGGAGAAGGTTCTGCTGGCTGAGAACCTTCCTCTCCATGGGTCTTTCATCCTCAAAGAAC

..((((.((((((((((((((((((.(((..(((((((((........)))))))))..))).)))))))))))))))))).)))).

*****************AGACCCATTGAGGAGAAGGTTC************************************************ hsa-miR-1851

***************************************************ACCTTCCTCTCCATGGGTCTT*************** hsa-miR-1851*

.................AGACCCATTGAGGAGAAGGTTC................................................ q0020269 3

...................................................ACCTTCCTCTCCATGGGTCTT............... q0040983 1

.................AGACCCATTGAGGAGAAGGTT................................................. q0042551 1

>hsa-miR-1852 chr2 102415176 102415263 + mfe=-66.600

GCTCTGTGATTGCCTCTGATCAGGCAAAATTGCAGACTGTCTTCCCAAATAGCCTGCAACTTTGCCTGATCAGAGGCAGTCACAGAGC

((((((((((((((((((((((((((((.((((((.((((........)))).)))))).))))))))))))))))))))))))))))

****************************************************CCTGCAACTTTGCCTGATCAGA************** hsa-miR-1852

****************TGATCAGGCAAAATTGCAGACT************************************************** hsa-miR-1852*

....................................................CCTGCAACTTTGCCTGATCAGA.............. q0002306_t0014559 46_2

................TGATCAGGCAAAATTGCAGACT.................................................. q0004463_t0042256 20_1

..............TCTGATCAGGCAAAATTGCAGA.................................................... q0005514 15

....................................................CCTGCAACTTTGCCTGATCAG............... q0007597 10

..............TCTGATCAGGCAAAATTGCAG..................................................... q0019672 3

......................................................TGCAACTTTGCCTGATCAGAGG............ q0020198 3

................TGATCAGGCAAAATTGCAGA.................................................... q0028687 2

.....................................................CTGCAACTTTGCCTGATCAGA.............. q0029647 2

....................................................CCTGCAACTTTGCCTGATCA................ q0092610 1

>hsa-miR-1853 chr2 126329376 126329466 + mfe=-51.000

ATTCTCTTAGGTTGGTGCAAAAGTAGTTGTGGTTTTGCCATTCATTTCAGTGATAAAAACCGCAATTACTTTTGCACCAACCTAATCGAAT

((((..((((((((((((((((((((((((((((((.....((((....))))..))))))))))))))))))))))))))))))..))))

*****************CAAAAGTAGTTGTGGTTTTGC***************************************************** hsa-miR-1853

.................CAAAAGTAGTTGTGGTTTTGC..................................................... q0027191 2

.................CAAAAGTAGTTGTGGTTTTGCC.................................................... q0102813 1

>hsa-miR-1854 chr2 137119141 137119236 - mfe=-42.000

TCTGGCATTAGGTTGGTACAAAAGTAATTGCGGATTTTGTCATTACTTTCAATGGCAAAAACCACAATTACTTTTGCACCAGCCTCATAGGAGAGA

(((...((.((((((((.((((((((((((.((.((((((((((......)))))))))).)).)))))))))))).)))))))).)).)))....

*******************AAAAGTAATTGCGGATTTTGT******************************************************** hsa-miR-1854

...................AAAAGTAATTGCGGATTTTGT........................................................ q0018416 3

...................AAAAGTAATTGCGGATTTTGTC....................................................... t0034299 1

>hsa-miR-1855 chr2 140300734 140300821 + mfe=-43.900

GTTATTAGAATGGTGCAAAAGTAATTGTTGTTCTTGCCTTTGAAAGTAATGGCAAAAACGACAATTACTTTTGTGCCAACCTAATAAT

((((((((..(((..(((((((((((((((((.(((((.(((....))).))))).)))))))))))))))))..)))..))))))))

****************AAAAGTAATTGTTGTTCTTGCC************************************************** hsa-miR-1855

****************************************************CAAAAACGACAATTACTTTTG*************** hsa-miR-1855*

................AAAAGTAATTGTTGTTCTTGCC.................................................. t0010085 3

....................................................CAAAAACGACAATTACTTTTG............... t0027634 1

>hsa-miR-1856 chr2 175172963 175173059 - mfe=-44.560

TCAGATCAGCTGCGCATTAGATTCTCAGAGGAGCAAGAATCCTAAAGTGAACTGTGCATGCGAGCTCCTTATGAGAATCTAATGCCTGATGATCTGA

((((((((...(.(((((((((((((((((((((..(........((....)).......)..))))))).)))))))))))))))...))))))))

**********TGCGCATTAGATTCTCAGAGGA***************************************************************** hsa-miR-1856

..........TGCGCATTAGATTCTCAGAGGA................................................................. t0008756 3

..........TGCGCATTAGATTCTCAGAGGAG................................................................ t0038010 1

>hsa-miR-1857 chr2 189853898 189853983 - mfe=-48.300

GTATTAGATTGGTGCAAAAGTTATTGTGGTTTTTGCTGTTACTTTCAGTGGCAAAAACTACAATAACTTTTGCACTGACCTAATAT

(((((((.(..(((((((((((((((((((((((((..((......))..)))))))))))))))))))))))))..).)))))))

***************AAAAGTTATTGTGGTTTTTGCT************************************************* hsa-miR-1857

...............AAAAGTTATTGTGGTTTTTGCT................................................. q0008722 8

>hsa-miR-1858 chr2 207356199 207356287 - mfe=-72.800

TGTGACACAACTTGTCATGTCTTACCCAGTCTCCGGTGCAGCCTGTTGTCAAGGCTGCACCGGAGACTGGGTAAGACATGACAAGCACA

.(((......((((((((((((((((((((((((((((((((((.......))))))))))))))))))))))))))))))))))))).

*****************************************************GCTGCACCGGAGACTGGGTAA*************** hsa-miR-1858

.....................................................GCTGCACCGGAGACTGGGTAA............... q0012628 5

......................................................CTGCACCGGAGACTGGGTA................ q0048062 1

>hsa-miR-1859 chr2 207682956 207683044 - mfe=-35.200

GTCAGACGTGTCATCCCCAGATACAATGGACAATATGCTATTATAATCGTATGGCATTGTCCTTGCTGTTTGGAGATAATACTGCTGAC

(((((..((((.(((.((((((((((.(((((..(((((((.........))))))))))))))).))))))).))).))))..)))))

************ATCCCCAGATACAATGGACAAT******************************************************* hsa-miR-1859

*******************************************************ATTGTCCTTGCTGTTTGGAGAT************ hsa-miR-1859*

............ATCCCCAGATACAATGGACAAT....................................................... q0001660_t0003758 70_9

.......................................................ATTGTCCTTGCTGTTTGGAGAT............ q0002417_t0010535 43_2

............ATCCCCAGATACAATGGACAA........................................................ q0005977 13

.........................................................TGTCCTTGCTGTTTGGAGATA........... q0006781_t0011294 11_2

.......................................................ATTGTCCTTGCTGTTTGGAGA............. q0006968_t0007793 11_4

............ATCCCCAGATACAATGGACA......................................................... q0007122_t0007232 11_4

.........................................................TGTCCTTGCTGTTTGGAGATAA.......... q0007809 10

.......................................................ATTGTCCTTGCTGTTTGGAGATAA.......... q0007885_t0025115 9_1

.........................................................TGTCCTTGCTGTTTGGAGAT............ q0008372_t0013912 9_2

........................................................TTGTCCTTGCTGTTTGGAGAT............ q0010854_t0042069 6_1

............ATCCCCAGATACAATGGACAATA...................................................... q0012004 5

......................................................CATTGTCCTTGCTGTTTGGAGA............. q0012127 5

.......................................................ATTGTCCTTGCTGTTTGGAGATA........... q0012991 5

........................................................TTGTCCTTGCTGTTTGGAGATAA.......... q0014825 4

........................................................TTGTCCTTGCTGTTTGGAGATA........... q0019063_t0046066 3_1

...............CCCAGATACAATGGACAAT....................................................... q0029600 2

.......................................................ATTGTCCTTGCTGTTTGGAG.............. q0030314 2

..........................................................GTCCTTGCTGTTTGGAGAT............ q0043255 1

............ATCCCCAGATACAATGGACAATAT..................................................... q0050933 1

................CCAGATACAATGGACAAT....................................................... q0071179 1

.........................................................TGTCCTTGCTGTTTGGAGA............. q0095405 1

........................................................TTGTCCTTGCTGTTTGGAG.............. q0106183 1

............ATCCCCAGATACAATGGAC.......................................................... t0027350 1

........................................................TTGTCCTTGCTGTTTGGAGA............. t0032190 1

>hsa-miR-1860 chr2 208327771 208327855 + mfe=-39.000

TGAATATTAAGCTTTTAATTTTTTGTTTCGGTCACTCTTGATAGCAGACATTGACTGAAACAAAAAATTAAAAGCTTTATATTCA

.((((((.((((((((((((((((((((((((((.(((.......)))...)))))))))))))))))))))))))).)))))).

**************TTAATTTTTTGTTTCGGTCACT************************************************* hsa-miR-1860

..............TTAATTTTTTGTTTCGGTCACT................................................. q0014209_t0011547 4_2

..............TTAATTTTTTGTTTCGGTCA................................................... q0051403 1

...............TAATTTTTTGTTTCGGTCACT................................................. q0100872 1

>hsa-miR-1861 chr2 211078200 211078294 + mfe=-52.700

TCAACATATAGGTTGGTGCAAAAGTAATTGCAGTTTTTGCCATTGAAAGTAGTGGCCAAAACTGCAATTACTTTAGCACCAACCTAATATCATGA

........(((((((((((.(((((((((((((((((.(((((((....))))))).))))))))))))))))).))))))))))).........

*******************************************************CCAAAACTGCAATTACTTTAG******************* hsa-miR-1861

.......................................................CCAAAACTGCAATTACTTTAG................... q0021335 3

>hsa-miR-1862 chr2 239938346 239938444 - mfe=-56.100

AGACGCTTGGACAGGCACCTGAGGCTCTGTTAGCCTTGGCTCTGGGTCCTGCTCCTTAGAGCAGAGGCAGAGAGGCTCAGGGTCTGTCTGGGTCACTCT

....(((..(((((((.((((((.((((.(..(((((.((((((((........)))))))).)))))).)))).)))))))))))))..)))......

*******************TGAGGCTCTGTTAGCCTTGGCTCT******************************************************** hsa-miR-1862

...................TGAGGCTCTGTTAGCCTTGGCTCT........................................................ q0019586 3

>hsa-miR-1863 chr3 19331342 19331429 - mfe=-27.600

TTTAAGAACTGGATATGATGACTGAAATAAGCTCCATATCAATGAGAATTTCAATGGGATTATGTGCAGTCAATGTCCAGTAATTAGA

(((((..(((((((((..((((((..((((..(((((...(((....)))...))))).))))...)))))))))))))))..)))))

*********TGGATATGATGACTGAAA************************************************************* hsa-miR-1863

.........TGGATATGATGACTGAAA............................................................. q0019101 3

>hsa-miR-1864 chr3 31178195 31178288 - mfe=-53.700

TGTATGTGTGTGTATATGTGTGTTGCATGTGTGTATATGTGTGTATATATGTACACATACACATACACGCAACACACATATATACATGCACACA

.((.((((((((((((((((((((((.(((((((.((((((((........)))))))).))))))).)))))))))))))))))))))).)).

******************TGTGTTGCATGTGTGTATAT******************************************************** hsa-miR-1864

..................TGTGTTGCATGTGTGTATAT........................................................ t0008661 3

..................TGTGTTGCATGTGTGTAT.......................................................... t0052503 1

>hsa-miR-1865 chr3 38039376 38039471 + mfe=-58.200

TTAGTTTGGTGCAAAAGTAATCACGGTTTTTGCTATTGAAAGTAATAGCAAAAACTTTCAATAGCAAAAACTGTGATTACTTTTGCATCAATCTAA

.(((.((((((((((((((((((((((((((((((((((((((..........)))))))))))))))))))))))))))))))))))))).))).

*****************************************************************AAAAACTGTGATTACTTTTGCAT******** hsa-miR-1865

.................................................................AAAAACTGTGATTACTTTTGCAT........ q0021178 3

>hsa-miR-1866 chr3 48332858 48332955 - mfe=-44.800

TGACTGTCATCCCACTGCTTCCAGCTTCCATGACTCCTGATGGAGGAATCACATGAATTCATCAGAATTCATGGAGGCTAGAAGCAGTATGAGGATCA

.((.(.((((...((((((((.(((((((((((...((((((((....((....)).))))))))...))))))))))).)))))))))))).).)).

***********************************************************CATCAGAATTCATGGAGGCTAGA**************** hsa-miR-1866

**********************AGCTTCCATGACTCCTGATGGA****************************************************** hsa-miR-1866*

...........................................................CATCAGAATTCATGGAGGCTAGA................ q0006165 13

...........................................................CATCAGAATTCATGGAGGCTAG................. q0006492_t0047346 12_1

............................................................ATCAGAATTCATGGAGGCTAGA................ q0018601 3

......................AGCTTCCATGACTCCTGATGGA...................................................... q0021431 3

...........................................................CATCAGAATTCATGGAGGCTAGAA............... q0039194 1

......................AGCTTCCATGACTCCTGATG........................................................ q0069176 1

...........................................................CATCAGAATTCATGGAGGCT................... q0091055 1

.....................CAGCTTCCATGACTCCTGATGG....................................................... q0100724 1

......................AGCTTCCATGACTCCTGATGG....................................................... q0103270 1

...........................................................CATCAGAATTCATGGAGGCTA.................. t0042028 1

>hsa-miR-1867 chr3 69180794 69180881 - mfe=-45.500

AAATTAATATGAAACTGACTGAATAGGTAGGGTCATTTTTCTGTGACTGCACATGGCCCAACCTATTCAGTTAGTTCCATATTAGTTT

(((((((((((.((((((((((((((((.((((((......((((....)))))))))).)))))))))))))))).)))))))))))

**************CTGACTGAATAGGTAGGGTCAT**************************************************** hsa-miR-1867

..............CTGACTGAATAGGTAGGGTCAT.................................................... q0074287_t0058600 1_1

..............CTGACTGAATAGGTAGGGTCATT................................................... q0105125 1

>hsa-miR-1868 chr3 115944978 115945067 - mfe=-54.400

GGCAGTAAATTTGTGTCTATACTCTGTCACTTTACTTTTGGCCTCAAGTCATTGCAGTAAAGTGGCAGAGTATAGACACAAATTTAGGCC

(((..(((((((((((((((((((((((((((((((..((((.....))))....))))))))))))))))))))))))))))))).)))

*********************************************************TAAAGTGGCAGAGTATAGACA************ hsa-miR-1868

.........................................................TAAAGTGGCAGAGTATAGACA............ q0062755_t0044934 1_1

.........................................................TAAAGTGGCAGAGTATAG............... t0034370 1

>hsa-miR-1869 chr3 128788633 128788722 - mfe=-49.600

GTCAATGAGAATCTGCCCTGAGACTTTTGCTCTAATAATTTATTCTAATAATAATTTAGATCAAAAGCCTCAGGGCAGATTTTCATTGGC

((((((((((((((((((((((.((((((.(((((....((((....))))....))))).)))))).))))))))))))))))))))))

*************TGCCCTGAGACTTTTGCTCTAA******************************************************* hsa-miR-1869

.............TGCCCTGAGACTTTTGCTCTA........................................................ q0028439 2

.............TGCCCTGAGACTTTTGCTCT......................................................... q0040777 1

.............TGCCCTGAGACTTTTGCTCTAA....................................................... q0041142_t0019576 1_1

>hsa-miR-1870 chr3 149665108 149665194 - mfe=-38.500

TATTAGGTTGGTGCAAAAGTAATTGCGGTTTTTGACTTTTTTTTTTTTTAAGGCAAAAACCACAATTACTGTTGCACCAATATAATG

(((((.((((((((((.((((((((.((((((((.((((..........)))))))))))).)))))))).)))))))))).)))))

***************AAAGTAATTGCGGTTTTTGACTT************************************************* hsa-miR-1870

...............AAAGTAATTGCGGTTTTTGACTT................................................. q0014847 4

...............AAAGTAATTGCGGTTTTTGACT.................................................. q0016791 4

................AAGTAATTGCGGTTTTTGACTT................................................. q0053139 1

>hsa-miR-1871 chr3 152436422 152436513 + mfe=-54.400

AAAATATTAGGTTGGTGCAAAAGTAATTGTGGTTTTTGTGATGACTTTCAATGGCAAAAACTGCAATTACTTTTGCACCAATCTAATATTTT

(((((((((((((((((((((((((((((..((((((((.((........)).))))))))..)))))))))))))))))))))))))))))

******************AAAAGTAATTGTGGTTTTTGTG**************************************************** hsa-miR-1871

..................AAAAGTAATTGTGGTTTTTGTG.................................................... q0008077_t0050435 9_1

..................AAAAGTAATTGTGGTTTTTGTGA................................................... q0079945 1

>hsa-miR-1872 chr3 198505137 198505228 - mfe=-64.400

GCAGAAAAATCCGACTCAGAAGACAGAGTGCCACTTACTGAAAGGTTTTTTCTCTCAGTAAGTGGCACTCTGTCTTCTGAGTTGGTACCTGT

((((......(((((((((((((((((((((((((((((((.(((.....))).)))))))))))))))))))))))))))))))...))))

****************************************************TCTCAGTAAGTGGCACTCTGTC****************** hsa-miR-1872

*******************AAGACAGAGTGCCACTTACTGA*************************************************** hsa-miR-1872*

....................................................TCTCAGTAAGTGGCACTCTGTC.................. t0026917 1

...................AAGACAGAGTGCCACTTACTGA................................................... t0051305 1

>hsa-miR-1873 chr4 77114043 77114135 - mfe=-60.800

GAAGTAAGCATCACTCAGCATAAACTGCATGCCTGCACACCAGGCATGCAGTGATGTGCAGGCATGCAGTTTATGTTAAGTGATGCTTTCTTT

((((.((((((((((.(((((((((((((((((((((((.((.(....)..)).))))))))))))))))))))))).)))))))))).))))

******************CATAAACTGCATGCCTGCACA****************************************************** hsa-miR-1873

..................CATAAACTGCATGCCTGCACA...................................................... t0012400 2

..................CATAAACTGCATGCCTGCACACC.................................................... t0036845 1

................AGCATAAACTGCATGCCTGCA........................................................ t0053789 1

>hsa-miR-1874 chr4 100137557 100137638 + mfe=-43.700

GAACAATCTAAAGGACCTGTACTAGGTTTAACATGTTGAGCATTACTCATGTTAGACCTAGTACACGTCCTTTAGATTCTTT

(((.((((((((((((.((((((((((((((((((............)))))))))))))))))).)))))))))))).)))

***************************************************TTAGACCTAGTACACGTCCTT********** hsa-miR-1874

...................................................TTAGACCTAGTACACGTCCTT.......... q0020136 3

...................................................TTAGACCTAGTACACGTCCT........... q0035629 2

...................................................TTAGACCTAGTACACGTCC............ t0055022 1

>hsa-miR-1875 chr4 113538009 113538100 + mfe=-46.600

GAATTATTAGGCTAGTGCAAAGGTAATTGCGGTTTTTGCTATTGCTTTTAAATGGCAAAAACTGCAGTTACTTTTGCACCAACCAATATTTC

(((.((((.((...(((((((((((((((((((((((((((((.......)))))))))))))))))))))))))))))...)))))).)))

*******************AAGGTAATTGCGGTTTTTGCT**************************************************** hsa-miR-1875

...................AAGGTAATTGCGGTTTTTGCT.................................................... q0019038 3

..................AAAGGTAATTGCGGTTTTTGCT.................................................... q0071293 1

>hsa-miR-1876 chr4 153629928 153630019 - mfe=-43.500

TCCTCTTGAGGTACCTGAATTACCAAAAGCTTTATGTATTCTGAAGTTATTGAAAATAAGAGCTTTTGGGAATTCAGGTAGTTCAGGAGTGA

((((((((((.((((((((((.(((((((((((.(((.(((..........))).)))))))))))))).)))))))))).)))))))).))

************************************************************AGCTTTTGGGAATTCAGGTAG*********** hsa-miR-1876

************ACCTGAATTACCAAAAGCTTT*********************************************************** hsa-miR-1876*

............................................................AGCTTTTGGGAATTCAGGTAG........... q0012974_t0021581 5_1

............................................................AGCTTTTGGGAATTCAGGTAGT.......... q0063445 1

............ACCTGAATTACCAAAAGCTTT........................................................... q0067337 1

............ACCTGAATTACCAAAAGCTT............................................................ t0027797 1

>hsa-miR-1877 chr4 160269404 160269496 + mfe=-58.160

TCTTCACTTTAAAGAGTGGCAAAGTCTTTCCATATGTGTAACAGACATACATACATACATATGGAAAGACTTTGCCACTCTTGAAAGTGAAGA

((((((((((.((((((((((((((((((((((((((((...............)))))))))))))))))))))))))))).))))))))))

***********************************************************TATGGAAAGACTTTGCCACT************** hsa-miR-1877

...........................................................TATGGAAAGACTTTGCCACT.............. q0032060 2

...........................................................TATGGAAAGACTTTGCCACTCTT........... q0078826 1

>hsa-miR-1878 chr4 174425875 174425970 + mfe=-45.900

AGAAAAATGTTAGGGTGGTGCAAAAGTGATCGTGGTTTTTGCAATTTTTTAATGACAAAAACCACAATTACTTTTGCACCAACCTAACCTTGTTTT

........((((((.(((((((((((((((.((((((((((..(((....)))..)))))))))).))))))))))))))).))))))........

********************CAAAAGTGATCGTGGTTTTTG******************************************************* hsa-miR-1878

....................CAAAAGTGATCGTGGTTTTTG....................................................... q0011788_t0010163 6_3

....................CAAAAGTGATCGTGGTTTTTGCA..................................................... q0024815 2

....................CAAAAGTGATCGTGGTTTTT........................................................ q0053746 1

..................TGCAAAAGTGATCGTGGTTTTT........................................................ q0077854 1

....................CAAAAGTGATCGTGGTTTTTGC...................................................... q0081740 1

>hsa-miR-1879 chr5 14879033 14879126 - mfe=-45.900

TAGGACCCTTACTTGGATCTGCAATTAGTATTTTAATCATAGATTGTATTTAGTTAGTTTTTAATACTAACTGCAGATTCAAGTGAGGGTTCTA

(((((((((((((((((((((((.((((((((..((.....(((((....)))))...))..)))))))).)))))))))))))))))))))))

****************************************************************TACTAACTGCAGATTCAAGTG********* hsa-miR-1879

................................................................TACTAACTGCAGATTCAAGTG......... q0029817 2

................................................................TACTAACTGCAGATTCAAGTGA........ q0103342 1

>hsa-miR-1880 chr5 24946240 24946323 + mfe=-43.000

TATTAGGTTGGTGGAAAGGTAATTGCGGTTTTTGCTATTAAAAGTAATGGCAAATCCTGCAATTACGTTTGCACCAATCCAATA

((((.((((((((.(((.((((((((((..((((((((((....))))))))))..)))))))))).))).)))))))).))))

***************AAGGTAATTGCGGTTTTTGCT************************************************ hsa-miR-1880

...............AAGGTAATTGCGGTTTTTGCT................................................ q0019038 3

..............AAAGGTAATTGCGGTTTTTGCT................................................ q0071293 1

>hsa-miR-1881 chr5 55907988 55908078 + mfe=-45.200

TTTATATTAGGCTGGTGCAAAAGTAATTGTGGTTTTTGTGATTGAAAGTAATGGCAAAAATTGCAATTACTTTTGCACTAACCTAAATAAA

.((((.(((((.(((((((((((((((((..((((((((.((((....)))).))))))))..))))))))))))))))).))))))))).

******************AAAAGTAATTGTGGTTTTTGTG*************************************************** hsa-miR-1881

..................AAAAGTAATTGTGGTTTTTGTG................................................... q0008077_t0050435 9_1

..................AAAAGTAATTGTGGTTTTTGTGA.................................................. q0079945 1

>hsa-miR-1882 chr5 70954349 70954445 + mfe=-52.800

AGTAGCTGGGATTACAGGCATGTGCTACCCTGCTTGGCTTGTTTTAAAAATGCATTTCAAGGCAGACGCGGTGGCTCACGCCTGTAATCCCAGCACT

(((.(((((((((((((((.((.((((((((((((((..(((.........)))..)))).))))....)))))).)).))))))))))))))))))

*******************ATGTGCTACCCTGCTTGGCTTG******************************************************** hsa-miR-1882

...................ATGTGCTACCCTGCTTGGCTTG........................................................ q0031567_t0022060 2_1

>hsa-miR-1883 chr5 89080979 89081064 - mfe=-38.900

GTTATTAAGTTGGTGCAAAAGTAATTGTGGTTTTTGTAATTTTTTTAATGACAAAACCATGATTATTTTTGCACCAACCTAATATC

..(((((.(((((((((((((((((..(((((((.((.(((.....))).)))))))))..))))))))))))))))).)))))..

***************CAAAAGTAATTGTGGTTTTTGTA************************************************ hsa-miR-1883

...............CAAAAGTAATTGTGGTTTTTGTA................................................ q0035339 2

................AAAAGTAATTGTGGTTTTTGTA................................................ q0062996 1

>hsa-miR-1884 chr5 170746268 170746366 - mfe=-57.600

GAGGAATGAACAGTTAAATTATAACATGTCCATATTATGGGTTAGTTGTGGACACATACTAACGCATAATATGGACATGTTATAATTTAACTGTTCCTT

.......((((((((((((((((((((((((((((((((.((((((.(((....))))))))).))))))))))))))))))))))))))))))))...

***********************************************************TAACGCATAATATGGACATGTT****************** hsa-miR-1884

...........................................................TAACGCATAATATGGACATGTT.................. q0019973_t0007868 3_4

............................................................AACGCATAATATGGACATGTTA................. q0031880_t0023930 2_1

...........................................................TAACGCATAATATGGACATGTTA................. q0046184_t0025375 1_1

...........................................................TAACGCATAATATGGACATGTTAT................ q0047824 1

...........................................................TAACGCATAATATGGACATGT................... q0070992_t0015402 1_2

............................................................AACGCATAATATGGACATGT................... q0077095 1

...........................................................TAACGCATAATATGGACATG.................... t0010235 3

...........................................................TAACGCATAATATGGACA...................... t0055552 1

>hsa-miR-1885 chr6 2799256 2799348 - mfe=-61.300

AGTTTCCATGATAGGGAAACCAGGCAAGAAATATTGTCTCCTCAAGTTGCGACGAGACAGTAGTTCTTGCCTGGTTTCTCTATCATGGAGTCT

((.(((((((((((((((((((((((((((.(((((((((.((.......)).))))))))).))))))))))))))))))))))))))).))

*******************************************************GACAGTAGTTCTTGCCTGGTTT**************** hsa-miR-1885

.......................................................GACAGTAGTTCTTGCCTGGTTT................ q0019106 3

......................................................AGACAGTAGTTCTTGCCTGGTT................. q0091751 1

>hsa-miR-1886 chr6 20675393 20675464 - mfe=-54.600

GCTGGTGCAAAAGTAATCGTGGTCTTTGCCATTACTTTCAATGGCAAAGACCACGATTACTTTTGCACCAAC

..(((((((((((((((((((((((((((((((......)))))))))))))))))))))))))))))))..

*******************************************GCAAAGACCACGATTACTTTT******** hsa-miR-1886

********AAAAGTAATCGTGGTCTTTGCC****************************************** hsa-miR-1886*

...........................................GCAAAGACCACGATTACTTTT........ q0047217 1

........AAAAGTAATCGTGGTCTTTGC........................................... q0047871 1

............................................CAAAGACCACGATTACTTTTGCA..... q0086699 1

........AAAAGTAATCGTGGTCTTTGCC.......................................... q0093877 1

>hsa-miR-1887 chr6 24927000 24927094 + mfe=-58.200

GTCGTTTATTCAATAAATTGATCCCTTATGTTCTCCTAGATCTACTATGACTCTAGGAGAACATAAGGGATCAATTTATTGAGTAACATAATGAC

((((((((((((((((((((((((((((((((((((((((((......)).))))))))))))))))))))))))))))))))))....))))))

******************************************************AGGAGAACATAAGGGATCAAT******************** hsa-miR-1887

......................................................AGGAGAACATAAGGGATCAAT.................... q0023456 2

......................................................AGGAGAACATAAGGGATCAATT................... q0067671 1

>hsa-miR-1888 chr6 33773891 33773980 + mfe=-43.000

TTCCTGTCCCCAGTTTTCAGGTGTGGAAACTGAGGCAGGAGGCAGTGAAGTAACTTGCTCAGGTTGCACAGCTGGGAAGTGGAGCAGGGA

(((((((..(((.((..(((.((((.((.(((((.((((..((......))..))))))))).)).)))).)))..)).))).)))))))

****************TCAGGTGTGGAAACTGAGGCA***************************************************** hsa-miR-1888

................TCAGGTGTGGAAACTGAGGCA..................................................... q0036802 2

................TCAGGTGTGGAAACTGAGGCAGG................................................... t0022832 1

................TCAGGTGTGGAAACTGAGGCAG.................................................... t0029336 1

...............TTCAGGTGTGGAAACTGAGGCA..................................................... t0031447 1

>hsa-miR-1889 chr6 82532186 82532261 - mfe=-50.700

TGTGCTCCAGTACATATAAAGAGACTTATTAAGATGATCTTTTCTTAATAAGTCTCTTTATATGTACTGGAGCCCG

.(.(((((((((((((((((((((((((((((((........))))))))))))))))))))))))))))))).).

*****TCCAGTACATATAAAGAGACTT************************************************* hsa-miR-1889

******************************************************TCTTTATATGTACTGGAGCCC* hsa-miR-1889*

.....TCCAGTACATATAAAGAGACTT................................................. q0038805 1

......................................................TCTTTATATGTACTGGAGCCC. q0078520 1

..TGCTCCAGTACATATAAAGAGA.................................................... q0112472 1

>hsa-miR-1890 chr6 91079183 91079272 + mfe=-54.400

GAACCTTAGTAAGGTTTGGATAGATGCAATAAAGTATGTCCACAGCTGAAAGGACATACTTTATTGCATGTATCCAAACCTTACTAATTC

.....(((((((((((((((((.((((((((((((((((((..........)))))))))))))))))).)))))))))))))))))...

**************TTTGGATAGATGCAATAAAGTA****************************************************** hsa-miR-1890

..............TTTGGATAGATGCAATAAAGTA...................................................... q0017123_t0002358 3_16

...............TTGGATAGATGCAATAAAGTA...................................................... q0030830_t0018077 2_1

.........TAAGGTTTGGATAGATGCAAT............................................................ q0041294_t0010802 1_2

..............TTTGGATAGATGCAATAAAGTATG.................................................... q0068696_t0052111 1_1

...............TTGGATAGATGCAATAAAGTATG.................................................... q0077585 1

..............TTTGGATAGATGCAATAAAGT....................................................... t0003501 10

.......AGTAAGGTTTGGATAGATGCA.............................................................. t0006875 4

......................................TCCACAGCTGAAAGGACA.................................. t0006973 4

..........AAGGTTTGGATAGATGCAATAA.......................................................... t0008086 3

.........TAAGGTTTGGATAGATGCAATA........................................................... t0009870 3

.........TAAGGTTTGGATAGATGCA.............................................................. t0010398 2

.....TTAGTAAGGTTTGGATAGATG................................................................ t0010847 2

..............TTTGGATAGATGCAATAAAGTATGT................................................... t0014133 2

......TAGTAAGGTTTGGATAGATGCA.............................................................. t0015518 2

.............GTTTGGATAGATGCAATAAAGTA...................................................... t0024767 1

...............TTGGATAGATGCAATAAAGT....................................................... t0026910 1

........GTAAGGTTTGGATAGATGCA.............................................................. t0031892 1

..............TTTGGATAGATGCAATAA.......................................................... t0038877 1

...........AGGTTTGGATAGATGCAATAA.......................................................... t0039617 1

..............TTTGGATAGATGCAATAAAG........................................................ t0049463 1

>hsa-miR-1891 chr6 108204187 108204270 - mfe=-45.400

TATTGGGTGGGTGCAAAAGTAATTGCGGTTTTTGCTATTAGTTTCAATGGTAAAAACCGTGACTACTTCTGCACCAACCTAGTA

((((((((.((((((.(((((.(..((((((((((((((......))))))))))))))..).))))).)))))).))))))))

**************************************************TAAAAACCGTGACTACTTCT************** hsa-miR-1891

..................................................TAAAAACCGTGACTACTTCT.............. q0032953_t0030004 2_1

..................................................TAAAAACCGTGACTACTTCTGC............ q0107065 1

..................................................TAAAAACCGTGACTACTTC............... t0021056 1

..................................................TAAAAACCGTGACTACTT................ t0029729 1

>hsa-miR-1892 chr6 120378014 120378110 + mfe=-43.100

ATCAGACTGAAACTACACTTTAAGGGGACCAAAGAGATATATAGATATCAGCTACCTATATACCTGTTCGGTCTCTTTAAAGTGTAGTTTAACTGAT

(((((....(((((((((((((((((((((.((.((.(((((((...........))))))).)).)).)))))))))))))))))))))..)))))

*********************************************************ATATACCTGTTCGGTCTCTTT******************* hsa-miR-1892

.........................................................ATATACCTGTTCGGTCTCTTT................... q0101753_t0014266 1_2

>hsa-miR-1893 chr6 124336490 124336578 - mfe=-44.600

ACGATTTAGGTTGGTGCAAAAGTAATTGCAGTTTTTGCCATTACTTTCAATGTCAAAACTACAGTTACTTTTGCACCAACCTAAATAGT

...(((((((((((((((((((((((((.((((((.(.((((......)))).))))))).)))))))))))))))))))))))))...

*****************************************************CAAAACTACAGTTACTTTTGCA************** hsa-miR-1893

.....................................................CAAAACTACAGTTACTTTTGCA.............. q0005665 14

.....................................................CAAAACTACAGTTACTTTTGC............... q0015689 4

>hsa-miR-1894 chr6 135559044 135559111 + mfe=-26.170

AAAATAGGACTTTTGAAGGAAGAGTTTTTTTTCACATTTTCACACTTTTCCTTCAAAAGTCATATTTT

((((((.((((((((((((((((((..................)))))))))))))))))).))))))

****TAGGACTTTTGAAGGAAGAGTT****************************************** hsa-miR-1894

....TAGGACTTTTGAAGGAAGAGTT.......................................... q0028824 2

....TAGGACTTTTGAAGGAAGAGTTT......................................... q0053574 1

>hsa-miR-1895 chr6 143914769 143914859 + mfe=-45.500

TTCACATGAAAAATTAAAGTTTACTGCATAGTGCTTGCTTAATAATTTGTTCAGCAAGCAATATGCAATAAACTTTAACTTTTCATGTGAG

.(((((((((((.((((((((((.((((((.((((((((.(((.....))).)))))))).)))))).)))))))))).))))))))))).

**************TAAAGTTTACTGCATAGTGCTT******************************************************* hsa-miR-1895

..............TAAAGTTTACTGCATAGTGCTT....................................................... q0011982 5

...............AAAGTTTACTGCATAGTGCTT....................................................... q0035644 2

...............AAAGTTTACTGCATAGTGCTTG...................................................... q0043106 1

................AAGTTTACTGCATAGTGCTTG...................................................... q0090335 1

>hsa-miR-1896 chr6 154546929 154547022 - mfe=-50.800

AGAGAAAGTTTTCAGTGTAACTCAACATTTGAAGTGTACTTGCCCTTGGACCAAGCAATTCTTCAAATGTTGAGATACACTGAAAACTTTCTCT

(((((((((((((((((((.((((((((((((((....((((.(....)..)))).....)))))))))))))).)))))))))))))))))))

**********TTCAGTGTAACTCAACATTTGA************************************************************** hsa-miR-1896

**************************************************************TCAAATGTTGAGATACACTGA*********** hsa-miR-1896*

..........TTCAGTGTAACTCAACATTTGA.............................................................. q0020437 3

..............................................................TCAAATGTTGAGATACACTGA........... q0035257 2

..............................................................TCAAATGTTGAGATACACTGAAA......... q0054226 1

................................................................AAATGTTGAGATACACTGAAA......... q0068493 1

>hsa-miR-1897 chr7 5378022 5378112 - mfe=-44.300

GTTGCGTTCCAGCCTGGGCAACAAGAGTGAAGTTCTGTCTCAAAAAAAAAGGAAATGGGGTCTCACTCTGTTGCTCAGGCTGGTCTTGAAC

(((.((..(((((((((((((((.((((((..((((((.((..........)).))))))..)))))))))))))))))))))...)))))

*******************AACAAGAGTGAAGTTCTGTCT*************************************************** hsa-miR-1897

...................AACAAGAGTGAAGTTCTGTCT................................................... q0074866 1

...................AACAAGAGTGAAGTTCTGTCTCA................................................. t0034530 1

...................AACAAGAGTGAAGTTCTGT..................................................... t0058256 1

>hsa-miR-1898 chr7 91093023 91093118 + mfe=-38.400

GTGACTATTGGGTTTGGAGCAAAAGTAATTGCGTTTTTTCCCATCACTTTCAATGGCAAAAACTGCAATTACTTTTGAACCAACCGGATAGCTTAT

(((((((((.((((.((..((((((((((((((.(((((.((((........)))).))))).))))))))))))))..)))))).))))).))))

********************************************************CAAAAACTGCAATTACTTTTGA****************** hsa-miR-1898

........................................................CAAAAACTGCAATTACTTTTGA.................. q0015755 4

.......................................................GCAAAAACTGCAATTACTTTTGA.................. q0039647 1

>hsa-miR-1899 chr7 98317209 98317286 + mfe=-24.400

GTAACAGTAACTTTTATTCTCATTTTCCTTTTCTCTACCTTGTAGAGAAGCAAAGTGATGAGTAATACTGGCTGGAGC

((..((((.....(((((((((((((...(((((((((...))))))))).))))))).)))))).....))))..))

**************************************************CAAAGTGATGAGTAATACTGGCT***** hsa-miR-1899

.................................................GCAAAGTGATGAGTAATACTGGCTGG... q0100473 1

..................................................CAAAGTGATGAGTAATACTG........ t0041629 1

..................................................CAAAGTGATGAGTAATACTGGCT..... t0052378 1

.................................................GCAAAGTGATGAGTAATACTGGCT..... t0053256 1

>hsa-miR-1900 chr7 101471879 101471943 + mfe=-26.700

GGCTGGTGTCAAACTCCTAGCTCAGGTGATCCGCCTAGTGGTTAGGATTTGGCGCTATCTCTCCC

((.((((((((((.((((((((((((((...))))).).)))))))))))))))))).)).....

**************************************TGGTTAGGATTTGGCGCT********* hsa-miR-1900

....................................AGTGGTTAGGATTTGGCG........... q0015925_t0026070 4_1

....................................AGTGGTTAGGATTTGGCGCT......... q0024504_t0025357 2_1

......................................TGGTTAGGATTTGGCGCT......... q0032830_t0006839 2_4

...................................TAGTGGTTAGGATTTGGC............ q0065843 1

...................................TAGTGGTTAGGATTTGGCG........... q0098528_t0042814 1_1

....................................AGTGGTTAGGATTTGGCGC.......... t0033572 1

...................................TAGTGGTTAGGATTTGGCGCT......... t0036900 1

>hsa-miR-1901 chr7 138379372 138379461 - mfe=-29.000

GGATAATAGGACAAGACAAATTACAGATTGTCTCAGAGAAAACAAATGAGTTACTCTCTCGGACAAGCTGTAGGTCCTACCTAAATGTCC

(((((.((((....(((....(((((.((((((.(((((.(((......)))..))))).)))))).))))).)))...))))..)))))

******************AATTACAGATTGTCTCAGAGAA************************************************** hsa-miR-1901

******************************************************TCTCTCGGACAAGCTGTAGGT*************** hsa-miR-1901*

..................AATTACAGATTGTCTCAGAGAA.................................................. q0002101_t0007264 52_4

..................AATTACAGATTGTCTCAGAGA................................................... q0002258_t0004346 47_7

..................AATTACAGATTGTCTCAGAGAAA................................................. q0002925_t0041767 35_1

..................AATTACAGATTGTCTCAGAG.................................................... q0009467_t0001714 7_25

....................TTACAGATTGTCTCAGAGAAA................................................. q0009488_t0024018 7_1

...................ATTACAGATTGTCTCAGAGAAA................................................. q0010551 6

....................TTACAGATTGTCTCAGAGAA.................................................. q0014883 4

...................................................TACTCTCTCGGACAAGCTGTAGGT............... q0015987 4

......................................................TCTCTCGGACAAGCTGTAGGT............... q0016845 4

...................ATTACAGATTGTCTCAGAGAA.................................................. q0017821 3

..................AATTACAGATTGTCTCAGAGAAAA................................................ q0022381 3

.................AAATTACAGATTGTCTCAGAG.................................................... q0029076_t0014971 2_2

..................AATTACAGATTGTCTCAGAGAAAAC............................................... q0029650 2

....................................................ACTCTCTCGGACAAGCTGTAGGT............... q0032402 2

........................................................TCTCGGACAAGCTGTAGGTC.............. q0036312_t0049240 2_1

......................................................TCTCTCGGACAAGCTGTAGGTC.............. q0047090 1

................................................AGTTACTCTCTCGGACAAGC...................... q0047785 1

........................................................TCTCGGACAAGCTGTAGGT............... q0049462 1

........................................................TCTCGGACAAGCTGTAGG................ q0062080 1

........................................................TCTCGGACAAGCTGTAGGTCC............. q0066221 1

...................................................TACTCTCTCGGACAAGCTGTAGG................ q0074999 1

.....................................................CTCTCTCGGACAAGCTGTAG................. q0076636 1

.................AAATTACAGATTGTCTCAGAGA................................................... q0080639 1

....................TTACAGATTGTCTCAGAGA................................................... q0083761 1

...................ATTACAGATTGTCTCAGAGA................................................... q0085708 1

................................................AGTTACTCTCTCGGACAAGCT..................... q0086223_t0011879 1_2

...................ATTACAGATTGTCTCAGAGAAAA................................................ q0088597 1

.................AAATTACAGATTGTCTCAGAGAA.................................................. q0092242 1

..................AATTACAGATTGTCTCAG...................................................... q0092355_t0005075 1_6

...................................................TACTCTCTCGGACAAGCTGTAG................. q0103912 1

.....................TACAGATTGTCTCAGAGA................................................... q0106581 1

..................AATTACAGATTGTCTCAGA..................................................... t0012004 2

....................TTACAGATTGTCTCAGAGAAAA................................................ t0013001 2

...................................................TACTCTCTCGGACAAGCT..................... t0030637 1

..................................................TTACTCTCTCGGACAAGCT..................... t0034402 1

......................................................TCTCTCGGACAAGCTGTA.................. t0040974 1

....................TTACAGATTGTCTCAGAG.................................................... t0055596 1

> hsa-miR-1902 chr8 6590092 6590174 + mfe=-44.900

AGAAACTGCTGAAGCTGCCATGTCTAAGAAGAAAACTTTGGAGAAAAATTTTCTTCTTAGACATGGCAACGTCAACAGTTTCT

((((((((.(((.(.((((((((((((((((((((.(((.....))).)))))))))))))))))))).).))).))))))))

*************************************************TTTCTTCTTAGACATGGCAAC************* hsa-miR-1902

.................................................TTTCTTCTTAGACATGGCAACG............ q0024943_t0027895 2_1

................................................TTTTCTTCTTAGACATGGCA............... q0093792 1

.................................................TTTCTTCTTAGACATGGCAAC............. t0007713 4

.................................................TTTCTTCTTAGACATGGCAA.............. t0015189 2

..................................................TTCTTCTTAGACATGGCAAC............. t0016078 2

..................................................TTCTTCTTAGACATGGCAACGT........... t0020562 1

.................................................TTTCTTCTTAGACATGGCA............... t0029129 1

.....................................................TTCTTAGACATGGCAACGT........... t0050155 1

................................................TTTTCTTCTTAGACATGGCAAC............. t0054961 1

..................................................TTCTTCTTAGACATGGCAACG............ t0058035 1

>hsa-miR-1903 chr8 12629112 12629190 - mfe=-61.000

GCTTTTAAAATGGAGCTGGCCAAAAAGCAGGCAGAGACTTTAAAAGCGTCTCTGCCTGCTTTTTGGCCAGCTCCGTTTT

......((((((((((((((((((((((((((((((((.........))))))))))))))))))))))))))))))))

*******************CCAAAAAGCAGGCAGAGACTT*************************************** hsa-miR-1903

...................CCAAAAAGCAGGCAGAGACTT....................................... q0019361 3

>hsa-miR-1904 chr8 69201988 69202068 - mfe=-38.800

TAGGTTCATGCAAAAGTAGTTGTGGTTTTGCCATTACTTTCAATGGATGGCAAAAACAGCAATTACTTTTGCACCAACCTA

((((((..(((((((((((((((..((((((((((..........))))))))))...)))))))))))))))..))))))

**********CAAAAGTAGTTGTGGTTTTGC************************************************** hsa-miR-1904

..........CAAAAGTAGTTGTGGTTTTGC.................................................. q0027191 2

..........CAAAAGTAGTTGTGGTTTTGCC................................................. q0102813 1

>hsa-miR-1905 chr8 78041555 78041645 - mfe=-43.400

GGATATACATACATGTACACACACATGTCATCCACACACATACATATATATATGTTTGTATGGATATGTGTGTGTATGTGTGTGTATACAC

...((((((((((((((((((((((((((....(((.(((((........))))).)))...))))))))))))))))))))))))))...

******************************************************TTTGTATGGATATGTGTGTGTA*************** hsa-miR-1905

......................................................TTTGTATGGATATGTGTGTGTA............... q0023501 2

.......................................................TTGTATGGATATGTGTGTGTAT.............. q0063149 1

......................................................TTTGTATGGATATGTGTGTGTAT.............. t0029677 1

>hsa-miR-1906 chr8 96154315 96154400 - mfe=-70.300

GAGGGAAAGCAGGCCAACCTCGAGGATCTCCCCAGCCTTGGCGTTCAGGTGCTGAGGAGATCGTCGAGGTTGGCCTGCTTCCCCTC

(((((.(((((((((((((((((.(((((((.((((((((.....)))).)))).))))))).))))))))))))))))).)))))

****************************************************TGAGGAGATCGTCGAGGTTGG************* hsa-miR-1906

***************AACCTCGAGGATCTCCCCAGCC************************************************* hsa-miR-1906*

....................................................TGAGGAGATCGTCGAGGTTGG............. q0023898 2

....................................................TGAGGAGATCGTCGAGGTTGGC............ q0036708 2

...............AACCTCGAGGATCTCCCCAGCC................................................. q0068392 1

>hsa-miR-1907 chr8 101784353 101784448 - mfe=-34.600

TTTGTAATTCTTTACTGTGGAATAGCTCAGAATGTCAGTTCTGTTTTAAGTAACAGAATTGATAACTGAGCAAGGAAACGTAATTTGGATTATAAA

.(((((((((.((((.((......((((((..(((((((((((((......))))))))))))).))))))......))))))...))))))))).

********************AATAGCTCAGAATGTCAGTT******************************************************** hsa-miR-1907

....................AATAGCTCAGAATGTCAGTT........................................................ q0019077 3

.....................ATAGCTCAGAATGTCAGTT........................................................ q0093619 1

>hsa-miR-1908 chr8 104236015 104236096 + mfe=-58.500

TTTGGGGTGATGGGTGGGGCAATGGGATCAGGTGCCTCAAAGGGCATCCCACCTGATCCCACAGCCCACCTGTCACCCCAAA

.(((((((((((((((((.(..(((((((((((((((....)))).....)))))))))))..)))))))))))))))))).

************GGTGGGGCAATGGGATCAGGT************************************************* hsa-miR-1908

............GGTGGGGCAATGGGATCAGGT................................................. q0029197 2

............GGTGGGGCAATGGGATCAGGTG................................................ q0064842 1

>hsa-miR-1909 chr8 125903395 125903487 + mfe=-54.600

GTGTACCACAGTGTCTATTTAGCCAATTGTCCATCTTTAGCTATTCTGAATGCCTAAAGATAGACAATTGGCTAAATAGAAATTGTGGTACAT

((((((((((((.((((((((((((((((((.((((((((.(((.....))).)))))))).)))))))))))))))))).))))))))))))

******************TTAGCCAATTGTCCATCTTTAG***************************************************** hsa-miR-1909

*******************************************************AAAGATAGACAATTGGCTAAAT**************** hsa-miR-1909*

..................TTAGCCAATTGTCCATCTTTAG..................................................... q0017615_t0023703 3_1

.......................................................AAAGATAGACAATTGGCTAAAT................ q0036560 2

.......................................................AAAGATAGACAATTGGCTAAA................. q0090560 1

>hsa-miR-1910 chr8 126606302 126606393 + mfe=-47.520

GATTTATTAGGTTGGTGCAAAAGTTATTGTGGTTTTTGCTATTACTTTCAATGCAAAAACTACAATTACTTTTGCACCAATCTAATACTATC

(((.((((((((((((((((((((.((((((((((((((.............)))))))))))))).))))))))))))))))))))..)))

******************AAAAGTTATTGTGGTTTTTGCT**************************************************** hsa-miR-1910

..................AAAAGTTATTGTGGTTTTTGCT.................................................... q0008722 8

>hsa-miR-1911 chr9 20401145 20401236 - mfe=-54.500

AAGGAACAGGGGACACTTGTAATGGAGAACACTAAGCTATGGACTGCTATGGACTGCTAGTGCTCTCCGTTACAAGTATCCCCTGTTACCTT

(((((((((((((.((((((((((((((.(((((((((((((....)))))).))..))))).)))))))))))))).))))))))).))))

*************CACTTGTAATGGAGAACACT*********************************************************** hsa-miR-1911

********************************************************CTAGTGCTCTCCGTTACAAGTA************** hsa-miR-1911*

.............CACTTGTAATGGAGAACACT........................................................... q0033172 2

........................................................CTAGTGCTCTCCGTTACAAGTA.............. t0040081 1

>hsa-miR-1912 chr9 67905119 67905217 + mfe=-59.100

GATATTTGAACCTCCTCCCGTGAATCACAAATGTCCTTAATAGCAATCCTTAAATGCCATTAAGGACATTTGTGATTGATGGGAGGAGGATGAAATATT

(((((((.(.(((((((((((.(((((((((((((((((((.(((.........))).))))))))))))))))))).))))))))))).).)))))))

**********************************************************ATTAAGGACATTTGTGATTGAT******************* hsa-miR-1912

..........................................................ATTAAGGACATTTGTGATTGAT................... q0022582 3

.........................................................CATTAAGGACATTTGTGATTGAT................... q0027932 2

.........................................................CATTAAGGACATTTGTGATTGA.................... q0032106 2

...........................................................TTAAGGACATTTGTGATTGAT................... q0067022 1

>hsa-miR-1913 chr9 85278238 85278330 - mfe=-34.300

CTTTCTGTTTCTAAGATAGAAAGTGTATGATGTACTTTCTGTTTCTAAGATAGAAAGTGTATGATGTACTTTCTGTTTCTACGAAACTTGGAG

((((..((((((((((((((((((((((.((((((((((((((.....)))))))))))))).))))))))))))))).)).)))))..))))

************************************************************ATGATGTACTTTCTGTTTCTACGA********* hsa-miR-1913

............................................................ATGATGTACTTTCTGTTTCTACGA......... q0016516 4

>hsa-miR-1914 chr9 88521663 88521746 - mfe=-43.600

TATTCAGTTGGTGCAAAAGTAATTGTGGTTTTTGTGATTACTTTTAATTGTAAAAACCTCAATTACTTTTGCACCAACCTAATA

((((..(((((((((((((((((((.((((((((..((((....))))..)))))))).)))))))))))))))))))..))))

**************AAAAGTAATTGTGGTTTTTGTG************************************************ hsa-miR-1914

..............AAAAGTAATTGTGGTTTTTGTG................................................ q0008077_t0050435 9_1

..............AAAAGTAATTGTGGTTTTTGTGA............................................... q0079945 1

>hsa-miR-1915 chr9 96612065 96612160 + mfe=-43.900

GTGCTGCAGGTGTTGGAGAGCAGTGTGTGTTGCCTGGGGACTGTGTGGACTGGTATCACCCAGACAGCTTGCACTGACTCCAGACCCTGCCGTCAT

((((.(((((..((((((..(((((((.((((.(((((.(((((....)).)))....))))).)))).))))))).))))))..))))).).)))

************************************************************CAGACAGCTTGCACTGACT***************** hsa-miR-1915

****************AGAGCAGTGTGTGTTGCCTGGG********************************************************** hsa-miR-1915*

............................................................CAGACAGCTTGCACTGACT................. q0052206 1

................AGAGCAGTGTGTGTTGCCTGGG.......................................................... q0079367 1

>hsa-miR-1916 chr9 113734202 113734277 + mfe=-21.500

GGGAAAAAAAAAAGGATTTGTCTTGTAGCCAGGATATTGTTTTAAAGAAAATCCTTTTTGTTTTTCCAGGTGGACC

(((((((.(((((((((((.((((.....(((....))).....))))))))))))))).))))))).........

***********************************************AAAATCCTTTTTGTTTTTCCAG******* hsa-miR-1916

GGGAAAAAAAAAAGGATTTGTCT***************************************************** hsa-miR-1916*

...............................................AAAATCCTTTTTGTTTTTCCAG....... q0031613 2

............................................AAGAAAATCCTTTTTGTTTTTCCA........ q0104515 1

GGGAAAAAAAAAAGGATTTGTCT..................................................... q0113372 1

>hsa-miR-1917 chr9 122531275 122531369 - mfe=-36.700

AGGGCTGCTAGATTTAATGGATCAAATCACAAGATGCCTAGTTAAATTTGAATTTTAAATTTAACTGGACATCTTGCATTTTATCTGGTAATCCT

(((..((((((((..((((..........(((((((.((((((((((((((...)))))))))))))).)))))))))))..))))))))..)))

***************************CACAAGATGCCTAGTTAAATTT********************************************** hsa-miR-1917

**********************************************************ATTTAACTGGACATCTTGCATT*************** hsa-miR-1917*

...........................CACAAGATGCCTAGTTAAATTT.............................................. q0055944 1

..........................................................ATTTAACTGGACATCTTGCAT................ q0098519 1

..........................................................ATTTAACTGGACATCTTGCATT............... t0055556 1

>hsa-miR-1918 chr9 130047040 130047130 - mfe=-49.800

GGCCCCTCCTTCTCAGCCCCAGCTCCCGCTCACCCCTGCCACGTCAAAGGAGGCAGAAGGGGAGTTGGGAGCAGAGAGGGGACCACGGGCT

(((((.((((((((.((((((((((((.((.....(((((.(......)..))))).)))))))))))).)).)))))))).....)))))

*****************************************************CAGAAGGGGAGTTGGGAGCAGA**************** hsa-miR-1918

.....................................................CAGAAGGGGAGTTGGGAGCAGA................ q0007603 10

.....................................................CAGAAGGGGAGTTGGGAGCAG................. q0030852 2

.....................................................CAGAAGGGGAGTTGGGAGC................... q0103277 1

.....................................................CAGAAGGGGAGTTGGGAGCA.................. q0103881 1

>hsa-miR-1919 chrX 1372805 1372890 + mfe=-38.000

CTCTGCCCCATCTCCACCTGGACCCAGCGTAGACAAAGAGGTGTTTCTACTCCATATCTACCTGGACCCAGTGTAGATGGGAGGAG

((((..(((((((.(((.(((..((((.(((((....(((.........)))....)))))))))..)))))).))))))).))))

***************ACCTGGACCCAGCGTAGACAAA************************************************* hsa-miR-1919

...............ACCTGGACCCAGCGTAGACAAAG................................................ q0035010 2

...............ACCTGGACCCAGCGTAGACAAA................................................. q0035148_t0042367 2_1

...............ACCTGGACCCAGCGTAGACAAAGA............................................... q0054046 1

...............ACCTGGACCCAGCGTAGACA................................................... q0106334_t0052007 1_1

...............ACCTGGACCCAGCGTAGACAA.................................................. q0106560 1

>hsa-miR-1920 chrX 6442696 6442783 + mfe=-46.600

TTTATTAGGTTAGTGCAAAAGTAACTGCGGTTTTTGTCACTGCTCTTCATGGCAAAAACTGCAGTCACTCTTGCACCAACCTAATAAA

.((((((((((.((((((.(((.((((((((((((((((.((.....)))))))))))))))))).))).)))))).)))))))))).

****************AAAAGTAACTGCGGTTTTTGT*************************************************** hsa-miR-1920

................AAAAGTAACTGCGGTTTTTGT................................................... q0031378 2

................AAAAGTAACTGCGGTTTTTGTCA................................................. q0081875 1

>hsa-miR-1921 chrX 7115401 7115497 + mfe=-32.400

GTAGGATAAATGACTCATCCTAGCTTGCCTGAGACTGTCCCAGTTTGAAAACTGGACCTCATCAGTCCTAGACACACTGGGATGTGGTTCACCCTAT

(((((......((((((((((((..((.(((.(((((..((((((....)))))).......))))).))).))..)))))))).))))...)))))

****************ATCCTAGCTTGCCTGAGACTGT*********************************************************** hsa-miR-1921

................ATCCTAGCTTGCCTGAGACTGT........................................................... q0031741 2

...............CATCCTAGCTTGCCTGAGACTG............................................................ q0034964 2

...............CATCCTAGCTTGCCTGAGACTGT........................................................... q0067437 1

>hsa-miR-1922 chrX 13535865 13535952 - mfe=-33.800

GGCACAGGCATTTTGTTCTAAGACTGCCAGGAGAAAGGGTCAGGCTGGCCTGAGCTTGTCTGCTGGGTTTAGAGGAGGAAAATTTGCC

((((......((((.(((((((....((((.(((.(((.(((((....))))).))).))).)))).))))))).)))).....))))

****************************************************AGCTTGTCTGCTGGGTTT****************** hsa-miR-1922

....................................................AGCTTGTCTGCTGGGTTT.................. q0029430 2

....................................................AGCTTGTCTGCTGGGTTTA................. q0066259 1

>hsa-miR-1923 chrX 14822869 14822952 + mfe=-30.900

TATTAGATTGGTGCAAAAGTAATTGCAGCATTTGCCATTATGTTTAACTGCAAATACCACAGTTACTTTTGCATGAACCTAATA

((((((.((.(((((((((((((((..(.((((((..(((....)))..)))))).)..))))))))))))))).)).))))))

***************************************************AAATACCACAGTTACTTTTGCA*********** hsa-miR-1923

**************AAAAGTAATTGCAGCATTTG************************************************** hsa-miR-1923*

...................................................AAATACCACAGTTACTTTTGCA........... q0026614 2

..............AAAAGTAATTGCAGCATTTG.................................................. t0030555 1

>hsa-miR-1924 chrX 18051877 18051942 - mfe=-27.300

CTTGATTCCTGGTGTGTAGCTGTACTTTTGTGACAGACTCTTAAGTGCAGAGCACCCAGGCTCAGG

(((((..(((((.((((..((((((((..(.(.....).)..)))))))).))))))))).)))))

***********GTGTGTAGCTGTACTTTT************************************* hsa-miR-1924

...........GTGTGTAGCTGTACTTTT..................................... q0027878_t0009474 2_3

>hsa-miR-1925 chrX 49662010 49662104 + mfe=-45.000

TGTCCTGGTTCCCCCTCTCTAATCCTTGCTACCTGGGTGAGAGTGCTTTCTGAATGCAGTGCACCCAGGCAAGGATTCTGCAAGGGGGAGTGACA

.(((....((((((((..(.(((((((((...(((((((..(.(((.........))).).))))))))))))))))..)..)))))))).))).

*********************************************************AGTGCACCCAGGCAAGGATTCT**************** hsa-miR-1925

.........................................................AGTGCACCCAGGCAAGGATTCT................ q0055271_t0036213 1_1

..........................................................GTGCACCCAGGCAAGGATTC................. t0056055 1

>hsa-miR-1926 chrX 55494652 55494741 + mfe=-44.300

AATGTGGTAGATATATGCACGATATAGGTATGTATGTATGTATGTATAAAAGCAGGGCAGTATATATATCGTGCATATATCTACCACATT

((((((((((((((((((((((((((.((((...(((.(((.(......).)))..))))))).))))))))))))))))))))))))))

******************************************************************TATCGTGCATATATCTACCACAT* hsa-miR-1926

*ATGTGGTAGATATATGCACGAT******************************************************************* hsa-miR-1926*

..................................................................TATCGTGCATATATCTACCACAT. q0036201 2

.ATGTGGTAGATATATGCACGAT................................................................... q0085601_t0027608 1_1

..TGTGGTAGATATATGCACGATA.................................................................. t0036894 1

.....GGTAGATATATGCACGATAT................................................................. t0037166 1

>hsa-miR-1927 chrX 139835992 139836053 - mfe=-29.500

GCCTTCTCTTCCCAGTTCTTCTTGGAGTCAGGAAAAGCTGGGTTGAGAGGAGCAGAAAAGAA

((((((((..(((((((.((((((....)))))).)))))))..)))))).)).........

********************************AAAAGCTGGGTTGAGAGGA*********** hsa-miR-1927

................................AAAAGCTGGGTTGAGAGGA........... q0015108_t0048178 4_1

.................................AAAGCTGGGTTGAGAGGA........... q0073035 1

...............................GAAAAGCTGGGTTGAGAGGA........... q0093351 1

>hsa-miR-1928 chrX/Y 1372805 1372890 + mfe=-38.000

CTCTGCCCCATCTCCACCTGGACCCAGCGTAGACAAAGAGGTGTTTCTACTCCATATCTACCTGGACCCAGTGTAGATGGGAGGAG

((((..(((((((.(((.(((..((((.(((((....(((.........)))....)))))))))..)))))).))))))).))))

***************ACCTGGACCCAGCGTAGACAAAG************************************************ hsa-miR-1928

...............ACCTGGACCCAGCGTAGACAAAG................................................ q0035010 2

...............ACCTGGACCCAGCGTAGACAAA................................................. q0035148_t0042367 2_1

...............ACCTGGACCCAGCGTAGACAAAGA............................................... q0054046 1

...............ACCTGGACCCAGCGTAGACA................................................... q0106334_t0052007 1_1

...............ACCTGGACCCAGCGTAGACAA.................................................. q0106560 1

>hsa-miR-1929 chr10 5769057 5769155 + mfe=-46.400

TTTATTGTGAAATATGTCATTAATATGTACTGACAAAGCGTATCTGTGTAATAAATATGCTTTTTGTCAGTACATGTTAATGGTATATTTCATAACAAA

....(((((((((((..((((((((((((((((((((((((((.(((...))).)))))))..)))))))))))))))))))..)))))))))))....

*******************TTAATATGTACTGACAAAGCGT********************************************************** hsa-miR-1929

********************************************************ATGCTTTTTGTCAGTACATGTTA******************** hsa-miR-1929*

...................TTAATATGTACTGACAAAGCGT.......................................................... q0013923 4

........................................................ATGCTTTTTGTCAGTACATGTTA.................... q0112946 1

>hsa-miR-1930 chr10 6234161 6234250 + mfe=-78.100

CTGTTCCGGGCATCACCTCCCACTGCAGAGCCTGGGGAGCCGGACAGCTCCCTTCCCAGGCTCTGCAGTGGGAACTGATGCCTGGAACAG

(((((((((((((((..((((((((((((((((((((((..(((....)))))))))))))))))))))))))..)))))))))))))))

***************CCTCCCACTGCAGAGCCTGGGGA**************************************************** hsa-miR-1930

*******************************************************CCAGGCTCTGCAGTGGGAA**************** hsa-miR-1930*

...............CCTCCCACTGCAGAGCCTGGGGA.................................................... q0064097 1

.......................................................CCAGGCTCTGCAGTGGGAA................ t0037694 1

>hsa-miR-1931 chr10 12212749 12212839 - mfe=-49.000

CCAAGTACTATTAGGTTGGTGCAAAAGTAACTGCGGTTTTTGAGAAGTAATTGAAAACCGCAATTACTTTTGCAGCAACCTAATATTTTGG

(((((...((((((((((.(((((((((((.(((((((((..(.......)..))))))))).))))))))))).)))))))))).)))))

**********************AAAAGTAACTGCGGTTTTTGA************************************************ hsa-miR-1931

......................AAAAGTAACTGCGGTTTTTGA................................................ t0007561 4

.....................CAAAAGTAACTGCGGTTTTTGA................................................ t0050418 1

>hsa-miR-1932 chr10 35408528 35408618 - mfe=-32.720

TGTAAGCAGGTCTAATAAGAATTTCTTTTTCTTCACAATTATGAAAGAAAAGAAATTGTGAAGAAAGAAATTCTTACTAGTTTTGCTGTCA

....((((((.(((.((((((((((((..(((((((((((.............))))))))))))))))))))))).))).))))))....

*******************************************************TTGTGAAGAAAGAAATTCTTA*************** hsa-miR-1932

****************AAGAATTTCTTTTTCTTCACA****************************************************** hsa-miR-1932*

.......................................................TTGTGAAGAAAGAAATTCTTA............... q0015657 4

.......................................................TTGTGAAGAAAGAAATTCTTACT............. q0026144 2

.......................................................TTGTGAAGAAAGAAATTCT................. q0026491 2

.......................................................TTGTGAAGAAAGAAATTCTT................ q0033957 2

................AAGAATTTCTTTTTCTTCACA...................................................... q0038816_t0016871 1_1

................AAGAATTTCTTTTTCTTCACAATT................................................... q0042934 1

................AAGAATTTCTTTTTCTTCACAA..................................................... q0088161 1

......................................................ATTGTGAAGAAAGAAATTCTT................ q0101959 1

.......................................................TTGTGAAGAAAGAAATTCTTAC.............. q0103411 1

................AAGAATTTCTTTTTCTTCA........................................................ q0109528 1

>hsa-miR-1933 chr10 60609637 60609695 - mfe=-19.700

GCAAACAAAGTACACATGCCTAGGGAAGACAATCCATGAGGTAGTGTGTGTGTGTGTGT

(((.(((...(((((((((((..(((......)))...))))).))))))..))).)))

************************************TGAGGTAGTGTGTGTGTGT**** hsa-miR-1933

....................................TGAGGTAGTGTGTGTGTGT.... q0014038 4

>hsa-miR-1934 chr10 103351157 103351253 + mfe=-80.700

GATCATTATTCAGGCCGGTCCTGCAGAGAGGAAGCCCTTCTGCTTACAGGTATTGGAAGGGCTTCCTCTCTGCAGGACCGGCCTGAATAATGTAATC

...(((((((((((((((((((((((((((((((((((((((((....)))...)))))))))))))))))))))))))))))))))))))).....

********************************************************AAGGGCTTCCTCTCTGCAGGAC******************* hsa-miR-1934

........................................................AAGGGCTTCCTCTCTGCAGGAC................... q0019970_t0016063 3_2

.........................................................AGGGCTTCCTCTCTGCAGGAC................... q0059839 1

........................................................AAGGGCTTCCTCTCTGCAGGA.................... t0012792 2

.........................................................AGGGCTTCCTCTCTGCAGGACCG................. t0055630 1

>hsa-miR-1935 chr11 33155668 33155761 + mfe=-54.110

CAGTTTCTCTTCCATCCCATATCTTAACAGCTAATCTAGTAAATTCTATCTTCAGAAGATTTGCTGTTAAGATATGGGATGGAGGAGAAATCTG

(((((((((((((((((((((((((((((((.(((((.......(((......)))))))).)))))))))))))))))))))))))))).)))

************************************************************TTGCTGTTAAGATATGGGATGG************ hsa-miR-1935

............................................................TTGCTGTTAAGATATGGGATGGA........... q0071499 1

............................................................TTGCTGTTAAGATATGGGATGG............ q0088200 1

............................................................TTGCTGTTAAGATATGGGA............... t0021856 1

...........................................................TTTGCTGTTAAGATATGGGAT.............. t0026265 1

...........................................................TTTGCTGTTAAGATATGGGATG............. t0036053 1

.............................................................TGCTGTTAAGATATGGGAT.............. t0046244 1

.............................................................TGCTGTTAAGATATGGGATGGA........... t0052273 1

>hsa-miR-1936 chr11 63865878 63865974 + mfe=-78.700

GGCCCAGGCATGGGGTTGTGGTCTGAGGTCTTGGGCCATCAGTGATGTCACAACCAGATGGCCCAAGACCCCAGACCACAACCCCATGTCTGGTGTT

.((((((((((((((((((((((((.((((((((((((((.(((....))).....)))))))))))))).)))))))))))))))))))))).)).

******************TGGTCTGAGGTCTTGGGCCAT********************************************************** hsa-miR-1936

..................TGGTCTGAGGTCTTGGGCCAT.......................................................... q0022810 3

>hsa-miR-1937 chr11 68607215 68607306 + mfe=-54.900

AGGTTCTTGGAAACTGTGACTTTAAGGGAAATGGCGCACAGCAGACCCTGCAATCATGCCGTTTTGCTTGAAGTCGCAGTTTCCCAGGACCT

(((((((.((((((((((((((((((.(((((((((....((((...)))).....))))))))).)))))))))))))))))).)))))))

**************TGTGACTTTAAGGGAAATGGCG******************************************************** hsa-miR-1937

..............TGTGACTTTAAGGGAAATGGCG........................................................ q0014610_t0035423 4_1

>hsa-miR-1938 chr11 71460915 71461003 - mfe=-65.600

TTGGTACCCGGTGGCAAGGTGGATGCAATGTGACCTCAACTCTTGGTCCTCTGAGGTCACATTGTATCCACCTTACCACTGGGTACTAA

.(((((((((((((.(((((((((((((((((((((((((.....))....))))))))))))))))))))))).))))))))))))).

****************AGGTGGATGCAATGTGACCTCA*************************************************** hsa-miR-1938

................AGGTGGATGCAATGTGACCTCA................................................... q0026530 2

................AGGTGGATGCAATGTGACCTCAA.................................................. q0067115 1

>hsa-miR-1939 chr11 73787925 73788023 + mfe=-49.900

TATTAGGTCGGTGCAAAAGTAATTGCTGTTTTTGCCATTAAAAATAATGGCATTAAAAGTAATGGCAAAAACGGCAATGACTTTTGTACCAATCTAATA

((((((((.(((((((((((.(((((((((((((((((((....((((...))))....))))))))))))))))))).))))))))))).))))))))

*********************************************************************AACGGCAATGACTTTTGTA*********** hsa-miR-1939

.....................................................................AACGGCAATGACTTTTGTA........... q0023856 2

......................................................................ACGGCAATGACTTTTGTACC......... q0050990 1

.....................................................................AACGGCAATGACTTTTGTACCA........ q0077971 1

.....................................................................AACGGCAATGACTTTTGT............ q0112688 1

>hsa-miR-1940 chr11 120858367 120858465 + mfe=-51.700

CTGGAACAGGCTGAGTGGGTTGGTGGGGGCAGCATTCTCTGTAGATAGTTTCTAATTCCCCAGAGAAGGCTGCTCCTCACCACCCCACCAGACTCTCAG

((((...((.(((.(((((.(((((((((((((.(((((((..((...........))..))))))).))))).)))))))).)))))))).)).))))

***********************************************************CCAGAGAAGGCTGCTCCTCACCA***************** hsa-miR-1940

***********************TGGGGGCAGCATTCTCTGTAGA****************************************************** hsa-miR-1940*

...........................................................CCAGAGAAGGCTGCTCCTCACCA................. q0067684 1

.......................TGGGGGCAGCATTCTCTGTAGA...................................................... t0047120 1

>hsa-miR-1941 chr12 21562856 21562939 + mfe=-58.700

TTTTTCTCCCAGTCAAGAGTTACTAGAACTATTCAACCTTCAGCTGTGTTGAATAGTTTTAGTAACTCTTGACTGGGAGAAAAG

.(((((((((((((((((((((((((((((((((((((.......).)))))))))))))))))))))))))))))))))))).

**************AAGAGTTACTAGAACTATT*************************************************** hsa-miR-1941

**************************************************GAATAGTTTTAGTAACTCTTGA************ hsa-miR-1941*

..............AAGAGTTACTAGAACTATT................................................... q0062076 1

...................................................AATAGTTTTAGTAACTCTTGA............ q0071405 1

..................................................GAATAGTTTTAGTAACTCTTGA............ q0086858 1

..............AAGAGTTACTAGAACTATTCA................................................. q0095294 1

>hsa-miR-1942 chr12 29307836 29307923 + mfe=-62.300

AGCTGGACTGAGAGAAATTTATTCTTGGTAGGTTGTACATTCCTAAACATGTACAACGTACTAAGAATAAATTTCTCTCAGTCCAGCT

((((((((((((((((((((((((((((((.(((((((((........))))))))).))))))))))))))))))))))))))))))

***************AATTTATTCTTGGTAGGTTGT**************************************************** hsa-miR-1942

*****************************************************CAACGTACTAAGAATAAATTTC************* hsa-miR-1942*

...............AATTTATTCTTGGTAGGTTGT.................................................... q0085962 1

.....................................................CAACGTACTAAGAATAAATTTC............. q0091738 1

......................................................AACGTACTAAGAATAAATTTCT............ q0100922 1

>hsa-miR-1943 chr12 51271035 51271127 - mfe=-55.100

GAGAACCTGAGGATCTTGTTGAACTAGGGCAGGATCCTTTCTGTCCAGAAGAAAAGGGACCTGCCCTAGTTCAGTAGGATCCTTGTGTTCCTC

..((((.((((((((((((((((((((((((((.((((((((.......)).)))))).)))))))))))))))))))))))))).))))...

***************TTGTTGAACTAGGGCAGGAT********************************************************** hsa-miR-1943

...............TTGTTGAACTAGGGCAGGAT.......................................................... t0002920 12

...............TTGTTGAACTAGGGCAGGATCCT....................................................... t0004890 6

...............TTGTTGAACTAGGGCAGGATC......................................................... t0007402 4

...............TTGTTGAACTAGGGCAGGATCCTTT..................................................... t0009683 3

...............TTGTTGAACTAGGGCAGGATCCTT...................................................... t0013472 2

...............TTGTTGAACTAGGGCAGGATCC........................................................ t0016053 2

...............TTGTTGAACTAGGGCAGGA........................................................... t0038514 1

..................TTGAACTAGGGCAGGATCCT....................................................... t0049414 1

>hsa-miR-1944 chr12 64931121 64931211 + mfe=-30.700

GGTTAGCACAGAGTGGGAGCTCTAGAAAGATTGTTGACCAATCATCTTATTGACTAGACCATCTTTCTAGAGTATAACTATTTTGGACACC

(((...(.((((((((..(((((((((((((.(((...((((......))))....))).)))))))))))))....)))))))))..)))

*****************AGCTCTAGAAAGATTGTTGACC**************************************************** hsa-miR-1944

.................AGCTCTAGAAAGATTGTTGACC.................................................... q0012757 5

.................AGCTCTAGAAAGATTGTTGAC..................................................... q0066775_t0049763 1_1

>hsa-miR-1945 chr12 68264771 68264868 - mfe=-76.300

GTTTATAATAAACTGAAATATTTGGGACTGATCTTGATGTCTGCCAAAACCTTGGCAGACATCAAGATCAGTCCCAAATATTTCAGTTTATTATAGAC

(((((((((((((((((((((((((((((((((((((((((((((((....)))))))))))))))))))))))))))))))))))))))))))))))

**********************TGGGACTGATCTTGATGTCT******************************************************** hsa-miR-1945

****************************************************TGGCAGACATCAAGATCAGTC************************* hsa-miR-1945*

...................ATTTGGGACTGATCTTGATGTC......................................................... q0030887 2

......................TGGGACTGATCTTGATGTCTG....................................................... q0040089 1

......................TGGGACTGATCTTGATGTCT........................................................ q0102450_t0009588 1_3

....................TTTGGGACTGATCTTGATGTCT........................................................ q0111665 1

.....................TTGGGACTGATCTTGATGTCT........................................................ t0012105 2

.....................TTGGGACTGATCTTGATGTC......................................................... t0012341 2

....................................................TGGCAGACATCAAGATCAGTC......................... t0016886 1

..........................ACTGATCTTGATGTCTGCCAAA.................................................. t0022365 1

......................TGGGACTGATCTTGATGT.......................................................... t0024991 1

>hsa-miR-1946 chr12 70365967 70366062 - mfe=-39.250

GCTGCGTTTGCACTGCTTCTCCAAAACCACATTATAGGTACTAAACAACATTGTTTAGTACCTATAATGTGCTAGACTCCTGGCTGCTAGCGAGGT

(((.((((.(((..(((..........((((((((((((((((((((....))))))))))))))))))))..........)))))).)))).)))

*****************************************************TTTAGTACCTATAATGTGCTAG********************* hsa-miR-1946

****************************ACATTATAGGTACTAAACAA************************************************ hsa-miR-1946*

.....................................................TTTAGTACCTATAATGTGCTAG..................... q0026137 2

............................ACATTATAGGTACTAAACAA................................................ t0033897 1

>hsa-miR-1947 chr13 49468550 49468640 - mfe=-31.500

GATGGTTGGGTTTGGATTGTTGTACTTTTTTTTTTGTTCGTTGCATTTTTAGGAACAAAAAAAAAAGCCCAACCCTTCACACCACTTCATC

(((((...(((.((((..((((..(((((((((((((((.(.........).)))))))))))))))..))))..)))).)))...)))))

*****************TGTTGTACTTTTTTTTTTGTTC**************************************************** hsa-miR-1947

******************************************************ACAAAAAAAAAAGCCCAACCCT*************** hsa-miR-1947*

.................TGTTGTACTTTTTTTTTTGTTC.................................................... q0001736_t0030781 67_1

.................TGTTGTACTTTTTTTTTTGTT..................................................... q0002672_t0004042 39_8

......................................................ACAAAAAAAAAAGCCCAACCCT............... q0004108_t0031314 22_1

.................TGTTGTACTTTTTTTTTTGT...................................................... q0008511_t0016420 8_1

......................TACTTTTTTTTTTGTTCGTTGCATT............................................ q0023803 2

...................TTGTACTTTTTTTTTTGTTCGT.................................................. q0037913 2

......................................................ACAAAAAAAAAAGCCCAACC................. q0074511 1

.................TGTTGTACTTTTTTTTTTGTTCG................................................... q0079219 1

......................TACTTTTTTTTTTGTTCGTTG................................................ q0090095 1

......................................................ACAAAAAAAAAAGCCCAACCCTT.............. t0021252 1

.................TGTTGTACTTTTTTTTTTG....................................................... t0033392 1

................TTGTTGTACTTTTTTTTTTGTT..................................................... t0035516 1

>hsa-miR-1948 chr13 68650567 68650646 + mfe=-32.300

TTAGGTTGGTGTAAAAGTAATTGTGGTTTTTGAAATTATATTTAACAGTAAAAATCGTAATTGTGTTTGCACCAACCTAG

.((((((((((((((..(((((((((((((((...(((....)))...)))))))))))))))..)))))))))))))).

************AAAAGTAATTGTGGTTTTTGAA********************************************** hsa-miR-1948

............AAAAGTAATTGTGGTTTTTGAA.............................................. q0006517 12

............AAAAGTAATTGTGGTTTTTGAAA............................................. q0018932 3

>hsa-miR-1949 chr13 114028066 114028152 + mfe=-45.400

TTTATTAGTTTGGTGCAAAAGTAATTGCAGTTTTTGCTGTTGAACGTAGTGGTAAAACTGCAGTTATTTTTGCACCAACCTAATAGG

.(((((((.(((((((((((((((((((((((((.((..(((....)))..))))))))))))))))))))))))))).))))))).

****************************************************TAAAACTGCAGTTATTTTTG*************** hsa-miR-1949

....................................................TAAAACTGCAGTTATTTTTG............... q0012355_t0013052 5_2

....................................................TAAAACTGCAGTTATTTTTGC.............. q0022656 3

....................................................TAAAACTGCAGTTATTTTTGCA............. t0036090 1

>hsa-miR-1950 chr14 25711213 25711296 - mfe=-52.800

TATTAGGTTGGTGCAAAAGTAATTGTTGTTCTTGCCATTTAAAGTAATGGCAAAAACCACAATTACTTTTGCACCAACCTAATA

((((((((((((((((((((((((((.(((.((((((((......)))))))).))).))))))))))))))))))))))))))

**************AAAAGTAATTGTTGTTCTTGCC************************************************ hsa-miR-1950

..............AAAAGTAATTGTTGTTCTTGCC................................................ t0010085 3

>hsa-miR-1951 chr14 33535504 33535600 - mfe=-39.300

TGAGTGTGTGAGTGTGTGTGTGTGAGTGTGTATGCGTGTGTGTGTCTGTGAGCGCTCATGCGTGCACATCTTCAGTCATGTGCCCACTCCTGTACCA

.(.((..(.(((((.(..((((((((((((((((((((.((((........)))).)))))))))))))..)))..))))..).))))).)..))).

********TGAGTGTGTGTGTGTGAGTGTGTA***************************************************************** hsa-miR-1951

........TGAGTGTGTGTGTGTGAGTGTGTA................................................................. q0016680 4

........TGAGTGTGTGTGTGTGAGTGTGTAT................................................................ q0066783 1

>hsa-miR-1952 chr14 90236780 90236877 - mfe=-65.900

GGTATGTATCTGCTTTTCCGGAGTTGTAAGTGTTGACAATATCCAGAATGACATTGTCTTTGTCAACACTTACAACTCTGGAAAAGCAGATACATACC

(((((((((((((((((((((((((((((((((((((((..........(((...))).)))))))))))))))))))))))))))))))))))))))

********TCTGCTTTTCCGGAGTTGTAA********************************************************************* hsa-miR-1952

........TCTGCTTTTCCGGAGTTGTAA..................................................................... q0024162 2

..........TGCTTTTCCGGAGTTGTAAGTGT................................................................. q0079434 1

>hsa-miR-1953 chr14 94673994 94674091 - mfe=-53.400

GGTCTTGGGCCAGCTTCCCTGCCCTGCCTGTTTTCTCCTTTGTGATTTTATGAGAACAAAGGAGGAAATAGGCAGGCCAGGGAAACGATCTCTCTCCC

((....(((...(.(((((((.(((((((((((((((((((((..((....))..))))))))))))))))))))).))))))).)...)))...)).

********************GCCCTGCCTGTTTTCTCCTTTG******************************************************** hsa-miR-1953

***********************************************************AGGAGGAAATAGGCAGGCCAGG***************** hsa-miR-1953*

....................GCCCTGCCTGTTTTCTCCTTTG........................................................ q0066543 1

...........................................................AGGAGGAAATAGGCAGGCCAGG................. q0090862 1

...................TGCCCTGCCTGTTTTCTCCTT.......................................................... t0022489 1

..........................................................AAGGAGGAAATAGGCAGGCCA................... t0058614 1

>hsa-miR-1955 chr15 33451756 33451852 - mfe=-42.500

AGTATGACACCTCAAAGAAGCAATACTGTTACCTGAAATAGGCTGCGAAGATAACAGTATTTCAGATAACAGTATTACATCTTTGAAGTGTCATATT

((((((((((.(((((((.(.((((((((((.(((((((..((((.........))))))))))).)))))))))).).))))))).))))))))))

******************AGCAATACTGTTACCTGAAAT********************************************************** hsa-miR-1955

***********************************************************TTTCAGATAACAGTATTACAT***************** hsa-miR-1955*

..................AGCAATACTGTTACCTGAAATA......................................................... q0045785_t0024907 1_1

...........................................................TTTCAGATAACAGTATTACAT................. q0045984 1

.................AAGCAATACTGTTACCTGAAAT.......................................................... q0053371 1

..................AGCAATACTGTTACCTGAAAT.......................................................... t0008965 3

>hsa-miR-1956 chr15 49893167 49893237 + mfe=-25.100

TCCTTAGCTGGGTAACTTTGGGCAAACCGCTTGGTCTCTCAAGCCTAAGGTTCTTCAGCTATAAAATGGGA

((((((((((((.((((((((((..(((....))).......)))))))))).))))))))......))))

*CCTTAGCTGGGTAACTTTGGG************************************************* hsa-miR-1956

.CCTTAGCTGGGTAACTTTGGG................................................. q0032043_t0024734 2_1

>hsa-miR-1957 chr15 63798639 63798723 - mfe=-56.100

AAAAAAAGGGAAAGAAGAACTGTTGCATTTGCCCTGCACTCAGTTTGCACAGGGTAAATGCAATAGTTCTTCTTTCCCTTTTTTT

((((((((((((((((((((((((((((((((((((..(.......)..))))))))))))))))))))))))))))))))))))

*************GAAGAACTGTTGCATTTGCCCT************************************************** hsa-miR-1957

.............GAAGAACTGTTGCATTTGCCCT.................................................. q0034759 2

.............GAAGAACTGTTGCATTTGCC.................................................... t0041423 1

>hsa-miR-1958 chr15 86483293 86483370 + mfe=-36.400

TTGGCCATGGGCTGAGTGCTGCTGACACAGAGATGAAGAAGACAGTCCCTGTCCTCCAGGAGCACTCAGCCTGGTGAA

.(.((((..(((((((((((.(((..((((.(((..........))).))))....))).))))))))))))))).).

*********************************************TCCCTGTCCTCCAGGAGCA************** hsa-miR-1958

.............................................TCCCTGTCCTCCAGGAGCA.............. q0048651_t0011889 1_2

>hsa-miR-1959 chr15 87664537 87664627 - mfe=-32.300

GTGGCAAACTGGAGCATGTAGGCTGATGTTGATACTGGAGAAAGCATTACCAGGCCTCCAGGTTACTTAGCCTAGCTCTCCAATTTGTTTC

..((((((.(((((....((((((((.((.....((((((...((........))))))))...))))))))))...))))).))))))..

****************TGTAGGCTGATGTTGATACTGGA**************************************************** hsa-miR-1959

................TGTAGGCTGATGTTGATACTGGA.................................................... q0035143 2

................TGTAGGCTGATGTTGATACTGGAG................................................... q0091759 1

>hsa-miR-1960 chr15 88228177 88228256 - mfe=-30.620

GTTTTGGATTTTGGACTTTTTCAGATTTGGGGATATTTGCATTATACTTATCCTAAATCTGAAAGTCCAAAACCTGAAAT

((((..(.((((((((((..(((((((((((.(((.............)))))))))))))))))))))))).)..))))

**********TTGGACTTTTTCAGATTTGGG************************************************* hsa-miR-1960

.........TTTGGACTTTTTCAGATTTGGGGAT.............................................. q0027509 2

..........TTGGACTTTTTCAGATTTGGGGAT.............................................. q0033241 2

..........TTGGACTTTTTCAGATTTGGG................................................. q0037139 2

.........TTTGGACTTTTTCAGATTTGGGG................................................ q0055033_t0035924 1_1

..........TTGGACTTTTTCAGATTTGGGGA............................................... q0072545 1

>hsa-miR-1961 chr16 5750694 5750784 - mfe=-40.400

GGTTGGTGCAAAAGTAACTGCGGTTTTTGTCATTACTTTCAAATAACTTTCAATATAATGGCAAAACCCCACAATTACTTTTGCACCAACC

(((((((((((((((((.((.((.(((((((((((..((.(((....))).))..)))))))))))..)).)).)))))))))))))))))

*********AAAAGTAACTGCGGTTTTTGT************************************************************* hsa-miR-1961

.........AAAAGTAACTGCGGTTTTTGT............................................................. q0031378 2

.........AAAAGTAACTGCGGTTTTTGTCA........................................................... q0081875 1

>hsa-miR-1962 chr16 14902859 14902953 + mfe=-66.400

GCCCAGCCAGGATCACAGACGTTTAAATTACACTCCTTCTGCTGTGCCTTACAGCAGTAGAAGGGGTGAAATTTAAACGTCTGTGATCCTGGGGT

((((...((((((((((((((((((((((.(((((((((((((((........))))))))))))))).))))))))))))))))))))))))))

**********************************************************AGAAGGGGTGAAATTTAAACGT*************** hsa-miR-1962

********************GTTTAAATTACACTCCTTCTGC***************************************************** hsa-miR-1962*

..........................................................AGAAGGGGTGAAATTTAAACGT............... q0015860_t0024165 4_1

.........................................................TAGAAGGGGTGAAATTTAAACG................ q0038035_t0059229 2_1

....................GTTTAAATTACACTCCTTCTGC..................................................... q0075185 1

..........................................................AGAAGGGGTGAAATTTAAACG................ q0103460_t0048634 1_1

>hsa-miR-1963 chr16 16301510 16301604 + mfe=-66.400

GCCCAGCCAGGATCACAGACGTTTAAATTACACTCCTTCTGCTGTGCCTTACAGCAGTAGAAGGGGTGAAATTTAAACGTCTGTGATCCTGGGGT

((((...((((((((((((((((((((((.(((((((((((((((........))))))))))))))).))))))))))))))))))))))))))

**********************************************************AGAAGGGGTGAAATTTAAACGT*************** hsa-miR-1963

********************GTTTAAATTACACTCCTTCTGC***************************************************** hsa-miR-1963*

..........................................................AGAAGGGGTGAAATTTAAACGT............... q0015860_t0024165 4_1

.........................................................TAGAAGGGGTGAAATTTAAACG................ q0038035_t0059229 2_1

....................GTTTAAATTACACTCCTTCTGC..................................................... q0075185 1

..........................................................AGAAGGGGTGAAATTTAAACG................ q0103460_t0048634 1_1

>hsa-miR-1964 chr16 18413248 18413342 - mfe=-66.400

GCCCAGCCAGGATCACAGACGTTTAAATTACACTCCTTCTGCTGTGCCTTACAGCAGTAGAAGGGGTGAAATTTAAACGTCTGTGATCCTGGGGT

((((...((((((((((((((((((((((.(((((((((((((((........))))))))))))))).))))))))))))))))))))))))))

**********************************************************AGAAGGGGTGAAATTTAAACGT*************** hsa-miR-1964

********************GTTTAAATTACACTCCTTCTGC***************************************************** hsa-miR-1964*

..........................................................AGAAGGGGTGAAATTTAAACGT............... q0015860_t0024165 4_1

.........................................................TAGAAGGGGTGAAATTTAAACG................ q0038035_t0059229 2_1

....................GTTTAAATTACACTCCTTCTGC..................................................... q0075185 1

..........................................................AGAAGGGGTGAAATTTAAACG................ q0103460_t0048634 1_1

>hsa-miR-1965 chr16 21424868 21424962 - mfe=-46.400

GCCTCAAATTTAAGGAGGGACTCACTCACAGGATTGTGCAAATGCAAAGTTGGCTTTTGCATGACCCTGGGAGTAGGTGCCTCCTTAAATTTTGC

((...(((((((((((((.((..((((.((((.(..((((((.((.......)).))))))..).)))).))))..)).))))))))))))).))

******************GACTCACTCACAGGATTGTGCA******************************************************* hsa-miR-1965

..................GACTCACTCACAGGATTGTGCA....................................................... q0035555 2

...................ACTCACTCACAGGATTGTGCAA...................................................... q0071833 1

.....................TCACTCACAGGATTGTGCAAAT.................................................... t0025907 1

>hsa-miR-1966 chr16 29517998 29518092 - mfe=-46.400

GCCTCAAATTTAAGGAGGGACTCACTCACAGGATTGTGCAAATGCAAAGTTGGCTTTTGCATGACCCTGGGAGTAGGTGCCTCCTTAAATTTTGC

((...(((((((((((((.((..((((.((((.(..((((((.((.......)).))))))..).)))).))))..)).))))))))))))).))

******************GACTCACTCACAGGATTGTGCA******************************************************* hsa-miR-1966

..................GACTCACTCACAGGATTGTGCA....................................................... q0035555 2

...................ACTCACTCACAGGATTGTGCAA...................................................... q0071833 1

.....................TCACTCACAGGATTGTGCAAAT.................................................... t0025907 1

>hsa-miR-1967 chr17 23159457 23159546 + mfe=-46.700

TGCCAGAGCCGGAGCGTAGCTGTAGCTTTAGCAGAGCAGCAGCACCGAGCTGGCTGGCTCTGCCTACTGCTATTCTCCCAGCACCTGGCA

.(((((.((.((((.(((((.((((.....(((((((((((((.....))).))).))))))))))).))))).))))..))..))))).

****************TAGCTGTAGCTTTAGCAGAGC***************************************************** hsa-miR-1967

*************************************************************GCCTACTGCTATTCTCCC*********** hsa-miR-1967*

................TAGCTGTAGCTTTAGCAGAGC..................................................... t0033342 1

.............................................................GCCTACTGCTATTCTCCC........... t0052272 1

>hsa-miR-1968 chr17 24212791 24212881 - mfe=-46.700

GGCCTGCGAGGGAGCTGTAGAGCAGGGAGCAGGAAGCTGTGTGTGTCCAGCCCTGACCTGTCCTGTTCTGCCCCCAGCCCCTCACAGTGCT

((((((.(((((.((((((((((((((..((((..((((........)))))))).....)))))))))))....)))))))).))).)))

***************TGTAGAGCAGGGAGCAGGAAGCT***************************************************** hsa-miR-1968

...............TGTAGAGCAGGGAGCAGGAAGCT..................................................... q0013168_t0011981 5_2

...............TGTAGAGCAGGGAGCAGGAAGCTG.................................................... q0037727 2

...............TGTAGAGCAGGGAGCAGGAAG....................................................... q0059465 1

...............TGTAGAGCAGGGAGCAGGAAGCTGT................................................... q0074248 1

>hsa-miR-1969 chr17 37919746 37919837 + mfe=-35.800

TAGAGCAGGGGGATGGCAGAGCAAAATTCATGGCCTACAGCTGCCTCTTGCCAAACTGCACTGGATTTTGTGTCTCCCATTCCCCAGAGCTG

...(((.(((((((((.(((((((((((((.(((........)))...(((......))).)))))))))).))).)))))))))...))).

******AGGGGGATGGCAGAGCAAAA****************************************************************** hsa-miR-1969

......AGGGGGATGGCAGAGCAAAA.................................................................. q0058775_t0040254 1_1

......AGGGGGATGGCAGAGCAAAATT................................................................ t0026824 1

>hsa-miR-1970 chr17 38113628 38113690 - mfe=-27.400

TTGGGAGGGAAGACAGCTGGAGAGTATGGTCACAGCAGCATCCTCCTCTGTTTTCTTTCCTAG

.(((((((((((((((..((((.(.(((.(......).)))))))).))))))))))))))).

TTGGGAGGGAAGACAGCTGGAGAG*************************************** hsa-miR-1970

TTGGGAGGGAAGACAGCTGGAGAG....................................... q0065508 1

TTGGGAGGGAAGACAGCTGGAGA........................................ q0097354_t0041452 1_1

.TGGGAGGGAAGACAGCTGGAGAGTAT.................................... t0045128 1

>hsa-miR-1971 chr17 52323626 52323720 - mfe=-47.400

CTGTAGGTTCTGTCTTGGGCCACTTGGATCTGAAGGCTGCCCCTTTGCTCTCTGGGGTAGCCTTCAGATCTTGGTGTTTTGAATTCTTACTATAG

((((((.....((.(..(..((((.((((((((((((((((((..........)))))))))))))))))).))))..)..))).....))))))

*********************************************************TAGCCTTCAGATCTTGGTGTTT**************** hsa-miR-1971

*******************CCACTTGGATCTGAAGGCTGCC****************************************************** hsa-miR-1971*

.........................................................TAGCCTTCAGATCTTGGTGTTT................ q0001376_t0003626 93_9

...................CCACTTGGATCTGAAGGCTGCC...................................................... q0003158 31

.........................................................TAGCCTTCAGATCTTGGTGTT................. q0004472_t0014138 20_2

.........................................................TAGCCTTCAGATCTTGGTGT.................. q0015052 4

.........................................................TAGCCTTCAGATCTTGGTGTTTT............... q0016876 4

........................................................GTAGCCTTCAGATCTTGGTGTT................. q0036954 2

.........................................................TAGCCTTCAGATCTTGGTG................... q0039808 1

...................CCACTTGGATCTGAAGGCTG........................................................ q0047830 1

....................CACTTGGATCTGAAGGCTGCC...................................................... q0061734 1

.........................................................TAGCCTTCAGATCTTGGT.................... q0062966 1

...................CCACTTGGATCTGAAGGCTGC....................................................... q0098790 1

>hsa-miR-1972 chr17 64607295 64607374 - mfe=-41.240

TCTTTGGAACGATAGCAGCATGAACCTGTCTCACTGCAGAATTATTTTGAGACAGGCTTATGCTGCTATCCTTCAAAAGA

(((((.(((.(((((((((((((.(((((((((..............))))))))).))))))))))))).))).)))))

***********************************************TGAGACAGGCTTATGCTGCTA************ hsa-miR-1972

*************AGCAGCATGAACCTGTCTCACT********************************************* hsa-miR-1972*

...............................................TGAGACAGGCTTATGCTGCTA............ t0015989 2

...............................................TGAGACAGGCTTATGCTGCTAT........... t0022593 1

.............AGCAGCATGAACCTGTCTCACT............................................. t0058940 1

.................................................AGACAGGCTTATGCTGCTAT........... t0059695 1

>hsa-miR-1973 chr18 3875346 3875439 + mfe=-61.600

TGCCTACAGTGAATCCCTAGTGGTCAGAGGGCTTATGATATATTGTGAGAGCCATGTCATAAGCCTTTTGGCCACTAGGGATTCAATGTATGCA

.((.((((.((((((((((((((((((((((((((((((((...((....)).)))))))))))))))))))))))))))))))).)))).)).

*****************TAGTGGTCAGAGGGCTTATGAT******************************************************* hsa-miR-1973

.................TAGTGGTCAGAGGGCTTATGAT....................................................... q0034710 2

.................TAGTGGTCAGAGGGCTTATGA........................................................ q0111233 1

>hsa-miR-1974 chr18 61509554 61509637 + mfe=-32.600

TATTAGGCTGGTGCAAAAGTTATTGTGGTTTTTGCTATTAAAAGTAATATCAAAAATTAATATTACTTTTGCACCAACTTAATA

(((((((.((((((((((((.....(((((((((.(((((....))))).))))))))).....)))))))))))).)))))))

**************AAAAGTTATTGTGGTTTTTGCT************************************************ hsa-miR-1974

..............AAAAGTTATTGTGGTTTTTGCT................................................ q0008722 8

>hsa-miR-1975 chr18 69962307 69962405 - mfe=-52.300

GTTTATGTATTAGGTTGGTGCAAAAGTAATTGTGGTTTTTGTTACTGAAAGTAATGGCAAAAGCCGCAATTACTTTAGCACCAACCTAGTAGTATATAC

((.((((((((((((((((((.((((((((((((((((((((((.((....)).)))))))))))))))))))))).)))))))))))))).)))).))

********************CAAAAGTAATTGTGGTTTTTGTT******************************************************** hsa-miR-1975

**********************************************************AAAAGCCGCAATTACTTTA********************** hsa-miR-1975*

....................CAAAAGTAATTGTGGTTTTTGTT........................................................ t0021300 1

..........................................................AAAAGCCGCAATTACTTTA...................... t0034242 1

>hsa-miR-1976 chr19 7632588 7632685 - mfe=-46.000

CCTTGAAGACATGACTTCTGTCTCTTCTTGAGCTACTCTTTGACACACCCTGGACAAAGGGTAGCTTGAAAAGAGACAGAAACCATGTTATTGCCAGG

(((.(..((((((..(((((((((((.(..((((((((((((.((.....))..))))))))))))..).)))))))))))..))))))....).)))

********************TCTCTTCTTGAGCTACTCTTTG******************************************************** hsa-miR-1976

....................TCTCTTCTTGAGCTACTCTTTG........................................................ q0068128_t0014933 1_2

>hsa-miR-1977 chr19 12892128 12892223 + mfe=-58.300

CCCACACAAGGCCTATCTATCTAGATTCTTCTTGGCCTCTCTGAGCATGCATTCCTGAGACTCCAAGAAGAATCTAGACAGATAGGCCTTGCTGGG

((((..((((((((((((.((((((((((((((((..((((.(((......)))..))))..)))))))))))))))).)))))))))))).))))

**********************************************************GACTCCAAGAAGAATCTAGACA**************** hsa-miR-1977

..........................................................GACTCCAAGAAGAATCTAGACA................ q0034709 2

..........................................................GACTCCAAGAAGAATCTAGACAGA.............. q0055030 1

.......................................................TGAGACTCCAAGAAGAATCTA.................... q0072387 1

............................................................CTCCAAGAAGAATCTAGACAGA.............. q0082216 1

...........................................................ACTCCAAGAAGAATCTAGACAG............... q0093865 1

.......................................................TGAGACTCCAAGAAGAATCTAG................... t0016274 1

.......................................................TGAGACTCCAAGAAGAAT....................... t0029222 1

>hsa-miR-1978 chr19 12912283 12912369 + mfe=-36.300

GGGCTCTGCTCTCCACATTGGAGGGTGTGGAAGACATCTGGGCCAACTCTGATCTCTTCATCTACCCCCCAGGACTGGGACAAGCCC

(((((.((.((.(((..((((.(((((..(((((.(((.(((....))).))).)))))...))))).))))...))))))))))))

*****************TTGGAGGGTGTGGAAGACATC************************************************* hsa-miR-1978

.................TTGGAGGGTGTGGAAGACATC................................................. q0023910 2

.................TTGGAGGGTGTGGAAGACAT.................................................. q0095724 1

>hsa-miR-1979 chr19 60428648 60428739 - mfe=-42.400

TTGAGGAAGGGTCAGGCATGAGGAGGATCTGAAGGATTGGACTCAGGTTCGAAACCTCCACTTCCTCCTCATCTCCTACCCTCTCCACTCAG

....((((((((.(((.((((((((((..((.(((.((((((....))))))..))).))..))))))))))..)))))))).)))......

******************TGAGGAGGATCTGAAGGATTGG**************************************************** hsa-miR-1979

..................TGAGGAGGATCTGAAGGATTGG.................................................... q0044757_t0023740 1_1

.................ATGAGGAGGATCTGAAGGAT....................................................... q0095239 1

..................TGAGGAGGATCTGAAGGATTG..................................................... t0011848 2

>hsa-miR-1980 chr20 3139237 3139327 + mfe=-45.000

TGGTGCCAGGACTGAGTGGCCTTCTCAGGAACCAGAGCCTTTTGCCGAAAAAAGGTTTGGGATCCTGAGGCCAGACCAGTCAGGCAGTCCA

.((((((..(((((..(((((...((((((.((.(((((((((......))))))))).)).)))))))))))...))))).))))..)).

****************TGGCCTTCTCAGGAACCAGAGCCTT************************************************** hsa-miR-1980

................TGGCCTTCTCAGGAACCAGAGCCTT.................................................. q0030233 2

................TGGCCTTCTCAGGAACCAGAGCC.................................................... t0048149 1

>hsa-miR-1981 chr20 32100575 32100668 + mfe=-64.300

CTATGAAAGCCAGATTCAGCTTTCCCTTCAGAGCCTGGCTTTGGCATCTATGAAAGCCAGGCTCTGAAGGGAAAGTTGAATCTTGCTAGAGTGG

((((...(((.(((((((((((((((((((((((((((((((.(......).))))))))))))))))))))))))))))))).)))...))))

*******************CTTTCCCTTCAGAGCCTGGCTTT**************************************************** hsa-miR-1981

...................CTTTCCCTTCAGAGCCTGGCTTT.................................................... q0035224 2

....................TTTCCCTTCAGAGCCTGG........................................................ t0028358 1

....................TTTCCCTTCAGAGCCTGGCTT..................................................... t0038202 1

....................TTTCCCTTCAGAGCCTGGCT...................................................... t0049174 1

>hsa-miR-1982 chr20 37838122 37838220 - mfe=-48.800

TAGAAACTATTAGATTGCTGCAAAAGTCATTGTGGTTTTTGACATTGAAATTAAAGGCAAAAACCACAATGACTTTTGCACCAACCTAATGGTTTACTG

(((((((((((((.(((.(((((((((((((((((((((((...((.......))..))))))))))))))))))))))).))).)))))))))).)))

********************CAAAAGTCATTGTGGTTTTTGA********************************************************* hsa-miR-1982

....................CAAAAGTCATTGTGGTTTTTGA......................................................... q0004979 17

.....................AAAAGTCATTGTGGTTTTTGA......................................................... q0101324_t0046100 1_1

>hsa-miR-1983 chr22 21611152 21611212 - mfe=-20.840

CCCGTGAGCTGGACATCCTAGAGCTCTCCTTCAATCCCATGGCCTCTAGGGTACCCTGCTC

.....((((.((..(((((((((..(..............)..)))))))))..)).))))

**CGTGAGCTGGACATCCTAGAGCT************************************ hsa-miR-1983

..CGTGAGCTGGACATCCTAGAGCT.................................... q0018351 3

>hsa-miR-1984 chr22 29457541 29457630 + mfe=-39.400

GGAGGTGGTCGAGGGAATCTGAGAAGGCGCACAAGGTTTGTGTCCAATACAGTCCACACCTTGCGCTACTCAGGTCTGCTCGTGCCCTCC

(((((.(..((((.(.(((((((..((((((..((((..(((..(......)..))))))))))))).))))))).).))))..))))))

***************AATCTGAGAAGGCGCACAAGGTT**************************************************** hsa-miR-1984

********************************************************CACCTTGCGCTACTCAGGTCTG************ hsa-miR-1984*

...............AATCTGAGAAGGCGCACAAGGTT.................................................... q0029423 2

........................................................CACCTTGCGCTACTCAGGTCTG............ q0086217 1

>hsa-miR-1985 chr22 29886048 29886105 - mfe=-40.500

GCTGAAGCTCTAAGGTTCCGCCTGCGGGCAGGAAGCGGAGGAACCTTGGAGCTTCGGC

(((((((((((((((((((..((((.........)))).)))))))))))))))))))

************************************GGAGGAACCTTGGAGCTTCGGC hsa-miR-1985

....................................GGAGGAACCTTGGAGCTTCGG. q0029559 2

....................................GGAGGAACCTTGGAGCTTCGGC q0058457_t0013394 1_2

.....................................GAGGAACCTTGGAGCTTCGGC t0044752 1

>hsa-miR-1986 chr22 34061644 34061742 + mfe=-49.730

GCGCTGTCCTTCCTCTGGGGAGCAGGCTCCGGGGGACAGGGAAAAGCACACAAGGAACTTGTCCTCTAGGGCCTGCAGTCTCATGGGAGAGTGACATGC

(((.((((((((((.(((((.((((((((..(((((((((.................)))))))))..))))))))..))))).)))).)).)))))))

***********************************************************TGTCCTCTAGGGCCTGCAGTCT****************** hsa-miR-1986

...........................................................TGTCCTCTAGGGCCTGCAGTCT.................. q0003160 31

...........................................................TGTCCTCTAGGGCCTGCAGTC................... q0061484_t0013867 1_2

...........................................................TGTCCTCTAGGGCCTGCAGT.................... q0068108 1

>hsa-miR-1987 chr22 44535062 44535147 + mfe=-47.440

TTATTTCTGATACCCCAAATCTTGATCAGAAGCCTTGATCAGAAGCTAGGAAGGCTTCTGATCAAGATTTGTGGTGTCAAGAATAA

.((((..(((((((.(((((((((((((((((((((..............))))))))))))))))))))).)))))))..)))).

******************************************************CTTCTGATCAAGATTTGTGGTGT********* hsa-miR-1987

......................................................CTTCTGATCAAGATTTGTGGTGT......... t0010301 3

...................................................AGGCTTCTGATCAAGATTTGT.............. t0036676 1

......................................................CTTCTGATCAAGATTTGTGGTG.......... t0037110 1
